# The Induction of Disease Resistance by Scopolamine and the Application of *Datura* Extract Against Potato (*Solanum tuberosum* L.) Late Blight

**DOI:** 10.3390/ijms252413442

**Published:** 2024-12-15

**Authors:** Zhiming Zhu, Shicheng Liu, Yi Liu, Xinze Zhang, Zhiwen Shi, Shuting Liu, Zhenglin Zhu, Pan Dong

**Affiliations:** 1 School of Life Sciences, Chongqing University, Chongqing 401331, China; zhuzhiming@pumc.edu.cn (Z.Z.); 202418021101t@stu.cqu.edu.cn (S.L.); 20205847@cqu.edu.cn (Y.L.); 20205558@cqu.edu.cn (X.Z.); shi-zw23@mails.tsinghua.edu.cn (Z.S.); 502024240060@smail.nju.edu.cn (S.L.); 2 Institute of Medicinal Plant Development, Chinese Academy of Medical Sciences and Peking Union Medical College, Beijing 100193, China; 3 Hongshen Honors School, Chongqing University, Chongqing 401331, China; 4 Shenzhen International Graduate School, Tsinghua University, Shenzhen 518055, China; 5 Chongqing Key Laboratory of Biology and Genetic Breeding for Tuber and Root Crops, Southwest University, Chongqing 400716, China

**Keywords:** scopolamine, *Datura* extract, *Phytophthora infestans*, transcriptome analysis, resistance induction

## Abstract

Late blight, caused by *Phytophthora infestans*, is a devastating disease of potato. Our previous work illustrated that scopolamine, the main bioactive substance of *Datura* extract, exerts direct inhibitory effects on *P. infestans*, but it is unclear whether scopolamine and *Datura* extract can boost resistance to late blight in potato. In this study, *P*. *infestans* is used to infect scopolamine-treated potato pieces and leaves, as well as whole potatoes. We found that scopolamine-treated potato is resistant to *P*. *infestans* both in vitro and in vivo. The treatment of 4.5 g/L scopolamine reduces the lesion size of whole potato to 54% compared with the control after 20 d of the infection of *P. infestans*. The disease-resistant substance detection based on the kit method shows that scopolamine triggers the upregulation of polyphenoloxidase, peroxidase, superoxide dismutase activities, and H_2_O_2_ contents in potato tubers, and the decline of phenylalanine ammonia lyase and catalase activity. A total of 1682 significantly differentially expressed genes were detected with or without scopolamine treatment through high-throughput transcriptome sequencing and the DESeq2 software (version 1.24.0), including 705 upregulated and 977 downregulated genes. Scopolamine may affect the genes functioning in the cell wall, membrane and the plant-pathogen interaction. The addition of *Datura* extract could directly inhibit the mycelial growth of *P*. *infestans* on rye plate medium. In addition, *P*. *infestans* was found to be resistant to late blight in potato pieces treated with *Datura* extract. *Datura* extract can also be utilized in combination with the chemical fungicide Infinito in field experiments to lessen late blight symptoms and enhance potato yield. To our knowledge, this is the first study to detect the induction of disease resistance by scopolamine, and it also explores the feasibility of *Datura* extract in potato disease resistance.

## 1. Introduction

Plant diseases caused by bacteria, fungi, oomycetes, nematodes, and viruses pose a serious threat to human health and global food security. Efficient chemical pesticides become the main controlling method of plant diseases, however, they may lead to severe impacts on human health and environmental safety [1]. Furthermore, the single target of chemical pesticides are prone to result in the resistance of pathogens. Therefore, environmentally friendly biocontrol has gained a multitude of attention in recent years, which includes antagonistic microorganisms and active substances produced by microorganisms, animals, and plants. There has been some research on the prevention and control of potato late blight by bacteria (*Bacillus subtilis*, *Pseudomonas fluorescens*, lactic acid bacteria, and *Azospirillum brasilense*, etc.) [2,3,4,5], fungi (*Trichoderma viride*, *Trichoderma isolate* HNA14, and *Streptomyces* sp. FXP04.) [6,7,8], and active substances (*β*-aminobutyric acid, cyclic lipopeptides, surfactin, fengycin B, and melatonin) [9,10,11,12,13]. The specific mechanisms consist of direct antibacterial ones, competition for resources (nutrition, space), parasitism, and the induction of disease resistance [14]. Induced resistance in plants is a defense formed by prior infection or some chemical treatments, such as chitosan and salicylic acid, which leads to plant resistance to subsequent pathogens or parasite attacks [15]. Inducible resistance can be used for the disease prevention and green production. However, only a few biologically active substances (eugenol, rotenone, and pyrethrum) and biocontrol bacteria (*Bacillus subtilis*, *Pseudomonas aeruginosa*) [16,17] have accessed the market. Herein, there is an urgent need to continue the research on biocontrol.

The traditional applications of *Datura*, like anesthetic, are mainly attributed to the active substances, like tropane alkaloids, that it contains (e.g., anisodamine, scopolamine). Nowadays, in the medical field, *Datura* extract can be taken advantage of for the relief of asthma, cough, analgesia, and anesthesia [18,19]. In agriculture, *Datura* extract is well known for its potential antifungal activity in vitro and in vivo [20,21,22]. In addition, it imposes desirable influences on the control of pests and diseases (tobacco (*Nicotiana tabacum*) mosaic virus disease, and sheath blight disease) [23,24], and affects seedling growth [25,26]. However, its inhibitory effect on oomycetes has not been reported. Scopolamine, the main active substance of *Datura* extract, is a classic non-competitive cholinergic receptor inhibitor, widely applied in the treatment of various human diseases, including neurological diseases, respiratory diseases, and digestive diseases [27,28]. However, few reports of scopolamine are about the prevention and treatment of plant diseases, except for the control of blast and sheath blight diseases of rice [29]. Our previous work has confirmed that scopolamine can inhibit the growth of mycelia, sporangia germination and viability, and pathogenicity of *P. infestans*, but its mechanism of inducing potato resistance against late blight remains unclear [14].

The following works are carried out in this study. Firstly, in vitro and in vivo induction experiments were conducted to determine the ability of scopolamine to improve resistance to *P. infestans* in different varieties of potato. Then, the activities of phenylalanine ammonia (PAL), polyphenol oxidase (PPO), peroxidase (POD), superoxide dismutase (SOD), and catalase (CAT), as well as the content of H_2_O_2_, are detected in potato tuber treated with or without scopolamine. In addition, through the transcriptome sequencing of potato tuber under the treatment of scopolamine, the molecular mechanism of the induction of disease resistance of scopolamine is preliminarily unraveled. At last, *Datura* extract is chosen for in vitro and field experiments to explore its application potential in potato late blight control.

## 2. Results

### 2.1. Scopolamine Enhances Potato Resistance to Late Blight In Vitro and In Vivo

**Figure 1:**
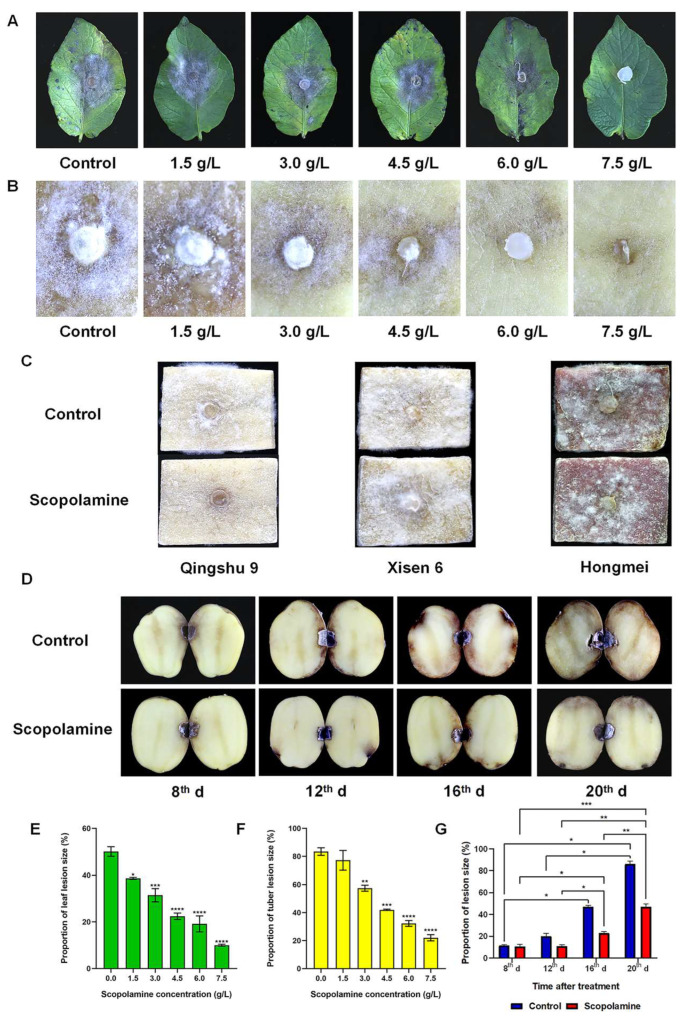
Scopolamine induces potatoes to resist late blight. Late blight symptoms of ‘Favorita’ potato leaves (**A**) and pieces (**B**) pretreated with different concentrations of scopolamine. (**C**) Late blight symptoms of ‘Qingshu 9’, ‘Xisen 6’, and ‘Hongmei’ potato pieces pretreated with scopolamine. (**D**) Symptoms of late blight in whole ‘Marco’ potatoes with or without scopolamine treatment. Proportion of lesion size of ‘Favorita’ potato leaves (**E**), pieces (**F**), and whole ‘Marco’ potatoes (**G**). d: days post inoculation with *P. infestans*. Tukey’s multiple comparisons test * *p* < 0.0332, ** *p* < 0.0021, *** *p* < 0.0002, **** *p* < 0.0001. 3 replicates per group.

To investigate whether scopolamine can induce plant disease resistance, the ‘Favorita’ potato pieces and leaves pretreated with scopolamine were inoculated with *P. infestans*. After 7 d post-inoculation (dpi), it was found that, in the control group, the leaves lost green around the inoculation site with obvious white mycelia (Figure 1A). The potato pieces grew white mycelia and rot with an unpleasant odor (Figure 1B). The higher the concentration of scopolamine, the smaller the spot area and the sparser the mycelia. Under the treatment of 7.5 g/L scopolamine, the proportion of the lesion areas of potato leaves and pieces was 10.02% and 22.05%, respectively, significantly lower than those of the control group (50.11% and 83.45%) (Figure 1E,F). In the control group, the potato pieces of ‘Qingshu 9’, ‘Xisen 6’, and ‘Hongmei’ all showed the phenotypes of browning and dense mycelia (Figure 1C). In the treatment group, there were no visible mycelia on ‘Qingshu 9’, and only the tissues at the inoculation point were browning. The mycelia growth of ‘Xisen 6’ and ‘Hongmei’ was also inhibited to varying degrees, and the mycelia became much sparser than in the control group.

In the whole potato pretreated scopolamine experiment, the occurrence process of late blight in transverse sections of ‘Marco’ potato was recorded from 8 to 20 dpi with *P. infestans* (Figure 1D). On the 8 dpi, there was no significant difference in the proportion of lesion area between the control and the treatment (11.95% and 10.71%), however, the degree of browning around the inoculation sites in the control group was more severe than that in the treatment group. On the 20 dpi, the mycelia around the inoculation sites in the control group grew vigorously, and the central and ring medullar region tissue was browning seriously (86.89%), while the proportion of lesion area in scopolamine treatment group was only 47.24% (Figure 1G).

### 2.2. Scopolamine Affects Disease-Resistant Substances of Potato Tubers

**Figure 2:**
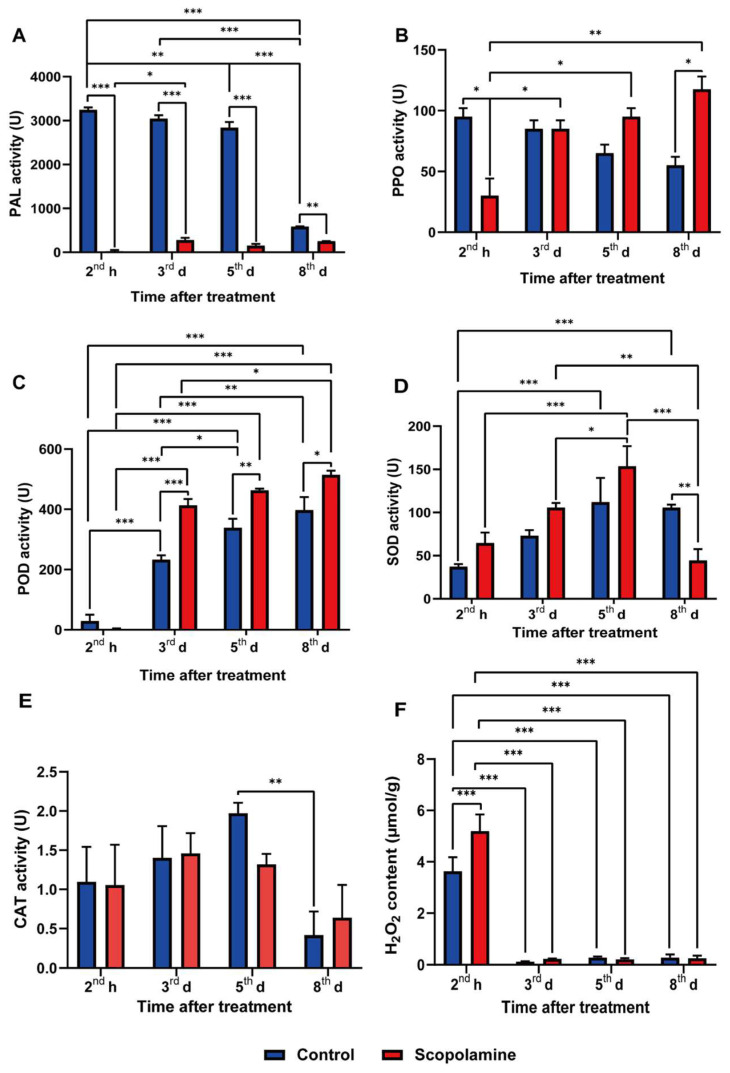
Change of disease-resistant substances in potato tubers with or without scopolamine treatment. (**A**) Phenylalanine ammonia (PAL) activity, (**B**) polyphenols oxidase (PPO) activity, (**C**) peroxidase (POD) activity, (**D**) superoxide dismutase (SOD) activity, (**E**) catalase (CAT) activity, (**F**) H_2_O_2_ content in control group and scopolamine-treated group. h: hours after scopolamine treatment. d: days post-inoculation with *P. infestans*. Three replicates per group. Values represent the means ± standard error of 3 independent samples (Tukey’s multiple comparisons test, * *p* < 0.0332, ** *p* < 0.0021, *** *p* < 0.0002).

To point out the effect of scopolamine on disease-resistant substances in potato tuber, the activities of PAL, PPO, POD, SOD, and CAT, and the content of H_2_O_2_, were detected after scopolamine treatment at the time of 2nd h, 3rd, 5th, 8th d after inoculation with *P. infestans* (Figure 2). The PAL activity in the treatment group (25–252 U) was significantly lower than control group (585–3245 U) at each time point (Figure 2A). The PPO activity of control group was continuously decreased from the 2nd h to 8th d (95–55 U), while in the treatment group, although it (30 U) was lower than that in the control group at the 2nd h, it increased a lot over time, and by the 8th d it had ascended to 117 U, which is 1.8 times that of the control group (Figure 2B). With the extension of treatment time, POD activity went up in both the control and treatment groups, while the treatment group upgraded more rapidly than the control group. From the 3rd to the 8th d, POD activity in the treated tubers (413–514 U) was significantly higher than that in the control group (232–397 U) (Figure 2C). The SOD activity of the control group (112 U) and the treatment group (154 U) reached a peak on the 5th d after inoculation, then the treatment group decreased more significantly than the control, and its activity was significantly lower than that of the control on the 8th d (Figure 2D). The change trend of CAT activity was similar to that of SOD, while the difference between the treatment and control groups was not significant at each time point (Figure 2E). The oxidative storm caused by endogenous H_2_O_2_ in the control group and the treatment group appeared at the 2nd h (3.8 and 5.2 μmol/g, respectively), with a significant difference (*p* < 0.0002) (Figure 2F). In both groups, the H_2_O_2_ levels plummeted (ranging from 0.1 to 0.3 µmol/g) during the 3rd–8th d, while it was not statistically significant between the treatment and control groups at the same time (*p* > 0.0332). Meanwhile, the mycelia on potato tubers pretreated with 200 μM exogenous H_2_O_2_ grew more sparsely than in the control (Supplementary Figure S2), suggesting that the increased level of H_2_O_2_ in tubers at the early stage of infection may help potato resist the disease. In a word, after treatment with scopolamine, PAL and CAT activity was significantly inhibited and SOD, PPO, and POD activities, and H_2_O_2_ content, increased.

### 2.3. RNA-Seq Analysis

**Figure 3:**
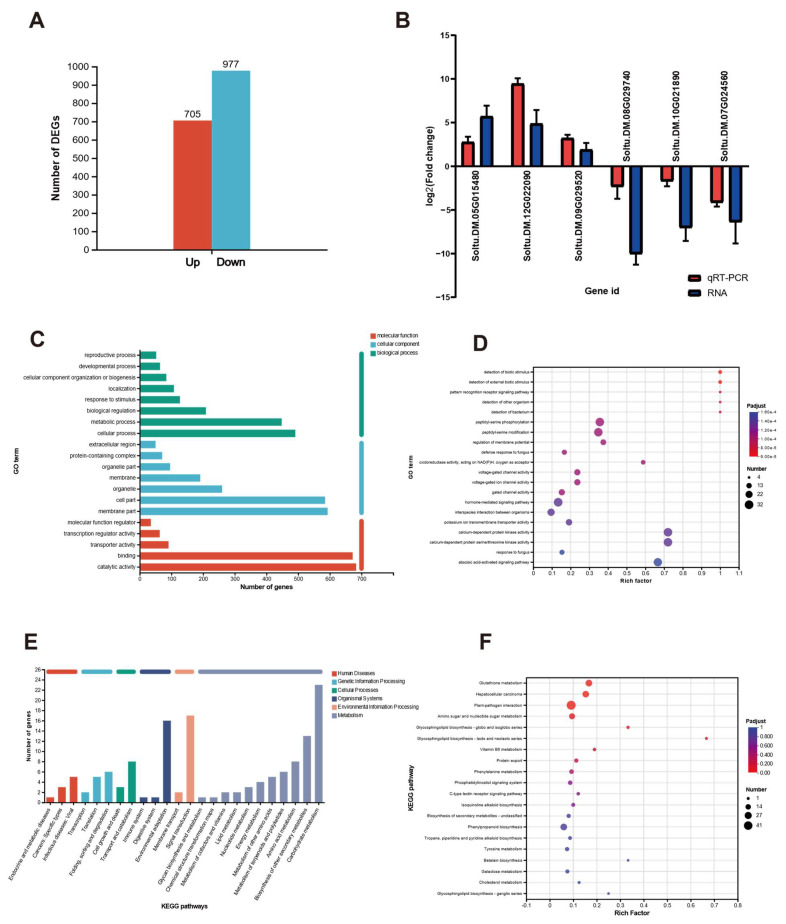
Main information of transcriptome sequencing in potato tubers under scopolamine treatment. (**A**) Distribution of differentially expressed genes. (**B**) qRT-PCR result. (**C**) GO annotation analysis diagram. The *X*–axis represents the number of genes compared to the secondary classification. (**D**) GO enrichment analysis. The horizontal axis represents the ratio of sample number of genes enriched in the rich factor (GO term) to the background number of annotated gene, and the color of the dot corresponds to different *p*-adjust ranges. (**E**) Histogram of KEGG. The *X*–axis is the number of genes annotated to the pathway. (**F**) KEGG enrichment analysis. The horizontal axis represents the ratio of rich factor (sample number of genes enriched in this pathway to background number of annotated genes). Three replicates per group.

Transcriptome analysis of potato tubers treated with or without scopolamine was performed to reveal the preliminary molecular mechanism of scopolamine-induced potato resistance to *P. infestans*. A total of 1682 significantly differentially expressed genes (DEGs) were observed, among which 977 were downregulated, and 705 were upregulated (Figure 3A, Supplementary Table S4). The most upregulated gene was Src Homology 3 (SH3) domain protein (65.14-fold), which associates with endocytosis and affects the immune pathway mediated by external signaling molecules. The most downregulated gene encodes a pseudo protein (1000-fold), and the second most downregulated gene was WRKY DNA-binding protein (600-fold), which has a variety of biological functions in plant growth, development, aging, and the regulation of stress resistance. The three most upregulated and three most downregulated DEGs were selected for the qRT-PCR experiment, and their expressions were consistent between transcriptome sequencing and qRT-PCR analysis (Figure 3B). Among the top 20 categories in GO annotation analysis, eight were biological processes (cellular process, metabolic process, etc.), five were molecular functions (catalytic activity, cell part, etc.), and seven were cellular components (cell part, organelle, etc.) (Figure 3C, Supplementary Table S5). In GO enrichment analysis, “detection of biotic stimulus” and “detection of external biotic stimulus” indicate the greatest enrichment (Figure 3D, Supplementary Table S5). KEGG annotation analysis shows that 11 of the top 20 pathways are related to metabolism, including carbohydrate metabolism, cofactor metabolism and vitamin metabolism, and sugar biosynthesis, etc. (Figure 3E, Supplementary Table S6). For KEGG enrichment analysis, glycosphingolipid biosynthesis-lacto and neolacto series had the highest enrichment (Figure 3F, Supplementary Table S6).

Since the cell wall, cell membrane, and plant–pathogen interaction are often concerned in inducing plant disease resistance, we pay more attention to these processes in the DEGs, GO terms, and KEGG pathways. There are 42 DEGs related to the cell wall, cell wall organization, and biogenesis in the transcriptome data (Supplementary Table S7). Upregulated genes consist of three polygalacturonase, two pectin lyase-like superfamily proteins, and four plant invertase/pectin methylesterase inhibitor superfamily genes, which can promote the synthesis of lignin, ethylene, and certain defense-related enzymes. Among the 517 membrane-associated DEGs, 37 genes are related to the leucine-rich repeat-containing protein family (the main immunoreceptor), and most of them are upregulated. In addition, 401 upregulated and 263 downregulated DEGs in plant resistance to pathogen invasion induced by scopolamine were found (Supplementary Table S8). After scopolamine treatment, PAMP-triggered immunity (PTI) and effector-triggered immunity (ETI) pathways were affected. The PTI pathway comprises 145 upregulated and 128 downregulated genes. The number of upregulated genes (256) in the ETI pathway is much more than in downregulated genes (135). The downregulated expression of MLP-like protein genes (Soltu.DM.09G007850, Soltu.DM.09G007770) can advance the level of ROS during pathogen invasion and enhance plant disease resistance in the ETI pathway.

### 2.4. Datura Extract Has Dual Effects Against Potato Late Blight

**Figure 4:**
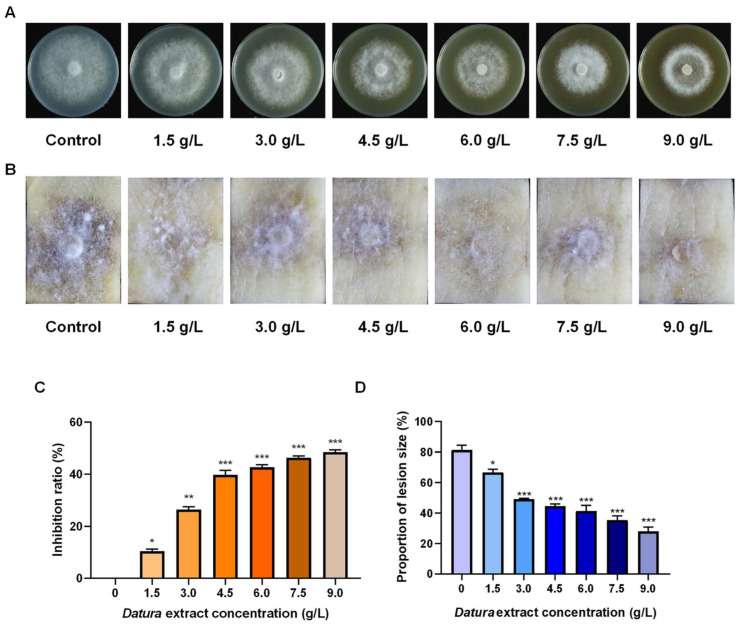
*Datura* extract has dual effects to resist potato late blight. (**A**) Growth state of *Phytophthora infestans* on the medium supplemented with different concentrations of *Datura* extract. (**B**) The growth of *Phytophthora infestans* on the potato pieces pretreated with different concentrations of scopolamine. (**C**) The inhibition ratio of *Datura* extract against *Phytophthora infestans*. (**D**) The proportion of lesion size of potato pieces. Three replicates per group. Dunnett’s multiple comparisons test, * *p* < 0.0332, ** *p* < 0.0021, *** *p* < 0.0002.

Scopolamine is the main active ingredient in *Datura* extract [30]. The anti-oomycete ability of *Datura* extract against *P. infestans* is tested in vitro. With the concentration of *Datura* extract increasing from 0 to 6.0 g/L, the mycelia become sparse and the colony diameter of *P. infestans* gradually descends from 6.7 to 4.0 cm. When the concentration exceeds 6.0 g/L, its inhibition effect on mycelia growth is not significantly improved with the increase of concentration (Figure 4A,C). In the induced resistance experiment, after 7 d of culture, there were thick white mycelia on the surface of the potato pieces with serious browning (81.43%). With the growth of *Datura* extract concentration, the proportion of lesion size gradually became lower, and the dense degree of mycelia decreased, exhibiting a dose-dependent effect. The proportion of the infected area in the tuber treated with 9 g/L scopolamine was only 28.13% at the 7th d (Figure 4B,D). Therefore, *Datura* extract is considered to have a dual effect on the control of potato late blight by both directly inhibiting the mycelia growth of *P. infestans* and inducing the resistance of potato against *P. infestans*.

### 2.5. Joint Effect of Datura Extract and Infinito in the Field Test

**Figure 5:**
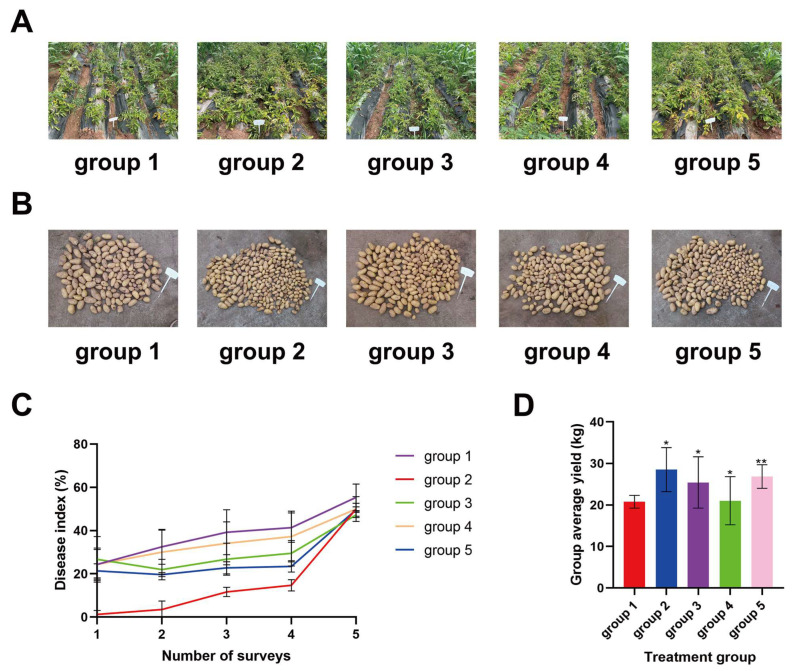
*Datura* extract could control late blight of potato and increase potato yield. (**A**) Late blight status of potato leaves at harvest. (**B**) Harvested potatoes. (The total length of the sign in the picture is 0.36 m) (**C**) Disease index of each treatment group. (The five surveys were given on 2, 6, 13, 19 April, and 5 May 2023). (**D**) Average yield of each treatment group. Treatment method: group 1. Control, treated with water; group 2. Infinito (1.5 mL); group 3. *Datura* extract (40 g); group 4. Infinito (0.15 mL); group 5. *Datura* extract (40 g) + Infinito (0.15 mL). (Tukey’s multiple comparisons test, * *p* < 0.0332, ** *p* < 0.0021.).

The field experiment on potato late blight control of *Datura* extract and Infinito was conducted in 2023 (Figure 5A,B). Starting from the discovery of the primary infection of late blight, the first spraying is performed. Before the second spraying, the first survey of the late blight occurrence of potato leaves is examined. In the first survey, each group is affected, and the disease index of the group 3 (*Datura* extract) is the highest (26.70%), while group 2 (1.5 mL Infinito) is the lowest (1.23%), indicating the control effect of *Datura* extract alone on foliar late blight disease is not ideal compared with the chemical fungicide. However, the combination of *Datura* extract and 0.15 mL Infinito (group 5) has a control effect only lower than that in group 2 in the first four surveys. Based on Jin’s method, the additive effects between *Datura* extract and Infinito (0.15 mL) for the second and third investigations (Q = 1.06, 1.03, respectively) and synergism effects between them in the fourth and fifth surveys (Q = 1.21, 1.68, respectively) are obtained. In the last survey, the disease indexes of group 3 (47.25%) and group 2 (49.99%) are almost similar, suggesting *Datura* extract and Infinito (1.5 mL) have similar control effects at this time. Due to the continuous rainfall, the 15-d interval exists between the fourth survey and the fifth survey, which leads to the fact that the difference of disease index in each group in the fifth survey is not as obvious as that in the fourth survey (Figure 5C).

The highest average yield is obtained in group 2 (28.52 kg), followed by group 5 (26.84 kg) and group 3 (25.43 kg) (Figure 5D). Compared with the control group (group 1), group 3 elevates production by 22.38%, with a commercial potato yield of 50.96%, and the combination of *Datura* extract and 0.15 mL Infinito (group 4) improves production by 29.16%, with a commercial potato yield of 59.84% (Supplementary Table S9). These results preliminarily imply that the use of *Datura* extract only or in combination with chemical fungicide expands the yield and commercial yield of potatoes, enhancing economic value.

## 3. Discussion

This study uncovers that scopolamine can induce potato late blight resistance by affecting the activities or contents of disease-resistant substances (PPO, POD, H_2_O_2_, etc.) and regulating the DEGs involved in plant–pathogen interaction, cell walls, and membranes. Previous studies found scopolamine could inhibit mycelial growth, sporangium germination and viability, and the pathogenicity of *P. infestans* [14]. It can be seen that scopolamine had dual functions of anti-oomycete and induced the disease resistance of potato, which is similar to chitosan against potato late blight [31], and putrescine against mango anthracnose [32]. These results suggest many bioactive materials have simultaneous bacteriostasis and induced disease resistance. While there are also some exceptions, for example, melatonin has no significant effect on spore germination and the growth of *Penicillium digitatum*, but it can reduce the resistance of citrus fruits to pathogens [33]. The dual functions of anti-oomycete and disease-inducing resistance of scopolamine make it an environmentally friendly agent for potato late blight controlling. However, scopolamine is particularly expensive ($3.08/g), so we turned our attention to *Datura* extract (USD 0.03/g), in which the main bioactive substance is scopolamine. It also shows dual functions of anti-oomycete and reduced disease resistance in vitro. In the field experiment, both Infinito (0.15 mL, 10% of the field recommended amount) and *Datura* extract alone are poorly effective in preventing foliar late blight. While, the combination of *Datura* extract and Infinito (0.15 mL) shows a synergistic effect in the fourth survey, indicating that *Datura* extract has the potential to reduce the use of the chemical fungicide Infinito. In addition, the last survey of disease index found that pure *Datura* extract has the best control effect, while Infinito (1.5 mL) has the worst control effect. A possible reason is that the fungicide Infinito and *Datura* extract are not used in time during 15 d of continuous rain, and the lasting ability of *Datura* extract is greater than that of Infinito. Further multi-year field experiments are needed to confirm the conjecture. Noteworthy is that scopolamine can induce potato to resist the infection of *P. infestans* in vitro and in vivo, and it is different in different potato varieties. The inducing effect of scopolamine on ‘Qingshu 9’ and ‘Marco’ is better than that on ‘Favorite’ and ‘Xisen 6’. Therefore, cultivars need to be concerned when specifically utilizing the induced disease resistance of scopolamine against late blight.

ROS burst leads to cell necrosis at the infected site to prevent the spread of pathogens [34]. H_2_O_2_ is the most important ROS, which can act as a signal molecule to induce downstream disease resistance [35]. During the interaction between potato and *P. infestans*, scopolamine can induce higher H_2_O_2_ content in potato tuber at 2nd h after scopolamine treatment than that in control group, suggesting that the effect of scopolamine on potato disease resistance is related to ROS. Meanwhile, the exogenous H_2_O_2_ may help potato resist *P. infestans*, further confirming the role of H_2_O_2_ in potato late blight resistance. ROS is controlled by a series of enzymes such as SOD, which is associated with defense, and CAT, which promotes the breakdown of H_2_O_2_. These enzymes are generally considered to be key enzymes in host defense against pathogenic infections. From 2nd h to 5th d after scopolamine treatment, SOD activity is upregulated and CAT activity downregulated, contributing to the development of late blight resistance, which is similar to the results of previous studies using epsilon-poly-l-lysine to induce *Pseudomonas tolaasii* disease resistance in *Agaricus bisporus* [36]. In addition, increased PPO activity can promote the production of quinones, thereby limiting the proliferation and spread of *P. infestans* [37]. PAL is involved in salicylic acid biosynthesis, which is an important signal of systemic resistance in plants [38]. PAL activity increases to fight pathogens at the onset of plant defense and then decreases rapidly [39]. Our control group results are consistent with this, indicating that *P. infestans* successfully infected potato tubers. Compared with control group, scopolamine treatment inhibited PAL activity. PAL can be directly involved in lignin synthesis associated with cell wall components. POD activity can promote the cross-linking between proteins and phenylpropionic acid free radicals, thereby enhancing the resistance of cell wall to fungal invasion [40]. These phenomena were also detected in experiments in which *β*-amino-butyric acid induced resistance to *Rhizoctonia solani* Kühn in rice [9], suggesting that the resistance mechanism of the two drugs to pathogens may be similar. Also, the upregulation of nine DEGs associated with pectinase is detected in the transcriptome data associated with the cell wall (Supplementary Table S7), these evidences seem to support that scopolamine treatment promotes plant cell wall consolidation and, subsequently, disease resistance. Scopolamine may also have an effect on the ETI pathway. Scopolamine increases the expression of auxin-responsive GH3 family proteins and UDP-glycosyltransferase genes in potato. Auxin-responsive GH3 family proteins and UDP-glycosyltransferase genes are involved in plant growth and development and biological stress responses in ETI [41]. Auxin not only regulates plant growth and development, but also regulates membrane function and gene expression, enabling plants to respond to stress [42]. UDP-glycosyltransferase is an important glycosylation catalyst in plants, which can activate plant hormones and defense compounds under stress [43]. The higher number of up-regulated DEGs in the ETI pathway led us to speculate that scopolamine may improve plant response to biotic stress and promote plant growth and development by increasing the expression of regulatory hormones and cell membrane-related genes in ETI.

## 4. Materials and Methods

### 4.1. Materials

Scopolamine, 98% pure, was purchased from Hongyu Medical Co., Ltd., Hangzhou, China. Scopolamine was dissolved in sterile water. *P. infestans* strain 88,069 was provided by Professor Jiasui Zhan at Fujian Agriculture and Forestry University, China. The strain was inoculated on rye glucose agar medium [44] and incubated at 20 °C in the dark. Potatoes ‘Favorita’, ‘Marco’, ‘Qingshu 9’, ‘Xisen 6’, and ‘Hongmei’ were planted and harvested in Chongqing University, China. Green leaves of consistent size and healthy tubers were selected for induction experiments. *Datura* extract, $0.03/g, produced by Xiazhou Biotechnology Co., Ltd., Shaanxi, China, was made from *Datura metel* L. Infinito was diluted with water, manufactured by Bayer AG, Leverkusen, Germany, contains 62.5 g/L fluopicolide and 625 g/L propamocarb hydrochloride.

### 4.2. The Effect of Scopolamine on Inducing the Resistance of Potato Pieces, Leaves, and Whole Potatoes to Late Blight

Late blight resistance induction in potato pieces (3 cm × 4 cm × 0.6 cm) and leaves (in vitro experiment) was performed according to the method of Liu et al. [45]. The potato pieces and leaves were washed with water, the surface sterilized with 75% alcohol for 30 s, dried, then divided into six groups, immersed in water (control group), 1.5, 3.0, 4.5, 6.0 and 7.5 g/L scopolamine solution for 1 h. The pieces and leaves were washed again with clean water and allowed to dry. For the whole potato experiment (in vivo experiment), control group and 4.5 g/L scopolamine solution treatment group were set up, and the soaking treatment was performed similarly to the pieces and leaves. Each group was repeated three times. After that, the leaves, pieces, and whole potatoes were inoculated with *P. infestans* mycelial disks (6-mm-diameter), and the potato pieces and whole potatoes were placed in a dark environment, while the 12 h white light/12 h dark cycle was given to the leaves. All of them were cultured in a closed environment at room temperature. After 7 d of cultivation of potato pieces and leaves, and 8, 12, 16, 20 d for whole potatoes, photo recordings were made. The proportion of lesion area (lesion area divided by total area) were calculated by ImageJ software. To confirm the widespread resistance induction of scopolamine in different varieties of potato, ‘Favorita’ was used for potato pieces and leaves experiment and ‘Marco’ was used for the whole potato experiment. The method of ‘Qingshu 9’, ‘Xisen 6’, and ‘Hongmei’ was the same as that of ‘Favorita’ potato pieces experiment, but only control group and 4.5 g/L scopolamine treatment group were set up.

### 4.3. Assays of Disease-Resistant Substances in Potato Tubers

‘Favorita’ potato pieces were treated with or without scopolamine treatment (IC50) for 1 h. The pieces were rinsed, air dried and inoculated with *P. infestans.* On the 2nd h, 3rd, 5th and 8th d after treated with *P. infestans*, the 1 cm^3^ potato tissue around the inoculation site was obtained for extracting enzymes. Determining the activity of PAL, PPO, POD, SOD, CAT, and the content of H_2_O_2_. PAL, PPO, CAT test kits (Beijing Leagene Biotech. Co., Ltd.) and POD, SOD, H_2_O_2_ test kits (Grace Biotechnology Co., Ltd, Suzhou, China.) were used to extract corresponding enzymes and their enzyme activities were calculated based on absorbance changes. Absorbance was measured with multi-mode reader (SYNERGY HTX, BioTek Instruments, Inc, Winooski, VT, USA.).

### 4.4. The Effect of H_2_O_2_ on Inducing the Resistance of Potato Pieces

H_2_O_2_ is not only an important signal molecule in plants after infection by pathogens, but also known as an antibacterial material. Scopolamine may induce the regulation of H_2_O_2_ content in potato tubers, which makes potato resistant to disease in both signal transduction and direct inhibition. To explore whether H_2_O_2_ could induce resistance to *P. infestans* in potato tuber, ‘Favorita’ potato pieces (3 cm × 4 cm × 0.6 cm) were selected and immersed in H_2_O_2_ (200 μM) or sterile water (control) for 1 h. Other procedures were identical to those described in Section 4.2. Pictures were taken and recorded after 5 d of culture.

### 4.5. RNA Sequencing

The clean, sterilized ‘Favorita’ potato pieces were soaked in scopolamine solution (IC50) and distilled water (Control) for 1 h, and then they were washed and dried. *P. infestans* was inoculated in the center of pieces and cultured for 5 d as procedure in Section 4.2. The mycelia were removed from the tubers, and the tissues (2 cm in diameter and 2 cm in thickness) around the inoculation point were taken off. The tissues were soaked in liquid nitrogen for 20 min and then stored in a −80 °C refrigerator for further transcriptome sequence in Majorbio Co., Ltd., Shanghai, China. Each treatment was repeated three times. Plant RNA Purification Reagent (Thermo Fisher Technology Co., Ltd., Shanghai, China) was used to extract total RNA, and gDNA was removed by TaKaRa DNase I. The RNA-seq library was built using the Illumina Truseq^TM^ RNA sample prep Kit method based on the Illumina Novaseq 6000 sequencing platform (Illumina, Inc, San Diego, CA, USA). The software DESeq2 (with biological repetition) was used for differential expression analysis according to the read count data compared to genes with the negative binomial distribution model. Differentially expressed genes (DEGs) with |log_2_FC| ≥ 1 and *p*-adjustment ≤ 0.05 were considered to be significant. The software Goatools (version 0.6.5, https://github.com/tanghaibao/GOatools, accessed on 18 November 2024) was used to perform GO enrichment analysis of DEGs [46]. KEGG pathway enrichment analysis of DEGs was conducted by the software KOBAS (version 2.1.1, http://kobas.cbi.pku.edu.cn/download.php, accessed on 18 November 2024) [47].

### 4.6. Quantitative Real-Time PCR Analysis

The total RNA was extracted with the same sample as Section 4.5 according to PrimeScript™RT reagent Kit (TakaRa Biomedical Technology Co., Ltd., Beijing, China). TaKaRa TBGreen^®^ PremixExTaq™II (RR820A) was used to amplify the target genes in Bio-RadCFX96 System. The 3 most significant upregulated (Soltu.DM.05G015480, Soltu.DM.12G023080, Soltu.DM.09G029520) and the 3 most significant downregulated DEGs (Soltu.DM.08G029740, Soltu.DM.10G021890, Soltu.DM.07G024560) were chosen for qRT-PCR. Primer Premier 6 was used to design the primers (Supplementary Table S1). The standard procedure of two-step PCR amplification was adopted, step 1: 95 °C for 30 s; step 2: PCR reaction; GOTO: 39 (40 Cycles), 95 °C for 5 s and 60 °C for 30 s; step 3: melt curve.

### 4.7. Effect of Datura Extract on Mycelia Growth of P. infestans

The 6-mm-diameter mycelia disks were inoculated in the center of the solid rye glucose medium added with the concentrations of 0, 1.5, 3.0, 4.5, 6.0, 7.5 and 9.0 g/L *Datura* extract. Each concentration group had four replicates, and the mediums were cultured at 20 °C of darkness. After 10 d, photos were taken to record the colony morphology. The colony diameter was determined by cross method, and the inhibition ratio was calculated by the following formula:

(1) $$Inhibition\;ratio\;(\%)=\frac{\left(Control\;group\;colony\;diameter-Datura\;extract\;treated\;colony\;diameter\right)}{\left(Control\;group\;colony\;diameter-initial\;colony\;diameter\right)}\times 100\%$$

### 4.8. Effect of Datura Extract on Induction of Late Blight Resistance in Potato Pieces

The protocol to explore the induced resistance of *Datura* extract to ‘Favorita’ potato pieces was referred to in 4.2. Potato pieces were soaked with different concentrations (0, 1.5, 3.0, 4.5, 6.0, 7.5 and 9.0 g/L) of *Datura* extract solution and rinsed with water. Each group was repeated three times. *P. infestans* was inoculated and the potato pieces were photographed after 7 d of culture, and the proportion of lesion area was calculated using imageJ software (version 1.53k).

### 4.9. Field Test

Potatoes (‘Favorita’, bought from Nongdeli Biological Co., Ltd, Jinan, China.) were grown at the Huxi campus of Chongqing University, China (altitude 286 m, longitude 29.60° E, latitude 106.31° N, red soil with moderate fertility). Potatoes were sowed on 17 January 2023, with 1500 kg of compound fertilizer (N-P-K: 19-5-21, Sinochem Fertilizer Co., Ltd, Beijing, China.) per hectare, and were harvested on 7 May 2023. A total of 15 plots (2.8 m × 4 m) were set up for 5 groups of treatments, and 3 parallel treatments were assigned to each group randomly (Supplementary Figure S1A). Each plot had the same cultivation conditions, ridged and covered with plastic film (single ridge single row). There were a total of 40 plants in each plot, 10 plants per row. On 15 March 2023, the first case of late blight was discovered. The following treatments were sprayed about every 7 d: (1) Control (water); (2) Infinito (1.5 mL); (3) *Datura* extract (40 g); (4) Infinito (0.15 mL); (5) *Datura* extract (40 g) + Infinito (0.15 mL). The spraying time was adjusted according to precipitation and temperature (Supplementary Figure S1B) and the disease index (DI) of the potato late blight in plots were shown in Supplementary Table S2.

Starting with the second spraying, the occurrence of late blight in potato leaves was investigated according to the 0–5 classification standard of potato late blight per plant before every spraying (Supplementary Table S3). The five surveys were given on 2, 6, 13, 19 April and 5 May 2023. The disease index and control effect (CE) were calculated by following formulas.

(2) $$DI(\%)=\frac{\sum_{s=1}^{S}s\cdot n_{s}}{S\cdot N}\times 100\%$$

*DI*, *s*, *n_s_*, *S* and *N* represent the disease index, each disease level, the number of plants at each disease level, the highest disease level and the total number of plants, respectively, and $N=\sum_{s=1}^{S}n_{s}$.

(3) $$CE(\%)=\frac{DI_{c}-DI_{t}}{DI_{C}}\times 100\%$$

Defining *CE* (%) as the control effect, where *DI_c_* and *DI_t_* are the disease indices of the control group and treatment group, respectively.

Jin’s method [48] was used to evaluate the combined use of *Datura* extract and Infinito. ED, EI, and E (D + I) were the inhibition rate of *Datura* extract, Infinito and the combined inhibition rate of the two drugs, respectively. The Q value was calculated according to the following formula.

(4) $$Q=\frac{E(D+I)}{\left(ED+EI-ED\times EI\right)}$$

Q values  < 0.85, 0.85–1.15 and ≥1.15 indicate antagonism, additive effects, and synergism, respectively. All treatments required three repetitions. The harvested healthy potatoes were divided into non-commodity potatoes and commodity potatoes (≥100 g), and the commodity potato rate and yield increase rate were calculated.

(5) $$R_{com}(\%)=\frac{m_{com}}{M}\times 100\%$$

*R_com_* (%) represents the commodity potato rate; *m_com_* and *M* denote the commodity potato weight and total potato weight, respectively.

(6) $$R_{y}(\%)=\frac{m_{t}-m_{c}}{m_{c}}\times 100\%$$

*R_y_* (%) represents the yield increase rate; *m_t_* and *m_c_* represent the weight of the treatment group and control group, respectively.

### 4.10. Statistical Analysis

Excel software was used for the simple calculation of all the original data. Dunnett’s multiple comparisons test and Tukey’s multiple comparisons test were used to analyze the significance of data in GraphPad software (version 8.0.2).

## 5. Conclusions

This study examined the ability of scopolamine to induce potato resistance to late blight caused by *P. infestans*. In vitro and in vivo experiments were conducted to find that scopolamine could be able to enhance the resistance of potatoes to *P. infestans*. This effect was also present in several kinds of potato varieties (‘Favorita’, ‘Marco’, ‘Qingshu 9’, ‘Xisen 6’, and ‘Hongmei’), and tissues (tuber and leaf). The upregulation of PPO, POD, SOD activities and H_2_O_2_ content, and the downregulation of PAL and CAT activities, are the preliminary biochemical responses of potato late blight resistance induced by scopolamine. Transcriptome sequencing results support the view that scopolamine may affect gene functions in cell walls, cell membranes, and plant–pathogen interactions. *Datura* extract shows a dual effect of directly inhibiting mycelial growth and inducing potato disease resistance in vitro. Also, it could be used in combination with Infinito, a chemical fungicide, to reduce late blight symptoms and increase potato yield in the field. To the best of our knowledge, this is the first study to examine scopolamine-induced disease resistance, providing an exploration for the use of *Datura* extract in potato production. However, the detection time point of resistant substances is not enough to reflect the whole process of tuber infection by *P. infestans*, and the molecular study is only limited to the transcriptional level. In addition, the field experiment in this study lasted for only one year, and the results obtained are only preliminary. In the future, multiyear and multisite field experiments need to be conducted to determine the optimal ratio and dosage of Infinito in combination with *Datura* extract. Also, more experiments on post-transcriptional level and translational regulation should be carried out to further explore the molecular mechanism of scopolamine on potato late blight.

## Data Availability

The datasets generated during and/or analysed during the current study are available from the corresponding author on reasonable request.

## Supplementary Materials

The following supporting information can be downloaded at: https://www.mdpi.com/article/10.3390/ijms252413442/s1.
