# Supplementary material for: The Induction of Disease Resistance by Scopolamine and the Application of Datura Extract Against Potato (Solanum tuberosum L.) Late Blight
Source: Int J Mol Sci. 2024 Dec 15;25(24):13442. doi: 10.3390/ijms252413442 (PMC11676833; doi:10.3390/ijms252413442)
Supplement: Supplementary file 1 [file ijms-25-13442-s001.zip › Supplementary Table 1.docx]

**Supplementary Table 1 Primers designed for qRT-PCR**

| gene name | sense primer (5’-3’) | antisense primer (3’-5’) |
| --- | --- | --- |
| Soltu.DM.05G015480 | TCCTCCGCAACCTGGCTCAAT | AACTGGCAGCAGCAACCATCTT |
| Soltu.DM.12G023080 | TCGCGGCGATATTTCAAGCAAT | TCCACCAACAACTCTTCCACCT |
| Soltu.DM.09G029520 | ACTTCTTTCACACCGAGCACAA | GCACATAGCGTAGGCAACTCAT |
| Soltu.DM.08G029740 | CGTTGTCTTGTCGGTCCTCTGT | GCACCATCGCCTTCTTCCTCTT |
| Soltu.DM.10G021890 | AGCAAACAAACAGGTGCAAAGA | ACAGTTTCCACCATCATACCCA |
| Soltu.DM.07G024560 | TTGCGATGTGAAACCCTCTGAG | CGAAATCAAGTCCAACGGTGTG |
