# Supplementary material for: The Induction of Disease Resistance by Scopolamine and the Application of Datura Extract Against Potato (Solanum tuberosum L.) Late Blight
Source: Int J Mol Sci. 2024 Dec 15;25(24):13442. doi: 10.3390/ijms252413442 (PMC11676833; doi:10.3390/ijms252413442)
Supplement: Supplementary file 1 [file ijms-25-13442-s001.zip › Supplementary Table 3.docx]

**Supplementary Table 3 Classification standard for single plant of potato late blight**

| disease grade | state of an illness |
| --- | --- |
| 0 | no disease |
| 1 | there are fewer lesions, only 5-10 lesions per plant |
| 2 | the incidence of leaf lesions was less than 25% |
| 3 | the disease was moderate and 50% of the leaves were infected |
| 4 | the plants were heavily defoliated, but not dead |
| 5 | plant dead |
