# Supplementary material for: The Induction of Disease Resistance by Scopolamine and the Application of Datura Extract Against Potato (Solanum tuberosum L.) Late Blight
Source: Int J Mol Sci. 2024 Dec 15;25(24):13442. doi: 10.3390/ijms252413442 (PMC11676833; doi:10.3390/ijms252413442)
Supplement: Supplementary file 1 [file ijms-25-13442-s001.zip › Supplementary Table 5.docx]

**Supplementary Table 5 The total GO terms in transcriptome data**

| GO ID | term type | description | *P* value | number |
| --- | --- | --- | --- | --- |
| GO:0009595 | BP | detection of biotic stimulus | 1.94E-08 | 7 |
| GO:0098581 | BP | detection of external biotic stimulus | 1.94E-08 | 7 |
| GO:0002221 | BP | pattern recognition receptor signaling pathway | 6.07E-08 | 4 |
| GO:0098543 | BP | detection of other organism | 6.07E-08 | 4 |
| GO:0016045 | BP | detection of bacterium | 6.07E-08 | 4 |
| GO:0018105 | BP | peptidyl-serine phosphorylation | 1.25E-07 | 31 |
| GO:0018209 | BP | peptidyl-serine modification | 1.58E-07 | 31 |
| GO:0042391 | BP | regulation of membrane potential | 1.76E-07 | 15 |
| GO:0050832 | BP | defense response to fungus | 1.90E-07 | 12 |
| GO:0050664 | MF | oxidoreductase activity, acting on NAD(P)H, oxygen as acceptor | 1.58E-07 | 10 |
| GO:0022832 | MF | voltage-gated channel activity | 1.90E-07 | 17 |
| GO:0005244 | MF | voltage-gated ion channel activity | 1.90E-07 | 17 |
| GO:0022836 | MF | gated channel activity | 1.94E-07 | 17 |
| GO:0009755 | BP | hormone-mediated signaling pathway | 3.06E-07 | 32 |
| GO:0044419 | BP | interspecies interaction between organisms | 3.25E-07 | 23 |
| GO:0015079 | MF | potassium ion transmembrane transporter activity | 2.79E-07 | 18 |
| GO:0010857 | MF | calcium-dependent protein kinase activity | 3.18E-07 | 31 |
| GO:0009931 | MF | calcium-dependent protein serine/threonine kinase activity | 3.18E-07 | 31 |
| GO:0009620 | BP | response to fungus | 4.10E-07 | 12 |
| GO:0009738 | BP | abscisic acid-activated signaling pathway | 4.60E-07 | 30 |
| GO:0005887 | CC | integral component of plasma membrane | 4.25E-07 | 15 |
| GO:0005249 | MF | voltage-gated potassium channel activity | 4.60E-07 | 17 |
| GO:0018193 | BP | peptidyl-amino acid modification | 5.01E-07 | 31 |
| GO:0046777 | BP | protein autophosphorylation | 5.87E-07 | 35 |
| GO:0016310 | BP | phosphorylation | 6.21E-07 | 49 |
| GO:0051707 | BP | response to other organism | 7.72E-07 | 23 |
| GO:0009605 | BP | response to external stimulus | 8.06E-07 | 23 |
| GO:0052542 | BP | defense response by callose deposition | 8.88E-07 | 4 |
| GO:0052544 | BP | defense response by callose deposition in cell wall | 8.88E-07 | 4 |
| GO:0052482 | BP | defense response by cell wall thickening | 8.88E-07 | 4 |
| GO:0009607 | BP | response to biotic stimulus | 9.21E-07 | 31 |
| GO:0098542 | BP | defense response to other organism | 9.50E-07 | 23 |
| GO:0006952 | BP | defense response | 9.61E-07 | 38 |
| GO:0004683 | MF | calmodulin-dependent protein kinase activity | 6.52E-07 | 31 |
| GO:0005261 | MF | cation channel activity | 6.60E-07 | 17 |
| GO:0046873 | MF | metal ion transmembrane transporter activity | 6.87E-07 | 18 |
| GO:0051082 | MF | unfolded protein binding | 8.62E-07 | 11 |
| GO:0015267 | MF | channel activity | 8.93E-07 | 21 |
| GO:0022803 | MF | passive transmembrane transporter activity | 8.93E-07 | 21 |
| GO:0022843 | MF | voltage-gated cation channel activity | 9.10E-07 | 17 |
| GO:0005216 | MF | ion channel activity | 9.28E-07 | 21 |
| GO:0005516 | MF | calmodulin binding | 9.48E-07 | 31 |
| GO:0051606 | BP | detection of stimulus | 1.05E-06 | 7 |
| GO:0006468 | BP | protein phosphorylation | 1.07E-06 | 49 |
| GO:0005267 | MF | potassium channel activity | 1.07E-06 | 17 |
| GO:0043207 | BP | response to external biotic stimulus | 1.15E-06 | 23 |
| GO:0005509 | MF | calcium ion binding | 1.18E-06 | 107 |
| GO:0035556 | BP | intracellular signal transduction | 1.26E-06 | 43 |
| GO:0045087 | BP | innate immune response | 1.42E-06 | 10 |
| GO:0007165 | BP | signal transduction | 1.48E-06 | 59 |
| GO:0042742 | BP | defense response to bacterium | 1.53E-06 | 9 |
| GO:0006796 | BP | phosphate-containing compound metabolic process | 1.58E-06 | 51 |
| GO:0006793 | BP | phosphorus metabolic process | 1.88E-06 | 51 |
| GO:0006955 | BP | immune response | 1.93E-06 | 10 |
| GO:0006464 | BP | cellular protein modification process | 2.24E-06 | 49 |
| GO:0036211 | BP | protein modification process | 2.24E-06 | 49 |
| GO:0004674 | MF | protein serine/threonine kinase activity | 1.44E-06 | 161 |
| GO:0015077 | MF | monovalent inorganic cation transmembrane transporter activity | 1.88E-06 | 18 |
| GO:0004672 | MF | protein kinase activity | 1.89E-06 | 185 |
| GO:0043531 | MF | ADP binding | 2.07E-06 | 56 |
| GO:0035639 | MF | purine ribonucleoside triphosphate binding | 2.10E-06 | 196 |
| GO:0005524 | MF | ATP binding | 2.26E-06 | 195 |
| GO:0016301 | MF | kinase activity | 2.26E-06 | 203 |
| GO:0140096 | MF | catalytic activity, acting on a protein | 2.36E-06 | 190 |
| GO:0006950 | BP | response to stress | 2.47E-06 | 59 |
| GO:0016772 | MF | transferase activity, transferring phosphorus-containing groups | 2.47E-06 | 203 |
| GO:0016773 | MF | phosphotransferase activity, alcohol group as acceptor | 2.63E-06 | 186 |
| GO:0046872 | MF | metal ion binding | 2.78E-06 | 109 |
| GO:0043169 | MF | cation binding | 2.85E-06 | 112 |
| GO:1901265 | MF | nucleoside phosphate binding | 2.86E-06 | 251 |
| GO:0000166 | MF | nucleotide binding | 2.86E-06 | 251 |
| GO:0016740 | MF | transferase activity | 3.05E-06 | 206 |
| GO:0097367 | MF | carbohydrate derivative binding | 3.06E-06 | 251 |
| GO:0043168 | MF | anion binding | 3.08E-06 | 251 |
| GO:0030554 | MF | adenyl nucleotide binding | 3.09E-06 | 250 |
| GO:0032559 | MF | adenyl ribonucleotide binding | 3.10E-06 | 250 |
| GO:0005886 | CC | plasma membrane | 3.16E-06 | 46 |
| GO:0032555 | MF | purine ribonucleotide binding | 3.32E-06 | 251 |
| GO:0017076 | MF | purine nucleotide binding | 3.39E-06 | 251 |
| GO:0036094 | MF | small molecule binding | 3.40E-06 | 251 |
| GO:0097159 | MF | organic cyclic compound binding | 3.43E-06 | 273 |
| GO:0009617 | BP | response to bacterium | 3.83E-06 | 9 |
| GO:0002429 | BP | immune response-activating cell surface receptor signaling pathway | 3.88E-06 | 3 |
| GO:0002768 | BP | immune response-regulating cell surface receptor signaling pathway | 3.88E-06 | 3 |
| GO:0002220 | BP | innate immune response activating cell surface receptor signaling pathway | 3.88E-06 | 3 |
| GO:0002752 | BP | cell surface pattern recognition receptor signaling pathway | 3.88E-06 | 3 |
| GO:0032499 | BP | detection of peptidoglycan | 3.88E-06 | 3 |
| GO:0032494 | BP | response to peptidoglycan | 3.88E-06 | 3 |
| GO:0032490 | BP | detection of molecule of bacterial origin | 3.88E-06 | 3 |
| GO:0032491 | BP | detection of molecule of fungal origin | 3.88E-06 | 3 |
| GO:0031224 | CC | intrinsic component of membrane | 3.58E-06 | 216 |
| GO:0032553 | MF | ribonucleotide binding | 3.69E-06 | 251 |
| GO:0043167 | MF | ion binding | 3.78E-06 | 321 |
| GO:2001080 | MF | chitosan binding | 3.88E-06 | 3 |
| GO:0052543 | BP | callose deposition in cell wall | 4.04E-06 | 4 |
| GO:0016021 | CC | integral component of membrane | 4.44E-06 | 216 |
| GO:1901363 | MF | heterocyclic compound binding | 4.42E-06 | 273 |
| GO:0003674 | MF | molecular_function | 4.54E-06 | 389 |
| GO:0005488 | MF | binding | 4.66E-06 | 343 |
| GO:0004675 | MF | transmembrane receptor protein serine/threonine kinase activity | 5.23E-06 | 12 |
| GO:0019199 | MF | transmembrane receptor protein kinase activity | 6.40E-06 | 12 |
| GO:0032412 | BP | regulation of ion transmembrane transporter activity | 7.19E-06 | 4 |
| GO:0052386 | BP | cell wall thickening | 7.19E-06 | 4 |
| GO:0022898 | BP | regulation of transmembrane transporter activity | 7.19E-06 | 4 |
| GO:0032409 | BP | regulation of transporter activity | 7.19E-06 | 4 |
| GO:0052545 | BP | callose localization | 7.19E-06 | 4 |
| GO:0010359 | BP | regulation of anion channel activity | 7.19E-06 | 4 |
| GO:0033037 | BP | polysaccharide localization | 7.19E-06 | 4 |
| GO:0002376 | BP | immune system process | 9.78E-06 | 10 |
| GO:1903959 | BP | regulation of anion transmembrane transport | 1.18E-05 | 4 |
| GO:0071323 | BP | cellular response to chitin | 1.53E-05 | 3 |
| GO:0002238 | BP | response to molecule of fungal origin | 1.53E-05 | 3 |
| GO:0004198 | MF | calcium-dependent cysteine-type endopeptidase activity | 1.53E-05 | 3 |
| GO:0044070 | BP | regulation of anion transport | 1.84E-05 | 4 |
| GO:0043412 | BP | macromolecule modification | 2.14E-05 | 49 |
| GO:0010200 | BP | response to chitin | 2.52E-05 | 5 |
| GO:0031226 | CC | intrinsic component of plasma membrane | 2.60E-05 | 15 |
| GO:0071219 | BP | cellular response to molecule of bacterial origin | 3.79E-05 | 3 |
| GO:0006898 | BP | receptor-mediated endocytosis | 5.36E-05 | 4 |
| GO:0004888 | MF | transmembrane signaling receptor activity | 5.78E-05 | 12 |
| GO:0007166 | BP | cell surface receptor signaling pathway | 6.38E-05 | 12 |
| GO:0044267 | BP | cellular protein metabolic process | 6.53E-05 | 50 |
| GO:0071417 | BP | cellular response to organonitrogen compound | 7.49E-05 | 3 |
| GO:0071216 | BP | cellular response to biotic stimulus | 7.49E-05 | 3 |
| GO:0110165 | CC | cellular anatomical entity | 0.000105719 | 319 |
| GO:0009593 | BP | detection of chemical stimulus | 0.000129592 | 3 |
| GO:0043269 | BP | regulation of ion transport | 0.000130445 | 6 |
| GO:0006457 | BP | protein folding | 0.000189779 | 11 |
| GO:0038023 | MF | signaling receptor activity | 0.000200355 | 12 |
| GO:0002764 | BP | immune response-regulating signaling pathway | 0.000204922 | 3 |
| GO:0002757 | BP | immune response-activating signal transduction | 0.000204922 | 3 |
| GO:0002758 | BP | innate immune response-activating signal transduction | 0.000204922 | 3 |
| GO:0009626 | BP | plant-type hypersensitive response | 0.00022273 | 6 |
| GO:0034050 | BP | host programmed cell death induced by symbiont | 0.00022273 | 6 |
| GO:0022890 | MF | inorganic cation transmembrane transporter activity | 0.000237894 | 18 |
| GO:0060089 | MF | molecular transducer activity | 0.000246611 | 12 |
| GO:0016174 | MF | NAD(P)H oxidase (H(2)O(2)-forming activity | 0.000247544 | 2 |
| GO:0009817 | BP | defense response to fungus, incompatible interaction | 0.000303791 | 3 |
| GO:0012501 | BP | programmed cell death | 0.000394076 | 6 |
| GO:0002237 | BP | response to molecule of bacterial origin | 0.000428918 | 3 |
| GO:0009814 | BP | defense response, incompatible interaction | 0.000502449 | 4 |
| GO:0008324 | MF | cation transmembrane transporter activity | 0.000521401 | 18 |
| GO:0016651 | MF | oxidoreductase activity, acting on NAD(P)H | 0.000527704 | 10 |
| GO:0034765 | BP | regulation of ion transmembrane transport | 0.000557182 | 5 |
| GO:0005515 | MF | protein binding | 0.000561486 | 50 |
| GO:0005576 | CC | extracellular region | 0.000598775 | 22 |
| GO:0034762 | BP | regulation of transmembrane transport | 0.000620571 | 5 |
| GO:0008219 | BP | cell death | 0.000654359 | 6 |
| GO:0031098 | BP | stress-activated protein kinase signaling cascade | 0.000959153 | 6 |
| GO:0023014 | BP | signal transduction by protein phosphorylation | 0.000959153 | 6 |
| GO:0006897 | BP | endocytosis | 0.001055429 | 4 |
| GO:0050896 | BP | response to stimulus | 0.001421256 | 60 |
| GO:0005262 | MF | calcium channel activity | 0.001454393 | 2 |
| GO:1901699 | BP | cellular response to nitrogen compound | 0.001533752 | 3 |
| GO:0032147 | BP | activation of protein kinase activity | 0.001660606 | 6 |
| GO:0065009 | BP | regulation of molecular function | 0.001995718 | 10 |
| GO:0004713 | MF | protein tyrosine kinase activity | 0.002733163 | 5 |
| GO:0004601 | MF | peroxidase activity | 0.00279112 | 10 |
| GO:0015075 | MF | ion transmembrane transporter activity | 0.002799705 | 22 |
| GO:0015318 | MF | inorganic molecular entity transmembrane transporter activity | 0.002911755 | 22 |
| GO:0016684 | MF | oxidoreductase activity, acting on peroxide as acceptor | 0.00297704 | 10 |
| GO:0002218 | BP | activation of innate immune response | 0.003117318 | 3 |
| GO:0002253 | BP | activation of immune response | 0.003117318 | 3 |
| GO:0045860 | BP | positive regulation of protein kinase activity | 0.003552549 | 6 |
| GO:0001934 | BP | positive regulation of protein phosphorylation | 0.003745483 | 6 |
| GO:0033674 | BP | positive regulation of kinase activity | 0.003946079 | 6 |
| GO:0010243 | BP | response to organonitrogen compound | 0.004109602 | 5 |
| GO:0010562 | BP | positive regulation of phosphorus metabolic process | 0.004154519 | 6 |
| GO:0042327 | BP | positive regulation of phosphorylation | 0.004154519 | 6 |
| GO:0045937 | BP | positive regulation of phosphate metabolic process | 0.004154519 | 6 |
| GO:0005737 | CC | cytoplasm | 0.004252152 | 44 |
| GO:0051049 | BP | regulation of transport | 0.005580051 | 6 |
| GO:0002684 | BP | positive regulation of immune system process | 0.006143305 | 3 |
| GO:0050778 | BP | positive regulation of immune response | 0.006143305 | 3 |
| GO:0045089 | BP | positive regulation of innate immune response | 0.006143305 | 3 |
| GO:0051347 | BP | positive regulation of transferase activity | 0.006710287 | 6 |
| GO:0019538 | BP | protein metabolic process | 0.006784376 | 53 |
| GO:0071495 | BP | cellular response to endogenous stimulus | 0.00690047 | 3 |
| GO:0045859 | BP | regulation of protein kinase activity | 0.00766063 | 6 |
| GO:0043549 | BP | regulation of kinase activity | 0.008345764 | 6 |
| GO:0032879 | BP | regulation of localization | 0.008345764 | 6 |
| GO:0031349 | BP | positive regulation of defense response | 0.00857448 | 3 |
| GO:0002833 | BP | positive regulation of response to biotic stimulus | 0.00857448 | 3 |
| GO:0005102 | MF | signaling receptor binding | 0.00857448 | 3 |
| GO:0007346 | BP | regulation of mitotic cell cycle | 0.009073805 | 6 |
| GO:0031401 | BP | positive regulation of protein modification process | 0.009454351 | 6 |
| GO:1901698 | BP | response to nitrogen compound | 0.009594577 | 5 |
| GO:0001932 | BP | regulation of protein phosphorylation | 0.009846147 | 6 |
| GO:0032103 | BP | positive regulation of response to external stimulus | 0.010466423 | 3 |
| GO:0042325 | BP | regulation of phosphorylation | 0.01109073 | 6 |
| GO:0048584 | BP | positive regulation of response to stimulus | 0.011104262 | 5 |
| GO:0042803 | MF | protein homodimerization activity | 0.013983143 | 4 |
| GO:0051338 | BP | regulation of transferase activity | 0.014946263 | 6 |
| GO:0052314 | BP | phytoalexin metabolic process | 0.015751477 | 1 |
| GO:0070509 | BP | calcium ion import | 0.015751477 | 1 |
| GO:0010120 | BP | camalexin biosynthetic process | 0.015751477 | 1 |
| GO:0052315 | BP | phytoalexin biosynthetic process | 0.015751477 | 1 |
| GO:0007263 | BP | nitric oxide mediated signal transduction | 0.015751477 | 1 |
| GO:0009700 | BP | indole phytoalexin biosynthetic process | 0.015751477 | 1 |
| GO:0010157 | BP | response to chlorate | 0.015751477 | 1 |
| GO:0052317 | BP | camalexin metabolic process | 0.015751477 | 1 |
| GO:0046167 | BP | glycerol-3-phosphate biosynthetic process | 0.015751477 | 1 |
| GO:0046217 | BP | indole phytoalexin metabolic process | 0.015751477 | 1 |
| GO:0009403 | BP | toxin biosynthetic process | 0.015751477 | 1 |
| GO:0005217 | MF | intracellular ligand-gated ion channel activity | 0.015751477 | 1 |
| GO:0099094 | MF | ligand-gated cation channel activity | 0.015751477 | 1 |
| GO:0004370 | MF | glycerol kinase activity | 0.015751477 | 1 |
| GO:0043855 | MF | cyclic nucleotide-gated ion channel activity | 0.015751477 | 1 |
| GO:0005221 | MF | intracellular cyclic nucleotide activated cation channel activity | 0.015751477 | 1 |
| GO:0005222 | MF | intracellular cAMP-activated cation channel activity | 0.015751477 | 1 |
| GO:0005242 | MF | inward rectifier potassium channel activity | 0.015751477 | 1 |
| GO:0019220 | BP | regulation of phosphate metabolic process | 0.017185814 | 6 |
| GO:0051174 | BP | regulation of phosphorus metabolic process | 0.017185814 | 6 |
| GO:0051716 | BP | cellular response to stimulus | 0.017771229 | 21 |
| GO:0003968 | MF | RNA-directed 5'-3' RNA polymerase activity | 0.025673615 | 2 |
| GO:0043085 | BP | positive regulation of catalytic activity | 0.026817994 | 6 |
| GO:0005575 | CC | cellular_component | 0.027682168 | 319 |
| GO:0045088 | BP | regulation of innate immune response | 0.028317004 | 3 |
| GO:0009845 | BP | seed germination | 0.028798198 | 2 |
| GO:0016209 | MF | antioxidant activity | 0.029627205 | 10 |
| GO:0050776 | BP | regulation of immune response | 0.030094823 | 3 |
| GO:0032270 | BP | positive regulation of cellular protein metabolic process | 0.030979633 | 6 |
| GO:0010508 | BP | positive regulation of autophagy | 0.03125541 | 1 |
| GO:0009704 | BP | de-etiolation | 0.03125541 | 1 |
| GO:0070370 | BP | cellular heat acclimation | 0.03125541 | 1 |
| GO:0050080 | MF | malonyl-CoA decarboxylase activity | 0.03125541 | 1 |
| GO:0004708 | MF | MAP kinase kinase activity | 0.03125541 | 1 |
| GO:0044093 | BP | positive regulation of molecular function | 0.031859696 | 6 |
| GO:0051247 | BP | positive regulation of protein metabolic process | 0.031859696 | 6 |
| GO:0031399 | BP | regulation of protein modification process | 0.035542079 | 6 |
| GO:0004197 | MF | cysteine-type endopeptidase activity | 0.035772355 | 3 |
| GO:0002682 | BP | regulation of immune system process | 0.037778636 | 3 |
| GO:0071310 | BP | cellular response to organic substance | 0.039841252 | 3 |
| GO:0033554 | BP | cellular response to stress | 0.04015698 | 18 |
| GO:0009408 | BP | response to heat | 0.042224608 | 4 |
| GO:0009873 | BP | ethylene-activated signaling pathway | 0.046465806 | 2 |
| GO:0016324 | CC | apical plasma membrane | 0.04651568 | 1 |
| GO:1901701 | BP | cellular response to oxygen-containing compound | 0.048646979 | 3 |
