# Supplementary material for: The Induction of Disease Resistance by Scopolamine and the Application of Datura Extract Against Potato (Solanum tuberosum L.) Late Blight
Source: Int J Mol Sci. 2024 Dec 15;25(24):13442. doi: 10.3390/ijms252413442 (PMC11676833; doi:10.3390/ijms252413442)
Supplement: Supplementary file 1 [file ijms-25-13442-s001.zip › Supplementary Table 6.docx]

**Supplementary Table 6 The total KEGG pathway analysis in transcriptome data**

| Pathway id | Description | *P* value | First Category | Second Category | *P* value | Number |
| --- | --- | --- | --- | --- | --- | --- |
| map00480 | Glutathione metabolism | 7.99E-08 | Metabolism | Metabolism of other amino acids | 7.99E-08 | 22 |
| map05225 | Hepatocellular carcinoma | 6.70E-07 | Human Diseases | Cancers: Specific types | 6.70E-07 | 21 |
| map04626 | Plant-pathogen interaction | 1.07E-05 | Organismal Systems | Environmental adaptation | 1.07E-05 | 41 |
| map00520 | Amino sugar and nucleotide sugar metabolism | 0.002648794 | Metabolism | Carbohydrate metabolism | 0.002648794 | 17 |
| map00603 | Glycosphingolipid biosynthesis - globo and isoglobo series | 0.006026806 | Metabolism | Glycan biosynthesis and metabolism | 0.006026806 | 3 |
| map00601 | Glycosphingolipid biosynthesis - lacto and neolacto series | 0.00575869 | Metabolism | Glycan biosynthesis and metabolism | 0.00575869 | 2 |
| map00750 | Vitamin B6 metabolism | 0.012710882 | Metabolism | Metabolism of cofactors and vitamins | 0.012710882 | 4 |
| map03060 | Protein export | 0.019747492 | Genetic Information Processing | Folding, sorting and degradation | 0.019747492 | 7 |
| map00360 | Phenylalanine metabolism | 0.028620094 | Metabolism | Amino acid metabolism | 0.028620094 | 9 |
| map04070 | Phosphatidylinositol signaling system | 0.052191979 | Environmental Information Processing | Signal transduction | 0.052191979 | 8 |
| map04625 | C-type lectin receptor signaling pathway | 0.057305456 | Organismal Systems | Immune system | 0.057305456 | 4 |
| map00950 | Isoquinoline alkaloid biosynthesis | 0.049871655 | Metabolism | Biosynthesis of other secondary metabolites | 0.049871655 | 6 |
| map00999 | Biosynthesis of secondary metabolites - unclassified | 0.092405189 | Metabolism | Biosynthesis of other secondary metabolites | 0.092405189 | 7 |
| map00940 | Phenylpropanoid biosynthesis | 0.100516084 | Metabolism | Biosynthesis of other secondary metabolites | 0.100516084 | 22 |
| map00960 | Tropane, piperidine and pyridine alkaloid biosynthesis | 0.114980597 | Metabolism | Biosynthesis of other secondary metabolites | 0.114980597 | 5 |
| map00350 | Tyrosine metabolism | 0.130374536 | Metabolism | Amino acid metabolism | 0.130374536 | 7 |
| map00965 | Betalain biosynthesis | 0.127683415 | Metabolism | Biosynthesis of other secondary metabolites | 0.127683415 | 1 |
| map00052 | Galactose metabolism | 0.145442745 | Metabolism | Carbohydrate metabolism | 0.145442745 | 6 |
| map04979 | Cholesterol metabolism | 0.157573417 | Organismal Systems | Digestive system | 0.157573417 | 2 |
| map00604 | Glycosphingolipid biosynthesis - ganglio series | 0.166518568 | Metabolism | Glycan biosynthesis and metabolism | 0.166518568 | 1 |
| map04130 | SNARE interactions in vesicular transport | 0.181809987 | Genetic Information Processing | Folding, sorting and degradation | 0.181809987 | 4 |
| map03050 | Proteasome | 0.208490173 | Genetic Information Processing | Folding, sorting and degradation | 0.208490173 | 5 |
| map03010 | Ribosome | 0.999998983 | Genetic Information Processing | Translation | 0.999998983 | 3 |
| map05167 | Kaposi sarcoma-associated herpesvirus infection | 0.204592238 | Human Diseases | Infectious diseases: Viral | 0.204592238 | 6 |
| map00562 | Inositol phosphate metabolism | 0.241215924 | Metabolism | Carbohydrate metabolism | 0.241215924 | 5 |
| map00130 | Ubiquinone and other terpenoid-quinone biosynthesis | 0.257648346 | Metabolism | Metabolism of cofactors and vitamins | 0.257648346 | 4 |
| map04075 | Plant hormone signal transduction | 0.272826663 | Environmental Information Processing | Signal transduction | 0.272826663 | 20 |
| map01062 | Biosynthesis of terpenoids and steroids | 0.27296956 | Metabolism | Chemical structure transformation maps | 0.27296956 | 1 |
| map00910 | Nitrogen metabolism | 0.287097448 | Metabolism | Energy metabolism | 0.287097448 | 3 |
| map04016 | MAPK signaling pathway - plant | 0.304708507 | Environmental Information Processing | Signal transduction | 0.304708507 | 19 |
| map05163 | Human cytomegalovirus infection | 0.310083802 | Human Diseases | Infectious diseases: Viral | 0.310083802 | 5 |
| map04144 | Endocytosis | 0.321684023 | Cellular Processes | Transport and catabolism | 0.321684023 | 14 |
| map00905 | Brassinosteroid biosynthesis | 0.32317435 | Metabolism | Metabolism of terpenoids and polyketides | 0.32317435 | 2 |
| map00904 | Diterpenoid biosynthesis | 0.338384185 | Metabolism | Metabolism of terpenoids and polyketides | 0.338384185 | 4 |
| map00333 | Prodigiosin biosynthesis | 0.365843929 | Metabolism | Biosynthesis of other secondary metabolites | 0.365843929 | 1 |
| map00620 | Pyruvate metabolism | 0.421355547 | Metabolism | Carbohydrate metabolism | 0.421355547 | 6 |
| map05226 | Gastric cancer | 0.429953505 | Human Diseases | Cancers: Specific types | 0.429953505 | 4 |
| map00906 | Carotenoid biosynthesis | 0.433787636 | Metabolism | Metabolism of terpenoids and polyketides | 0.433787636 | 3 |
| map00410 | beta-Alanine metabolism | 0.452075122 | Metabolism | Metabolism of other amino acids | 0.452075122 | 5 |
| map02010 | ABC transporters | 0.457387229 | Environmental Information Processing | Membrane transport | 0.457387229 | 3 |
| map00260 | Glycine, serine and threonine metabolism | 0.460806942 | Metabolism | Amino acid metabolism | 0.460806942 | 5 |
| map00400 | Phenylalanine, tyrosine and tryptophan biosynthesis | 0.469030453 | Metabolism | Amino acid metabolism | 0.469030453 | 3 |
| map00040 | Pentose and glucuronate interconversions | 0.48975654 | Metabolism | Carbohydrate metabolism | 0.48975654 | 7 |
| map00511 | Other glycan degradation | 0.495060185 | Metabolism | Glycan biosynthesis and metabolism | 0.495060185 | 1 |
| map00650 | Butanoate metabolism | 0.509261523 | Metabolism | Carbohydrate metabolism | 0.509261523 | 2 |
| map04141 | Protein processing in endoplasmic reticulum | 0.528443537 | Genetic Information Processing | Folding, sorting and degradation | 0.528443537 | 15 |
| map05165 | Human papillomavirus infection | 0.539991734 | Human Diseases | Infectious diseases: Viral | 0.539991734 | 8 |
| map00909 | Sesquiterpenoid and triterpenoid biosynthesis | 0.545747221 | Metabolism | Metabolism of terpenoids and polyketides | 0.545747221 | 4 |
| map00902 | Monoterpenoid biosynthesis | 0.547050964 | Metabolism | Metabolism of terpenoids and polyketides | 0.547050964 | 3 |
| map00195 | Photosynthesis | 0.572779902 | Metabolism | Energy metabolism | 0.572779902 | 4 |
| map00901 | Indole alkaloid biosynthesis | 0.57646047 | Metabolism | Biosynthesis of other secondary metabolites | 0.57646047 | 2 |
| map00061 | Fatty acid biosynthesis | 0.578370633 | Metabolism | Lipid metabolism | 0.578370633 | 3 |
| map04933 | AGE-RAGE signaling pathway in diabetic complications | 0.579226727 | Human Diseases | Endocrine and metabolic diseases | 0.579226727 | 1 |
| map00270 | Cysteine and methionine metabolism | 0.588973171 | Metabolism | Amino acid metabolism | 0.588973171 | 6 |
| map00941 | Flavonoid biosynthesis | 0.624013635 | Metabolism | Biosynthesis of other secondary metabolites | 0.624013635 | 4 |
| map00780 | Biotin metabolism | 0.649382703 | Metabolism | Metabolism of cofactors and vitamins | 0.649382703 | 1 |
| map00531 | Glycosaminoglycan degradation | 0.649382703 | Metabolism | Glycan biosynthesis and metabolism | 0.649382703 | 1 |
| map04218 | Cellular senescence | 0.656324347 | Cellular Processes | Cell growth and death | 0.656324347 | 6 |
| map00945 | Stilbenoid, diarylheptanoid and gingerol biosynthesis | 0.685970845 | Metabolism | Biosynthesis of other secondary metabolites | 0.685970845 | 4 |
| map00860 | Porphyrin and chlorophyll metabolism | 0.689984224 | Metabolism | Metabolism of cofactors and vitamins | 0.689984224 | 2 |
| map00051 | Fructose and mannose metabolism | 0.705458349 | Metabolism | Carbohydrate metabolism | 0.705458349 | 3 |
| map04145 | Phagosome | 0.733986155 | Cellular Processes | Transport and catabolism | 0.733986155 | 4 |
| map00250 | Alanine, aspartate and glutamate metabolism | 0.745264492 | Metabolism | Amino acid metabolism | 0.745264492 | 2 |
| map00710 | Carbon fixation in photosynthetic organisms | 0.75702125 | Metabolism | Energy metabolism | 0.75702125 | 3 |
| map00565 | Ether lipid metabolism | 0.767450928 | Metabolism | Lipid metabolism | 0.767450928 | 1 |
| map00564 | Glycerophospholipid metabolism | 0.770570794 | Metabolism | Lipid metabolism | 0.770570794 | 4 |
| map04712 | Circadian rhythm - plant | 0.784668288 | Organismal Systems | Environmental adaptation | 0.784668288 | 2 |
| map00196 | Photosynthesis - antenna proteins | 0.797208972 | Metabolism | Energy metabolism | 0.797208972 | 1 |
| map00030 | Pentose phosphate pathway | 0.805653565 | Metabolism | Carbohydrate metabolism | 0.805653565 | 2 |
| map00600 | Sphingolipid metabolism | 0.814903797 | Metabolism | Lipid metabolism | 0.814903797 | 1 |
| map00500 | Starch and sucrose metabolism | 0.825796351 | Metabolism | Carbohydrate metabolism | 0.825796351 | 6 |
| map00460 | Cyanoamino acid metabolism | 0.842235793 | Metabolism | Metabolism of other amino acids | 0.842235793 | 2 |
| map00010 | Glycolysis / Gluconeogenesis | 0.845459331 | Metabolism | Carbohydrate metabolism | 0.845459331 | 5 |
| map00071 | Fatty acid degradation | 0.847688693 | Metabolism | Lipid metabolism | 0.847688693 | 2 |
| map00640 | Propanoate metabolism | 0.847688693 | Metabolism | Carbohydrate metabolism | 0.847688693 | 2 |
| map00592 | alpha-Linolenic acid metabolism | 0.85296974 | Metabolism | Lipid metabolism | 0.85296974 | 2 |
| map00100 | Steroid biosynthesis | 0.865546493 | Metabolism | Lipid metabolism | 0.865546493 | 1 |
| map00590 | Arachidonic acid metabolism | 0.865546493 | Metabolism | Lipid metabolism | 0.865546493 | 1 |
| map03020 | RNA polymerase | 0.867826128 | Genetic Information Processing | Transcription | 0.867826128 | 2 |
| map04146 | Peroxisome | 0.868212072 | Cellular Processes | Transport and catabolism | 0.868212072 | 4 |
| map00220 | Arginine biosynthesis | 0.871550008 | Metabolism | Amino acid metabolism | 0.871550008 | 1 |
| map00062 | Fatty acid elongation | 0.882765999 | Metabolism | Lipid metabolism | 0.882765999 | 1 |
| map00380 | Tryptophan metabolism | 0.889554498 | Metabolism | Amino acid metabolism | 0.889554498 | 2 |
| map00330 | Arginine and proline metabolism | 0.889554498 | Metabolism | Amino acid metabolism | 0.889554498 | 2 |
| map00073 | Cutin, suberine and wax biosynthesis | 0.922300392 | Metabolism | Lipid metabolism | 0.922300392 | 1 |
| map04928 | Parathyroid hormone synthesis, secretion and action | 0.925772781 | Organismal Systems | Endocrine system | 0.925772781 | 1 |
| map00240 | Pyrimidine metabolism | 0.927591869 | Metabolism | Nucleotide metabolism | 0.927591869 | 5 |
| map00591 | Linoleic acid metabolism | 0.935287604 | Metabolism | Lipid metabolism | 0.935287604 | 1 |
| map04934 | Cushing syndrome | 0.935287604 | Human Diseases | Endocrine and metabolic diseases | 0.935287604 | 1 |
| map00908 | Zeatin biosynthesis | 0.95247583 | Metabolism | Metabolism of terpenoids and polyketides | 0.95247583 | 5 |
| map00970 | Aminoacyl-tRNA biosynthesis | 0.957127277 | Genetic Information Processing | Translation | 0.957127277 | 1 |
| map00053 | Ascorbate and aldarate metabolism | 0.96087718 | Metabolism | Carbohydrate metabolism | 0.96087718 | 1 |
| map03018 | RNA degradation | 0.973173742 | Genetic Information Processing | Folding, sorting and degradation | 0.973173742 | 3 |
| map03008 | Ribosome biogenesis in eukaryotes | 0.978252722 | Genetic Information Processing | Translation | 0.978252722 | 2 |
| map00230 | Purine metabolism | 0.979274007 | Metabolism | Nucleotide metabolism | 0.979274007 | 5 |
| map03015 | mRNA surveillance pathway | 0.97973988 | Genetic Information Processing | Translation | 0.97973988 | 3 |
| map03013 | RNA transport | 0.979898334 | Genetic Information Processing | Translation | 0.979898334 | 5 |
| map03430 | Mismatch repair | 0.980443796 | Genetic Information Processing | Replication and repair | 0.980443796 | 3 |
| map00280 | Valine, leucine and isoleucine degradation | 0.986969532 | Metabolism | Amino acid metabolism | 0.986969532 | 1 |
| map00561 | Glycerolipid metabolism | 0.987553506 | Metabolism | Lipid metabolism | 0.987553506 | 1 |
| map03440 | Homologous recombination | 0.991765711 | Genetic Information Processing | Replication and repair | 0.991765711 | 3 |
| map00630 | Glyoxylate and dicarboxylate metabolism | 0.992132518 | Metabolism | Carbohydrate metabolism | 0.992132518 | 1 |
| map00190 | Oxidative phosphorylation | 0.994712784 | Metabolism | Energy metabolism | 0.994712784 | 3 |
| map04217 | Necroptosis | 0.995862941 | Cellular Processes | Cell growth and death | 0.995862941 | 1 |
| map03420 | Nucleotide excision repair | 0.995944376 | Genetic Information Processing | Replication and repair | 0.995944376 | 2 |
| map04714 | Thermogenesis | 0.998390663 | Organismal Systems | Environmental adaptation | 0.998390663 | 4 |
| map03030 | DNA replication | 0.998748269 | Genetic Information Processing | Replication and repair | 0.998748269 | 1 |
| map03040 | Spliceosome | 0.999182793 | Genetic Information Processing | Transcription | 0.999182793 | 3 |
| map04120 | Ubiquitin mediated proteolysis | 0.999244048 | Genetic Information Processing | Folding, sorting and degradation | 0.999244048 | 3 |
