# Supplementary material for: The Induction of Disease Resistance by Scopolamine and the Application of Datura Extract Against Potato (Solanum tuberosum L.) Late Blight
Source: Int J Mol Sci. 2024 Dec 15;25(24):13442. doi: 10.3390/ijms252413442 (PMC11676833; doi:10.3390/ijms252413442)
Supplement: Supplementary file 1 [file ijms-25-13442-s001.zip › Supplementary Table 7.docx]

**Supplementary Table 7 DEGs related to membrane and cell wall**

| gene name | gene description | log_2_FC(S/C) | *P* value |
| --- | --- | --- | --- |
| Plasma membrane | | | |
| Soltu.DM.01G000790 | Plant cadmium resistance | -1.37322227 | 0.04593041 |
| Soltu.DM.01G003160 | Abscisic acid-responsive (TB2/DP1, HVA22) family protein | -1.21006742 | 0.02122811 |
| Soltu.DM.01G004960 | hypothetical protein | -5.16085572 | 0.00788574 |
| Soltu.DM.01G005400 | cytochrome P450, family 71, subfamily B, polypeptide | -2.57503655 | 0.04694314 |
| Soltu.DM.01G006200 | Late embryogenesis abundant (LEA) hydroxyproline-rich glycoprotein family | -1.09289278 | 0.02519381 |
| Soltu.DM.01G006570 | Leucine-rich receptor-like protein kinase family protein | -2.80736566 | 0.00824859 |
| Soltu.DM.01G007940 | potassium channel in Arabidopsis thaliana | 1.9037761 | 0.04087967 |
| Soltu.DM.01G008310 | beta glucosidase | 1.53376494 | 0.03031048 |
| Soltu.DM.01G011140 | receptor-like protein kinase | -2.23619974 | 0.03340279 |
| Soltu.DM.01G020290 | hypothetical protein | 1.43493981 | 0.02976836 |
| Soltu.DM.01G023180 | Leucine-rich receptor-like protein kinase family protein | 1.71961405 | 0.02341788 |
| Soltu.DM.01G026010 | purple acid phosphatase | 1.33498733 | 0.00483793 |
| Soltu.DM.01G027660 | hypothetical protein | -1.14481122 | 0.02142631 |
| Soltu.DM.01G028580 | conserved hypothetical protein | -2.8733903 | 0.0158366 |
| Soltu.DM.01G029840 | Wall-associated receptor kinase galacturonan-binding domain containing protein | 1.56943131 | 0.04478466 |
| Soltu.DM.01G030170 | Ankyrin repeat family protein | -2.95103347 | 0.04227645 |
| Soltu.DM.01G030410 | PRA1 (Prenylated rab acceptor) family protein | -1.79808609 | 0.01489269 |
| Soltu.DM.01G031630 | Plant VAMP (vesicle-associated membrane protein) family protein | -1.10341693 | 0.03294456 |
| Soltu.DM.01G032410 | S-adenosyl-L-methionine-dependent methyltransferases superfamily protein | -1.38561469 | 0.02812159 |
| Soltu.DM.01G032830 | early nodulin-like protein | -1.1123741 | 0.02893129 |
| Soltu.DM.01G034630 | manganese tracking factor for mitochondrial SOD2 | -1.28856154 | 0.01367529 |
| Soltu.DM.01G036640 | conserved hypothetical protein | -2.87651935 | 0.01001094 |
| Soltu.DM.01G038350 | Signal peptidase subunit | -1.11690877 | 0.03777928 |
| Soltu.DM.01G038640 | disease resistance family protein / LRR family protein | 2.15050202 | 0.0413963 |
| Soltu.DM.01G040150 | Inorganic H pyrophosphatase family protein | -2.06210391 | 0.04064511 |
| Soltu.DM.01G042240 | plasma membrane intrinsic protein 1;5 | 4.57465608 | 0.02437512 |
| Soltu.DM.01G046850 | LYR motif-containing protein At3g19508 | -1.19728784 | 0.04796614 |
| Soltu.DM.01G047220 | Protein of unknown function (DUF579) | 1.40702798 | 0.02259908 |
| Soltu.DM.02G012990 | Leucine-rich receptor-like protein kinase family protein | 1.07731559 | 0.04266489 |
| Soltu.DM.02G019250 | receptor kinase | -1.4985975 | 0.03839046 |
| Soltu.DM.02G019570 | cysteine-rich RLK (RECEPTOR-like protein kinase) | -2.22050571 | 0.00790146 |
| Soltu.DM.02G019620 | cysteine-rich RLK (RECEPTOR-like protein kinase) | -1.49410823 | 0.04228594 |
| Soltu.DM.02G019820 | methylesterase PCR A | 1.63621905 | 0.01194014 |
| Soltu.DM.02G019840 | methylesterase PCR A | 2.10953714 | 0.00432268 |
| Soltu.DM.02G020370 | F-box family protein with a domain of unknown function (DUF295) | -1.91291131 | 0.03449208 |
| Soltu.DM.02G020420 | aspartic proteinase A1 | 1.32670518 | 0.00760924 |
| Soltu.DM.02G020930 | microsomal glutathione s-transferase, putative | -1.6107509 | 0.02603548 |
| Soltu.DM.02G021070 | Protein kinase superfamily protein | -1.9916752 | 0.0282497 |
| Soltu.DM.02G021100 | receptor serine/threonine kinase, putative | -3.10267661 | 0.01718472 |
| Soltu.DM.02G022950 | TRICHOME BIREFRINGENCE-LIKE | -1.61396961 | 0.02169847 |
| Soltu.DM.02G023340 | NEP-interacting protein | -1.31233843 | 0.00271015 |
| Soltu.DM.02G023620 | histidine kinase | 1.03932612 | 0.02961573 |
| Soltu.DM.02G024350 | Protein of unknown function (DUF726) | 1.44226427 | 0.04020575 |
| Soltu.DM.02G024530 | beta-galactosidase | 2.06040581 | 0.02046644 |
| Soltu.DM.02G025220 | Cytochrome P450 superfamily protein | 1.56383437 | 0.02954422 |
| Soltu.DM.02G026090 | Protein kinase superfamily protein | -1.48696381 | 0.02595313 |
| Soltu.DM.02G026510 | hypothetical protein | -1.10354959 | 0.01386717 |
| Soltu.DM.02G026700 | conserved hypothetical protein | -2.03096417 | 0.0285746 |
| Soltu.DM.02G028120 | cytochrome P450, family 81, subfamily D, polypeptide | -1.86542476 | 0.01251436 |
| Soltu.DM.02G029910 | protein kinase family protein / peptidoglycan-binding LysM domain-containing protein | -1.67276786 | 0.01698463 |
| Soltu.DM.02G031740 | Protein of unknown function (DUF1666) | -1.47107453 | 0.018354 |
| Soltu.DM.02G032330 | ABC transporter family protein | 1.40270515 | 0.02495863 |
| Soltu.DM.02G032490 | Family of unknown function (DUF716) | -1.25181985 | 0.00986327 |
| Soltu.DM.02G033820 | Domain of unknown function (DUF23) | -1.10588669 | 0.02440484 |
| Soltu.DM.03G000760 | translocase of outer membrane 22-V | -1.41125029 | 0.01693325 |
| Soltu.DM.03G000830 | chlorophyll A/B binding protein | 1.915808 | 0.01319258 |
| Soltu.DM.03G001940 | receptor like protein | 1.45591919 | 0.04728172 |
| Soltu.DM.03G002400 | Protein kinase superfamily protein | -1.02060068 | 0.0467118 |
| Soltu.DM.03G004500 | abscisic acid (aba)-deficient | -1.22700345 | 0.00298734 |
| Soltu.DM.03G004580 | Peptidase M50B-like domain containing protein | -1.23790767 | 0.01514405 |
| Soltu.DM.03G005020 | hydroxyproline-rich glycoprotein family protein | 2.37354398 | 0.00198335 |
| Soltu.DM.03G005390 | heptahelical protein | -1.66401572 | 0.01006931 |
| Soltu.DM.03G012810 | plasma membrane intrinsic protein 1;4 | 4.35364371 | 0.0005338 |
| Soltu.DM.03G013630 | RING/U-box superfamily protein | -1.20925558 | 0.0159805 |
| Soltu.DM.03G014650 | Protein kinase superfamily protein | 1.50730199 | 0.0103622 |
| Soltu.DM.03G015700 | EXORDIUM like | 1.10524449 | 0.03894844 |
| Soltu.DM.03G016610 | peptidoglycan-binding LysM domain-containing protein | -2.99924457 | 0.00248725 |
| Soltu.DM.03G017800 | cytochrome P450, family 715, subfamily A, polypeptide | -2.52646147 | 0.02049665 |
| Soltu.DM.03G018440 | Kunitz family trypsin and protease inhibitor protein | -1.29388352 | 0.03460361 |
| Soltu.DM.03G018820 | Protein of unknown function (DUF761) | 2.41740536 | 0.04787342 |
| Soltu.DM.03G020240 | hypothetical protein | 1.81616526 | 0.01129653 |
| Soltu.DM.03G020790 | 4-coumarate:CoA ligase | -1.61713838 | 0.02859725 |
| Soltu.DM.03G020890 | Cytochrome P450 superfamily protein | -2.00762581 | 0.02528875 |
| Soltu.DM.03G024780 | Xanthine/uracil permease family protein | 1.63846027 | 0.04716116 |
| Soltu.DM.03G024870 | RING/U-box superfamily protein | -3.26858897 | 0.0036232 |
| Soltu.DM.03G024920 | cysteine-rich RLK (RECEPTOR-like protein kinase) | -1.51408313 | 0.03220943 |
| Soltu.DM.03G025510 | cytochrome P450, family 71, subfamily A, polypeptide | -1.86525523 | 0.0370194 |
| Soltu.DM.03G025600 | cytochrome P450, family 71, subfamily A, polypeptide | -1.93160382 | 0.02124991 |
| Soltu.DM.03G027150 | nitrate transporter 1:2 | 2.7118917 | 0.01165784 |
| Soltu.DM.03G027800 | Glycosyl hydrolase family 47 protein | -1.36739785 | 0.04825769 |
| Soltu.DM.03G028860 | O-fucosyltransferase family protein | 1.38305338 | 0.0031353 |
| Soltu.DM.03G030970 | natural resistance-associated macrophage protein | 1.25373337 | 0.04249486 |
| Soltu.DM.03G032730 | receptor-like kinase | 1.68765607 | 0.01425022 |
| Soltu.DM.03G034460 | conserved hypothetical protein | -2.73048885 | 0.00069183 |
| Soltu.DM.03G035700 | downstream target of AGL15-4 | 1.90218171 | 0.03174368 |
| Soltu.DM.03G035890 | actin binding protein family | 1.7023679 | 0.00929418 |
| Soltu.DM.03G036080 | Calcium-activated chloride channel domain containing protein | 1.78498652 | 0.01141857 |
| Soltu.DM.03G037470 | RING/U-box superfamily protein | -1.45540924 | 0.02390132 |
| Soltu.DM.04G000060 | Matrixin family protein | -1.68891464 | 0.02248227 |
| Soltu.DM.04G002040 | isopentenyltransferase | -3.3729408 | 4.56E-06 |
| Soltu.DM.04G002100 | conserved hypothetical protein | -1.74829541 | 0.01503064 |
| Soltu.DM.04G002250 | Protein kinase superfamily protein | -1.31345675 | 0.04156804 |
| Soltu.DM.04G002270 | Protein kinase superfamily protein | -1.88688313 | 0.01939959 |
| Soltu.DM.04G002660 | proline-rich family protein | 1.77989187 | 0.02494795 |
| Soltu.DM.04G003290 | PLANT CADMIUM RESISTANCE | -1.29696508 | 0.00175893 |
| Soltu.DM.04G004090 | BRI1-like | 1.62422518 | 0.01337131 |
| Soltu.DM.04G008450 | Leucine-rich repeat transmembrane protein kinase | 1.25175266 | 0.04723787 |
| Soltu.DM.04G010650 | Leucine-rich repeat protein kinase family protein | 2.29036941 | 0.00202624 |
| Soltu.DM.04G013500 | Plant protein of unknown function (DUF247) | 2.35486189 | 0.00823387 |
| Soltu.DM.04G013670 | GDSL lipase | 1.22351048 | 0.02739097 |
| Soltu.DM.04G020160 | Protein kinase superfamily protein | 1.50757798 | 0.04219409 |
| Soltu.DM.04G020510 | conserved hypothetical protein | -2.06661157 | 0.00907856 |
| Soltu.DM.04G020610 | cytochrome P450, family 71, subfamily A, polypeptide | -1.89138259 | 0.02158957 |
| Soltu.DM.04G020740 | Leucine-rich repeat (LRR) family protein | 1.24773185 | 0.04254754 |
| Soltu.DM.04G021440 | Major facilitator superfamily protein | -1.35734001 | 0.03844136 |
| Soltu.DM.04G021850 | slufate transporter 2;1 | 2.72370555 | 1.72E-06 |
| Soltu.DM.04G021930 | Cupredoxin superfamily protein | -1.81344887 | 0.01561659 |
| Soltu.DM.04G022150 | Cupredoxin superfamily protein | -1.69089281 | 0.01852772 |
| Soltu.DM.04G022660 | cytochrome P450, family 71, subfamily A, polypeptide | -2.69655193 | 0.03244331 |
| Soltu.DM.04G024000 | nucleotide sugar transporter-KT | -1.37881537 | 0.02677674 |
| Soltu.DM.04G025520 | conserved hypothetical protein | -2.47898002 | 0.00282081 |
| Soltu.DM.04G027140 | Protein of unknown function (DUF793) | -1.32986927 | 0.02343246 |
| Soltu.DM.04G027620 | Protein kinase superfamily protein | -1.01430426 | 0.01648953 |
| Soltu.DM.04G028320 | laccase | -3.65587425 | 0.04361932 |
| Soltu.DM.04G028470 | RING/U-box superfamily protein | -2.58259459 | 0.00975812 |
| Soltu.DM.04G029040 | Glycosyl hydrolase family protein | 1.4248981 | 0.00190099 |
| Soltu.DM.04G030340 | RING/U-box superfamily protein | -1.53051046 | 0.01337322 |
| Soltu.DM.04G031280 | HVA22 homologue A | 1.15197253 | 0.02798714 |
| Soltu.DM.04G031480 | oligopeptide transporter | 1.56955695 | 0.00430993 |
| Soltu.DM.04G031720 | Leucine-rich repeat transmembrane protein kinase family protein | 1.66226321 | 0.03773993 |
| Soltu.DM.04G032040 | protein serine/threonine kinases;protein kinases;ATP binding;sugar binding;kinases;carbohydrate binding | -1.12128122 | 0.03031444 |
| Soltu.DM.04G032170 | Transmembrane amino acid transporter family protein | -1.29794575 | 0.02229736 |
| Soltu.DM.04G033040 | cytochrome P450, family 81, subfamily D, polypeptide | -1.50096462 | 0.02524259 |
| Soltu.DM.04G033190 | Domain of unknown function (DUF303) | -4.18851335 | 0.00161258 |
| Soltu.DM.04G034290 | Serine protease inhibitor (SERPIN) family protein | -1.39806697 | 0.00543428 |
| Soltu.DM.04G034410 | Serine protease inhibitor (SERPIN) family protein | -1.56514988 | 0.02397884 |
| Soltu.DM.04G034450 | Major facilitator superfamily protein | 1.10333282 | 0.0464838 |
| Soltu.DM.04G034820 | GNS1/SUR4 membrane protein family | -2.13284781 | 0.02107899 |
| Soltu.DM.04G034830 | lysophosphatidyl acyltransferase | -1.50509179 | 0.01017391 |
| Soltu.DM.04G036490 | C2H2-like zinc finger protein | 2.11158281 | 0.00274915 |
| Soltu.DM.04G037830 | glutamate receptor 3.3 | 1.13014646 | 0.00725829 |
| Soltu.DM.05G000240 | conserved hypothetical protein | -1.18581673 | 0.02006723 |
| Soltu.DM.05G000280 | DHHC-type zinc finger family protein | 1.57566362 | 0.00761139 |
| Soltu.DM.05G000760 | ureide permease | 1.28411973 | 0.00864947 |
| Soltu.DM.05G001680 | hypothetical protein | -1.67216765 | 0.01651297 |
| Soltu.DM.05G002210 | ATPase E1-E2 type family protein / haloacid dehalogenase-like hydrolase family protein | -1.02903587 | 0.0471839 |
| Soltu.DM.05G002780 | PLAC8 family protein | -3.06446947 | 0.01116843 |
| Soltu.DM.05G002800 | PLAC8 family protein | -4.34643471 | 0.04229437 |
| Soltu.DM.05G003040 | conserved hypothetical protein | -1.02986362 | 0.04490743 |
| Soltu.DM.05G003410 | Protein kinase superfamily protein | -2.69466409 | 0.0162664 |
| Soltu.DM.05G003720 | Protein kinase superfamily protein | -1.43050893 | 0.03385106 |
| Soltu.DM.05G004430 | kokopelli | 1.56805074 | 0.00637811 |
| Soltu.DM.05G004570 | conserved hypothetical protein | 1.55490777 | 0.00384715 |
| Soltu.DM.05G005090 | galactosyltransferase1 | -1.53061863 | 0.0301291 |
| Soltu.DM.05G006230 | UDP-galactose transporter | -1.6501722 | 0.02128168 |
| Soltu.DM.05G006290 | Leucine-rich repeat protein kinase family protein | 1.32261434 | 0.03655375 |
| Soltu.DM.05G007790 | Major facilitator superfamily protein | 2.01905513 | 0.02643909 |
| Soltu.DM.05G008020 | RING/U-box superfamily protein | 1.1613859 | 0.02498328 |
| Soltu.DM.05G008050 | NHL domain-containing protein | 1.68657714 | 0.03186246 |
| Soltu.DM.05G009380 | hercules receptor kinase | -1.13412173 | 0.01207825 |
| Soltu.DM.05G009530 | Protein of Unknown Function (DUF239) | 2.18048399 | 0.03127768 |
| Soltu.DM.05G009780 | root hair specific | 2.7803669 | 0.04928296 |
| Soltu.DM.05G009860 | phosphate transporter 2;1 | 1.30067202 | 0.02364919 |
| Soltu.DM.05G010490 | Leucine-rich repeat protein kinase family protein | 1.22574189 | 0.0189586 |
| Soltu.DM.05G017910 | Auxin-responsive family protein | -1.61289275 | 0.01331224 |
| Soltu.DM.05G019270 | cytochrome p450 78a9 | 1.69500349 | 0.00047458 |
| Soltu.DM.05G020010 | cyclic nucleotide gated channel | 1.29390692 | 0.00165847 |
| Soltu.DM.05G020050 | cyclic nucleotide gated channel | -1.93120234 | 0.0101521 |
| Soltu.DM.05G021160 | uncoupling protein | -1.27616133 | 0.0126313 |
| Soltu.DM.05G021330 | white-brown complex homolog protein | -1.8614301 | 0.01101851 |
| Soltu.DM.05G021350 | white-brown complex homolog protein | -1.59855622 | 0.00503182 |
| Soltu.DM.05G021360 | white-brown complex homolog protein | -1.53603849 | 0.02247884 |
| Soltu.DM.05G021590 | Leucine-rich repeat protein kinase family protein | 2.13518642 | 0.00996087 |
| Soltu.DM.05G022180 | zinc-binding in reverse transcriptase domain containing protein | 1.89566788 | 0.02433442 |
| Soltu.DM.05G022780 | Alpha/beta hydrolase related protein | -1.49957269 | 0.03944598 |
| Soltu.DM.05G023050 | Pathogenesis-related thaumatin superfamily protein | 1.41456839 | 0.04729003 |
| Soltu.DM.05G024190 | Transmembrane amino acid transporter family protein | 2.90494713 | 0.0336202 |
| Soltu.DM.05G025060 | Putative membrane lipoprotein | 1.26409851 | 0.03574272 |
| Soltu.DM.05G025360 | ALA-interacting subunit | -1.09994856 | 0.0244596 |
| Soltu.DM.05G026670 | Ankyrin repeat family protein | -2.10250867 | 0.00926262 |
| Soltu.DM.06G001620 | Leucine-rich repeat receptor-like protein kinase family protein | -4.72457479 | 0.00770943 |
| Soltu.DM.06G003080 | Plant protein of unknown function (DUF828) | 1.57744524 | 0.00950252 |
| Soltu.DM.06G003660 | copper transporter | -2.01418947 | 0.01475855 |
| Soltu.DM.06G004000 | ZRT/IRT-like protein | -2.42485221 | 0.03473707 |
| Soltu.DM.06G009010 | expansin A4 | 1.60619334 | 0.01373187 |
| Soltu.DM.06G009380 | CRINKLY4 related | -1.31144863 | 0.03613886 |
| Soltu.DM.06G010960 | potassium transporter | 1.83998432 | 0.01541164 |
| Soltu.DM.06G011440 | RING/U-box superfamily protein | 2.27593074 | 0.01500813 |
| Soltu.DM.06G011560 | Leucine-rich repeat protein kinase family protein | 1.57484915 | 0.005121 |
| Soltu.DM.06G012630 | EXORDIUM like | 1.70281945 | 0.03288005 |
| Soltu.DM.06G013450 | beta-carotene hydroxylase | -1.07306895 | 0.01721595 |
| Soltu.DM.06G013790 | Plant invertase/pectin methylesterase inhibitor superfamily | 2.38243806 | 0.0149575 |
| Soltu.DM.06G014150 | Lung seven transmembrane receptor family protein | -2.52381286 | 0.00338554 |
| Soltu.DM.06G019860 | 3-ketoacyl-CoA synthase | -1.12627564 | 0.03349055 |
| Soltu.DM.06G020490 | late embryogenesis abundant domain-containing protein / LEA domain-containing protein | -3.55120957 | 0.0018388 |
| Soltu.DM.06G020750 | P-loop containing nucleoside triphosphate hydrolases superfamily protein | -2.06041202 | 0.02072969 |
| Soltu.DM.06G021930 | glutamate receptor 2.8 | -3.60795916 | 0.00302081 |
| Soltu.DM.06G021980 | glutamate receptor 2.9 | -2.68096805 | 0.00997886 |
| Soltu.DM.06G022050 | glutamate receptor 2.8 | -2.05033118 | 0.03881592 |
| Soltu.DM.06G022070 | glutamate receptor 2.8 | -3.28760189 | 0.00340589 |
| Soltu.DM.06G022080 | glutamate receptor 2.8 | -2.12708649 | 0.02972024 |
| Soltu.DM.06G024280 | phospholipid N-methyltransferase | -1.07354264 | 0.04352509 |
| Soltu.DM.06G025870 | RING/FYVE/PHD zinc finger superfamily protein | -1.27812691 | 0.03303839 |
| Soltu.DM.06G026640 | HCO3- transporter family | 1.47288861 | 0.01412548 |
| Soltu.DM.06G027220 | secE/sec61-gamma protein transport protein | -1.08993097 | 0.02939183 |
| Soltu.DM.06G028340 | Pathogenesis-related thaumatin superfamily protein | 5.05697445 | 0.01549242 |
| Soltu.DM.06G028360 | serine protease inhibitor, Kazal-type family protein | -1.03416124 | 0.03856988 |
| Soltu.DM.06G029040 | Bifunctional inhibitor/lipid-transfer protein/seed storage 2S albumin superfamily protein | 1.47692982 | 0.0141254 |
| Soltu.DM.06G030000 | cellulose synthase-like A02 | 1.42238813 | 0.03862879 |
| Soltu.DM.06G030840 | nitrate excretion transporter1 | 1.26694654 | 0.03092862 |
| Soltu.DM.06G031040 | P-loop containing nucleoside triphosphate hydrolases superfamily protein | 1.45962862 | 0.01762786 |
| Soltu.DM.06G031570 | Rab5-interacting family protein | -1.12455981 | 0.03769113 |
| Soltu.DM.06G032750 | SOS3-interacting protein | -1.9415507 | 0.00506017 |
| Soltu.DM.06G033580 | ER lumen protein retaining receptor family protein | -1.92804389 | 0.01039361 |
| Soltu.DM.06G033590 | ER lumen protein retaining receptor family protein | -2.25593188 | 0.01800735 |
| Soltu.DM.07G000020 | Leucine-rich repeat protein kinase family protein | 1.51646493 | 0.00708375 |
| Soltu.DM.07G000180 | receptor kinase | -3.824267 | 0.02579076 |
| Soltu.DM.07G000580 | Leucine-rich repeat protein kinase family protein | 1.30174278 | 0.02817797 |
| Soltu.DM.07G000770 | Low temperature and salt responsive protein family | -1.00773648 | 0.01400242 |
| Soltu.DM.07G001250 | cytochrome P450, family 72, subfamily A, polypeptide | -1.11652142 | 0.02225827 |
| Soltu.DM.07G001330 | UDP-D-glucuronate 4-epimerase | 1.01474543 | 0.01423331 |
| Soltu.DM.07G002120 | UDP-glucosyl transferase 73B5 | -2.1852053 | 0.00253252 |
| Soltu.DM.07G002180 | xyloglucan endotransglucosylase/hydrolase | 1.72163285 | 0.01708283 |
| Soltu.DM.07G002460 | zinc transporter of Arabidopsis thaliana | -4.48996965 | 0.04511981 |
| Soltu.DM.07G003570 | blue-copper-binding protein | -1.5365407 | 0.04275537 |
| Soltu.DM.07G004010 | purine permease | 1.04717883 | 0.02261223 |
| Soltu.DM.07G004270 | receptor like protein | -1.41790645 | 0.04193978 |
| Soltu.DM.07G005310 | S-locus lectin protein kinase family protein | -1.31389736 | 0.03783745 |
| Soltu.DM.07G005320 | S-locus lectin protein kinase family protein | -1.55090723 | 0.01070432 |
| Soltu.DM.07G005660 | ADP-ribosylation factor family protein | 3.24661957 | 0.04549568 |
| Soltu.DM.07G006960 | EF-TU receptor | 1.46622885 | 0.02078061 |
| Soltu.DM.07G013260 | conserved hypothetical protein | -1.53035106 | 0.03165777 |
| Soltu.DM.07G014660 | glycosyltransferase | -1.25627525 | 0.01748526 |
| Soltu.DM.07G015350 | conserved hypothetical protein | -1.57965162 | 0.0102217 |
| Soltu.DM.07G016110 | Auxin-responsive family protein | -2.24233213 | 0.0081349 |
| Soltu.DM.07G016120 | Auxin-responsive family protein | -2.71330289 | 0.0082951 |
| Soltu.DM.07G016130 | Auxin-responsive family protein | -2.01071786 | 0.02712478 |
| Soltu.DM.07G017310 | SLAC1 homologue | -1.86835638 | 0.04879404 |
| Soltu.DM.07G017850 | Transmembrane protein 97, predicted | -1.10103748 | 0.04972879 |
| Soltu.DM.07G018590 | S-locus lectin protein kinase family protein | -1.24794189 | 0.00915163 |
| Soltu.DM.07G018600 | S-locus lectin protein kinase family protein | -1.19180884 | 0.02139081 |
| Soltu.DM.07G019710 | expansin B3 | 2.34065503 | 0.01477384 |
| Soltu.DM.07G020440 | RING/U-box superfamily protein | -1.02173314 | 0.04255235 |
| Soltu.DM.07G021360 | cytochrome P450, family 72, subfamily A, polypeptide | -1.9077963 | 0.01755407 |
| Soltu.DM.07G021370 | cytochrome P450, family 72, subfamily A, polypeptide | -1.67919474 | 0.0298447 |
| Soltu.DM.07G021390 | cytochrome P450, family 72, subfamily A, polypeptide | 2.7353111 | 0.02887801 |
| Soltu.DM.07G021660 | S-domain-2 | -1.66207277 | 0.01644369 |
| Soltu.DM.07G022060 | Carbohydrate-binding X8 domain superfamily protein | 2.34078544 | 0.01828518 |
| Soltu.DM.07G022270 | Cytochrome P450 superfamily protein | 2.40745609 | 0.00391932 |
| Soltu.DM.07G023160 | Protein kinase superfamily protein | 1.5242709 | 0.03708793 |
| Soltu.DM.07G025400 | formin homology5 | 2.47131689 | 0.01592159 |
| Soltu.DM.07G025750 | early nodulin-like protein | 1.94505705 | 0.00918157 |
| Soltu.DM.07G027700 | Leucine-rich receptor-like protein kinase family protein | 1.71294962 | 0.01869624 |
| Soltu.DM.08G000040 | MATE efflux family protein | -2.14654524 | 0.03753096 |
| Soltu.DM.08G000320 | DUF2499 domain containing protein | 1.76196686 | 0.04884885 |
| Soltu.DM.08G000480 | Bifunctional inhibitor/lipid-transfer protein/seed storage 2S albumin superfamily protein | -1.82049159 | 0.02136528 |
| Soltu.DM.08G000560 | plant VAP homolog | 1.89002939 | 0.02409445 |
| Soltu.DM.08G001690 | Endomembrane protein 70 protein family | -1.12773653 | 0.03835435 |
| Soltu.DM.08G002310 | Cellulose-synthase-like C5 | 1.72193427 | 0.02212651 |
| Soltu.DM.08G003320 | ATP-binding cassette | 1.87576811 | 0.04754139 |
| Soltu.DM.08G003390 | Amino acid permease family protein | 1.45387409 | 0.04485018 |
| Soltu.DM.08G004540 | Late embryogenesis abundant (LEA) hydroxyproline-rich glycoprotein family | -2.18000881 | 0.03527642 |
| Soltu.DM.08G005670 | C2 domain-containing protein / GRAM domain-containing protein | -6.19333962 | 0.00745719 |
| Soltu.DM.08G005970 | conserved hypothetical protein | 2.28153491 | 0.04809853 |
| Soltu.DM.08G013640 | ferrochelatase | -1.2383814 | 0.02446447 |
| Soltu.DM.08G014550 | Protein kinase family protein with leucine-rich repeat domain | -2.59361901 | 0.01881558 |
| Soltu.DM.08G014810 | Leucine-rich receptor-like protein kinase family protein | 2.31384104 | 0.00079326 |
| Soltu.DM.08G018140 | Eukaryotic aspartyl protease family protein | -2.70844209 | 0.0226686 |
| Soltu.DM.08G018550 | cyclic nucleotide-gated channel | 3.52066194 | 0.02729067 |
| Soltu.DM.08G020300 | ATP-binding cassette | 1.8099185 | 0.00890312 |
| Soltu.DM.08G020450 | solute:sodium symporters;urea transmembrane transporters | -2.69126418 | 0.02846516 |
| Soltu.DM.08G021530 | Family of unknown function (DUF716) domain containing protein | -1.23317531 | 0.02122385 |
| Soltu.DM.08G021780 | lectin protein kinase family protein | -1.39431873 | 0.01829142 |
| Soltu.DM.08G022670 | Tetraspanin family protein | 1.81261288 | 0.04254077 |
| Soltu.DM.08G023920 | BAX inhibitor | -1.27108175 | 0.00617789 |
| Soltu.DM.08G025360 | Glycosyl hydrolases family 32 protein | -3.47658324 | 0.00681949 |
| Soltu.DM.08G025570 | cytochrome P450, family 706, subfamily A, polypeptide | 1.93099936 | 0.02567642 |
| Soltu.DM.08G025830 | copper amine oxidase family protein | 2.7993244 | 0.01671729 |
| Soltu.DM.08G025880 | UDP-D-glucuronate 4-epimerase | -1.41385261 | 0.03474158 |
| Soltu.DM.08G026960 | MATE efflux family protein | -3.39995919 | 0.0077035 |
| Soltu.DM.08G027650 | root hair specific | -1.14673959 | 0.00551378 |
| Soltu.DM.08G028450 | hypothetical protein | -2.62319948 | 0.00536571 |
| Soltu.DM.08G028460 | Late embryogenesis abundant (LEA) hydroxyproline-rich glycoprotein family | -2.97713176 | 0.01135043 |
| Soltu.DM.08G029320 | Glycine-rich protein family | 1.94731523 | 0.04025449 |
| Soltu.DM.08G029590 | RING/U-box superfamily protein | 2.82306388 | 0.00065277 |
| Soltu.DM.09G000480 | Transmembrane amino acid transporter family protein | 1.16684719 | 0.00666333 |
| Soltu.DM.09G002400 | Oligosaccaryltransferase | -1.04542174 | 0.03190205 |
| Soltu.DM.09G004290 | Nucleotide-diphospho-sugar transferases superfamily protein | 2.64165173 | 0.00813849 |
| Soltu.DM.09G005200 | Pectin lyase-like superfamily protein | 2.7097243 | 0.01520333 |
| Soltu.DM.09G005740 | Calcium-dependent lipid-binding (CaLB domain) family protein | -1.53499354 | 0.00531101 |
| Soltu.DM.09G005860 | plasma membrane intrinsic protein 2;4 | 2.93714597 | 0.00016677 |
| Soltu.DM.09G005870 | plasma membrane intrinsic protein 2A | 3.31093989 | 7.87E-05 |
| Soltu.DM.09G007110 | conserved hypothetical protein | 5.11355888 | 0.04741716 |
| Soltu.DM.09G008770 | receptor like protein | -1.83918962 | 0.00732501 |
| Soltu.DM.09G009300 | prenylated RAB acceptor 1.B4 | -1.09464794 | 0.01634239 |
| Soltu.DM.09G015150 | formin homology | 1.08038448 | 0.00970312 |
| Soltu.DM.09G015240 | Protein of unknown function (DUF707) | -2.16894418 | 0.00176971 |
| Soltu.DM.09G015690 | SKU5 similar | 1.25053997 | 0.03670275 |
| Soltu.DM.09G018170 | vesicle-associated membrane protein | -1.46064615 | 0.01193086 |
| Soltu.DM.09G018580 | Integral membrane HRF1 family protein | -1.07426369 | 0.0066471 |
| Soltu.DM.09G018950 | Leucine-rich repeat transmembrane protein kinase family protein | -3.66748916 | 0.03320587 |
| Soltu.DM.09G019550 | Plant protein of unknown function (DUF828) | 1.86269644 | 0.00634922 |
| Soltu.DM.09G020220 | Bax inhibitor-1 family protein | -1.21168267 | 0.0095148 |
| Soltu.DM.09G022060 | Pollen Ole e 1 allergen and extensin family protein | 4.95060041 | 0.01485371 |
| Soltu.DM.09G023280 | TSPO(outer membrane tryptophan-rich sensory protein)-related | -1.04040265 | 0.03182578 |
| Soltu.DM.09G023860 | cellulose synthase-like D5 | 1.71303476 | 0.02258219 |
| Soltu.DM.09G024250 | receptor-like protein kinase | -3.653172 | 0.00130231 |
| Soltu.DM.09G024270 | amine oxidase | -4.29474942 | 0.02689095 |
| Soltu.DM.09G024650 | Bifunctional inhibitor/lipid-transfer protein/seed storage 2S albumin superfamily protein | -2.6099369 | 0.01563488 |
| Soltu.DM.09G024960 | conserved hypothetical protein | -2.2606268 | 0.00415952 |
| Soltu.DM.09G025290 | ATPase E1-E2 type family protein / haloacid dehalogenase-like hydrolase family protein | -1.66530005 | 0.04557048 |
| Soltu.DM.09G026100 | Target SNARE coiled-coil domain protein | 1.06392517 | 0.03770009 |
| Soltu.DM.09G027420 | ammonium transporter 1;1 | -1.37615134 | 0.01799039 |
| Soltu.DM.09G028080 | Serinc-domain containing serine and sphingolipid biosynthesis protein | 3.87433948 | 0.03979292 |
| Soltu.DM.09G029520 | Disease resistance protein (CC-NBS-LRR class) family | 5.34153951 | 0.00366156 |
| Soltu.DM.09G030850 | cytochrome P450, family 76, subfamily C, polypeptide | -2.39518896 | 0.02999252 |
| Soltu.DM.09G031000 | Ankyrin repeat family protein | 4.72956743 | 0.02357874 |
| Soltu.DM.10G000590 | Cation efflux family protein | -1.76835912 | 0.02572233 |
| Soltu.DM.10G001270 | hypothetical protein | 5.04577115 | 0.00104827 |
| Soltu.DM.10G003670 | cytochrome P450, family 72, subfamily A, polypeptide | -1.86506953 | 0.03863552 |
| Soltu.DM.10G005720 | arabinogalactan protein | -1.15984134 | 0.03026565 |
| Soltu.DM.10G005810 | Fatty acid/sphingolipid desaturase | -1.32243017 | 0.01530077 |
| Soltu.DM.10G009910 | conserved hypothetical protein | 1.87661536 | 0.01274136 |
| Soltu.DM.10G013190 | Concanavalin A-like lectin protein kinase family protein | -3.25120519 | 0.00476933 |
| Soltu.DM.10G015470 | cytochrome P450, family 72, subfamily A, polypeptide | -1.61491796 | 0.00743008 |
| Soltu.DM.10G016100 | B-cell receptor-associated 31-like | 3.25870485 | 0.00814326 |
| Soltu.DM.10G018320 | dicarboxylate transport 2.1 | 2.03596499 | 0.03208549 |
| Soltu.DM.10G018490 | Leucine-rich repeat protein kinase family protein | 1.35241299 | 0.00846767 |
| Soltu.DM.10G019260 | prenylated RAB acceptor 1.B4 | -1.25769326 | 0.01538369 |
| Soltu.DM.10G019330 | TRICHOME BIREFRINGENCE-LIKE | -3.37022238 | 0.02445554 |
| Soltu.DM.10G019760 | alpha 1,4-glycosyltransferase family protein | -2.07626383 | 0.01014024 |
| Soltu.DM.10G020380 | hypothetical protein | 4.56267848 | 0.03222772 |
| Soltu.DM.10G021850 | NB-ARC domain-containing disease resistance protein | 1.33703442 | 0.01766688 |
| Soltu.DM.10G022920 | Arabidopsis Inositol phosphorylceramide synthase | 1.85444291 | 0.0068728 |
| Soltu.DM.10G024050 | plasma membrane intrinsic protein 2A | -1.92079815 | 0.02850607 |
| Soltu.DM.10G024710 | cytochrome P450, family 76, subfamily G, polypeptide | -2.25265686 | 0.01948541 |
| Soltu.DM.10G024730 | cytochrome P450, family 76, subfamily G, polypeptide | -2.518994 | 0.02018195 |
| Soltu.DM.10G025370 | NDR1/HIN1-like | -1.84591216 | 0.00839634 |
| Soltu.DM.10G025380 | NDR1/HIN1-like | -2.3039974 | 0.01396355 |
| Soltu.DM.10G025810 | cytochrome P450, family 82, subfamily C, polypeptide | 2.17779504 | 0.02446718 |
| Soltu.DM.10G026880 | Concanavalin A-like lectin protein kinase family protein | -2.14357041 | 0.01443073 |
| Soltu.DM.10G027330 | Glycosyltransferase family 61 protein | -1.34893058 | 0.02766915 |
| Soltu.DM.10G028420 | squalene synthase | 1.95149268 | 0.00065469 |
| Soltu.DM.10G030130 | zinc finger (C3HC4-type RING finger) family protein | 1.13298704 | 0.01009388 |
| Soltu.DM.11G000080 | Plant protein of unknown function (DUF827) | -2.29610258 | 0.00250191 |
| Soltu.DM.11G001250 | nonsense-mediated mRNA decay NMD3 family protein | -1.00426596 | 0.02842931 |
| Soltu.DM.11G005440 | UDP-Glycosyltransferase superfamily protein | -1.02825032 | 0.04415926 |
| Soltu.DM.11G006330 | Integral membrane Yip1 family protein | -1.06637125 | 0.00615466 |
| Soltu.DM.11G006920 | cationic amino acid transporter | 2.2111037 | 0.00831661 |
| Soltu.DM.11G007740 | hypothetical protein | -4.36757226 | 0.00245321 |
| Soltu.DM.11G008380 | receptor-like protein kinase | -1.6553974 | 0.01997811 |
| Soltu.DM.11G008440 | receptor-like protein kinase | -1.83466073 | 0.0252115 |
| Soltu.DM.11G008450 | receptor-like protein kinase | -1.80958381 | 0.02892468 |
| Soltu.DM.11G008490 | receptor-like protein kinase | -1.66796117 | 0.02616715 |
| Soltu.DM.11G008510 | receptor-like protein kinase | -1.75961645 | 0.0077051 |
| Soltu.DM.11G008890 | conserved hypothetical protein | 1.81417029 | 0.01522936 |
| Soltu.DM.11G010180 | sucrose-proton symporter | 1.33975829 | 0.00133882 |
| Soltu.DM.11G010260 | Leucine-rich repeat protein kinase family protein | -1.83276091 | 0.01016746 |
| Soltu.DM.11G010770 | Pathogenesis-related thaumatin superfamily protein | 1.65601936 | 0.04182213 |
| Soltu.DM.11G015080 | no exine formation | -1.73645338 | 0.00844825 |
| Soltu.DM.11G016270 | conserved hypothetical protein | 3.40807485 | 0.03688714 |
| Soltu.DM.11G016790 | osmotin | -2.41739386 | 0.03439799 |
| Soltu.DM.11G017460 | Peptidase M28 family protein | -5.51788902 | 0.03951531 |
| Soltu.DM.11G017650 | FAD-linked oxidases family protein | 1.85007436 | 0.02443491 |
| Soltu.DM.11G020040 | MATE efflux family protein | 2.08569603 | 0.01542772 |
| Soltu.DM.11G021030 | Eukaryotic aspartyl protease family protein | -1.05128501 | 0.03727044 |
| Soltu.DM.11G021070 | magnesium (Mg) transporter | 1.93842513 | 0.02047146 |
| Soltu.DM.11G021210 | cellulose synthase-like A02 | 1.6697238 | 0.01728082 |
| Soltu.DM.11G021230 | histone deacetylase 2C | -1.03213604 | 0.03676839 |
| Soltu.DM.11G021410 | hypothetical protein | -1.50147164 | 0.015911 |
| Soltu.DM.11G022120 | F-box family protein | -2.5681976 | 0.00808209 |
| Soltu.DM.11G022280 | Late embryogenesis abundant (LEA) hydroxyproline-rich glycoprotein family | -1.40780716 | 0.01574716 |
| Soltu.DM.11G022780 | Kiwellin | -2.22293748 | 0.01740357 |
| Soltu.DM.11G022830 | alpha carbonic anhydrase | -3.60488461 | 0.0278615 |
| Soltu.DM.11G022840 | alpha carbonic anhydrase | -3.37879603 | 0.04097435 |
| Soltu.DM.11G023870 | Nucleotide-diphospho-sugar transferases superfamily protein | 1.02105371 | 0.00323793 |
| Soltu.DM.11G026250 | P-loop containing nucleoside triphosphate hydrolases superfamily protein | 1.28525522 | 0.00729596 |
| Soltu.DM.12G000120 | receptor like protein | -1.28731511 | 0.03158056 |
| Soltu.DM.12G001210 | Protein of unknown function (DUF604) | -2.32906231 | 0.03933394 |
| Soltu.DM.12G001430 | conserved hypothetical protein | -1.30103266 | 0.03428918 |
| Soltu.DM.12G002400 | Late blight resistance protein R1 domain containing protein | 3.70454862 | 0.04282769 |
| Soltu.DM.12G002640 | UDP-D-glucuronate 4-epimerase | 2.05650607 | 0.03951049 |
| Soltu.DM.12G004730 | Domain of unknown function (DUF23) | -1.68565682 | 0.03630269 |
| Soltu.DM.12G004770 | Protein of unknown function (DUF594) | -1.53169837 | 0.01559331 |
| Soltu.DM.12G005750 | alpha/beta-Hydrolases superfamily protein | -5.33585342 | 0.00418157 |
| Soltu.DM.12G006150 | Peroxisomal membrane 22 kDa (Mpv17/PMP22) family protein | -1.63442612 | 0.00216812 |
| Soltu.DM.12G008740 | RING/U-box superfamily protein | -2.80963895 | 0.00890726 |
| Soltu.DM.12G009270 | Protein kinase family protein with leucine-rich repeat domain | -5.17178579 | 0.01239155 |
| Soltu.DM.12G009490 | Cytochrome P450 superfamily protein | -1.73113634 | 0.03154933 |
| Soltu.DM.12G009550 | Cytochrome P450 superfamily protein | -1.82826302 | 0.01815954 |
| Soltu.DM.12G010360 | tonoplast intrinsic protein | 3.20200825 | 0.00254102 |
| Soltu.DM.12G010700 | sulfate transporter 3;4 | 1.64982218 | 0.03860876 |
| Soltu.DM.12G011100 | early nodulin-like protein | 3.40045313 | 0.01266102 |
| Soltu.DM.12G011950 | Protein kinase superfamily protein | -1.11082352 | 0.02101955 |
| Soltu.DM.12G012180 | Protein of unknown function (DUF1666) | 1.64051604 | 0.00596631 |
| Soltu.DM.12G019510 | transporter associated with antigen processing protein | -1.34465824 | 0.03489124 |
| Soltu.DM.12G019530 | serine carboxypeptidase-like | 2.86003906 | 0.04133589 |
| Soltu.DM.12G019570 | formin homology | 2.08262545 | 0.01436415 |
| Soltu.DM.12G020650 | Carbohydrate-binding X8 domain superfamily protein | 4.09624334 | 0.03520492 |
| Soltu.DM.12G021100 | cellulose synthase like E1 | 1.30037799 | 0.03800739 |
| Soltu.DM.12G023100 | Protein of unknown function (DUF604) | 2.44913942 | 0.02568919 |
| Soltu.DM.12G023590 | FASCICLIN-like arabinogalactan | -2.662856 | 0.01933992 |
| Soltu.DM.12G025140 | xyloglucan endotransglucosylase/hydrolase | -2.85003587 | 0.00822296 |
| Soltu.DM.12G025460 | Protein kinase family protein with leucine-rich repeat domain | -1.60140085 | 0.03572455 |
| Soltu.DM.12G027460 | hypothetical protein | 2.28886094 | 0.029131 |
| Soltu.DM.12G027820 | Major facilitator superfamily protein | -1.52711235 | 0.04231668 |
| Soltu.DM.12G028090 | RING membrane-anchor | -1.56111922 | 0.02053092 |
| Soltu.DM.12G028690 | Nucleotide/sugar transporter family protein | -1.48695295 | 0.0163948 |
| Intrinsic component of membrane | | | |
| Soltu.DM.01G000220 | Cytochrome b561/ferric reductase transmembrane protein family | -1.11485095 | 0.02164664 |
| Soltu.DM.01G005630 | signal peptide peptidase | -1.10695955 | 0.04993759 |
| Soltu.DM.01G006240 | HSP20-like chaperones superfamily protein | 1.42922142 | 0.04181753 |
| Soltu.DM.01G007020 | receptor like protein | 1.22423421 | 0.04814904 |
| Soltu.DM.01G010340 | plastid-encoded CLP P | -2.85762188 | 0.02689996 |
| Soltu.DM.01G014450 | Got1/Sft2-like vescicle transport protein family | -1.18797812 | 0.00613355 |
| Soltu.DM.01G021920 | vesicle-associated membrane protein | -1.31310073 | 0.01804099 |
| Soltu.DM.01G022390 | Protein kinase superfamily protein | 4.47692598 | 0.00736973 |
| Soltu.DM.01G023870 | REF4-related | 1.04333869 | 0.04548647 |
| Soltu.DM.01G025960 | Auxin efflux carrier family protein | 4.50045796 | 0.01116635 |
| Soltu.DM.01G026030 | Leucine-rich repeat protein kinase family protein | 1.42819026 | 0.00135448 |
| Soltu.DM.01G028050 | O-fucosyltransferase family protein | -1.4538247 | 0.02685231 |
| Soltu.DM.01G030040 | metacaspase | -1.37901942 | 0.00811813 |
| Soltu.DM.01G030400 | CAMV movement protein interacting protein | -1.91049245 | 0.0006159 |
| Soltu.DM.01G031110 | Cotton fibre expressed protein domain containing protein | 1.49692608 | 0.02114228 |
| Soltu.DM.01G032570 | UDP-N-acetylglucosamine (UAA) transporter family | -1.21879721 | 0.02343367 |
| Soltu.DM.01G033920 | transmembrane protein-related | -1.0536456 | 0.01217867 |
| Soltu.DM.01G036060 | Major facilitator superfamily protein | 2.11517544 | 0.03740651 |
| Soltu.DM.01G036140 | HSP20-like chaperones superfamily protein | 2.77459472 | 0.04637185 |
| Soltu.DM.01G037800 | receptor like protein | -1.14658944 | 0.04457812 |
| Soltu.DM.01G039100 | soybean gene regulated by cold-2 | -1.31082399 | 0.03879511 |
| Soltu.DM.01G040680 | GDSL-like Lipase/Acylhydrolase superfamily protein | 1.44223732 | 0.01361095 |
| Soltu.DM.01G041480 | Leucine-rich repeat protein kinase family protein | -1.13202986 | 0.04525049 |
| Soltu.DM.01G041510 | Leucine-rich repeat protein kinase family protein | 3.27809854 | 0.04945643 |
| Soltu.DM.01G042270 | DNA-directed RNA polymerase III subunit Rpc31 domain containing protein | -2.49923374 | 0.02604244 |
| Soltu.DM.01G042510 | Protein of unknown function (DUF679) | -2.04632868 | 0.01340525 |
| Soltu.DM.01G045470 | F-box family protein with a domain of unknown function (DUF295) | -2.16221483 | 0.0168418 |
| Soltu.DM.01G045660 | fatty acid amide hydrolase | 1.70362438 | 0.01624358 |
| Soltu.DM.01G047000 | Leucine-rich repeat protein kinase family protein | 1.56788961 | 0.00596107 |
| Soltu.DM.01G047740 | cytochrome P450, family 707, subfamily A, polypeptide | -2.20871192 | 0.02498343 |
| Soltu.DM.01G047850 | conserved hypothetical protein | 1.52458985 | 0.03544061 |
| Soltu.DM.01G049560 | Major facilitator superfamily protein | -1.15961879 | 0.01414419 |
| Soltu.DM.01G050940 | Major facilitator superfamily protein | -3.2169536 | 0.00148178 |
| Soltu.DM.01G051250 | PAM domain (PCI/PINT associated module) protein | -2.567064 | 0.02881064 |
| Soltu.DM.01G051570 | Transmembrane amino acid transporter family protein | -1.35890951 | 0.0425949 |
| Soltu.DM.02G001220 | S-locus lectin protein kinase family protein | -1.44794196 | 0.0160473 |
| Soltu.DM.02G001580 | cytochrome P450, family 86, subfamily B, polypeptide | -2.99664368 | 0.02055293 |
| Soltu.DM.02G003520 | conserved hypothetical protein | -1.09169548 | 0.01247621 |
| Soltu.DM.02G004170 | B-cell receptor-associated 31-like | -2.22012139 | 0.02933466 |
| Soltu.DM.02G004650 | Late embryogenesis abundant (LEA) hydroxyproline-rich glycoprotein family | -2.00541997 | 0.01137635 |
| Soltu.DM.02G006070 | BURP domain-containing protein | -3.85617172 | 0.00825444 |
| Soltu.DM.02G006910 | Target SNARE coiled-coil domain protein | -5.13281412 | 0.04669588 |
| Soltu.DM.02G007060 | abscisic acid (aba)-deficient | 1.14029725 | 0.04961956 |
| Soltu.DM.02G007100 | Leucine-rich repeat protein kinase family protein | -4.14690072 | 0.0449072 |
| Soltu.DM.02G010360 | RING/U-box superfamily protein | -3.64448053 | 0.01654176 |
| Soltu.DM.02G010690 | HAUS augmin-like complex subunit 6 N-terminus domain containing protein | 1.02411567 | 0.01009957 |
| Soltu.DM.02G010760 | Leucine-rich repeat protein kinase family protein | 1.52733269 | 0.01028899 |
| Soltu.DM.02G011200 | RING/U-box superfamily protein | 1.05174788 | 0.03376342 |
| Soltu.DM.02G011480 | Ankyrin repeat family protein | 4.3820096 | 0.01694404 |
| Soltu.DM.02G011540 | Cysteine/Histidine-rich C1 domain family protein | -1.23981878 | 0.02450651 |
| Soltu.DM.02G012460 | photosystem I subunit F | 1.95405379 | 0.0104997 |
| Soltu.DM.02G014910 | Leucine-rich repeat transmembrane protein kinase | -6.00542911 | 0.01650919 |
| Soltu.DM.02G015840 | Leucine-rich repeat protein kinase family protein | 2.14636223 | 0.03782257 |
| Soltu.DM.02G016560 | glutamate receptor 2.9 | -3.73275192 | 0.00422831 |
| Soltu.DM.02G016590 | glutamate receptor 2.9 | -4.91622206 | 0.00391129 |
| Soltu.DM.02G017590 | Concanavalin A-like lectin protein kinase family protein | -1.58402873 | 0.00509532 |
| Soltu.DM.02G018000 | alpha 1,4-glycosyltransferase family protein | 1.00012976 | 0.02406568 |
| Soltu.DM.02G018040 | Protein of unknown function (DUF1218) | -1.18394997 | 0.04148537 |
| Soltu.DM.02G018160 | STRUBBELIG-receptor family | 1.6939196 | 0.04378666 |
| Soltu.DM.02G018870 | Major facilitator superfamily protein | 2.03006473 | 0.02355864 |
| Soltu.DM.02G019090 | protein serine/threonine kinases;protein kinases;ATP binding;sugar binding;kinases;carbohydrate binding | -1.30336382 | 0.02304744 |
| Soltu.DM.02G019120 | S-locus lectin protein kinase family protein | -2.87128399 | 0.00115305 |
| Soltu.DM.02G019150 | S-locus lectin protein kinase family protein | -1.56471484 | 0.00909483 |
| Soltu.DM.02G023370 | early-responsive to dehydration stress protein (ERD4) | -1.11904815 | 0.03024028 |
| Soltu.DM.02G023400 | Eukaryotic aspartyl protease family protein | -1.62795415 | 0.01106241 |
| Soltu.DM.02G023580 | ascorbate peroxidase | 1.49085967 | 0.01881879 |
| Soltu.DM.02G024410 | Protein kinase family protein with leucine-rich repeat domain | 1.57694784 | 0.02961793 |
| Soltu.DM.02G024730 | P-loop containing nucleoside triphosphate hydrolases superfamily protein | -1.37711282 | 0.01819623 |
| Soltu.DM.02G026160 | Protein kinase superfamily protein | -1.57576023 | 0.0224153 |
| Soltu.DM.02G026570 | conserved hypothetical protein | -2.35513996 | 0.00480052 |
| Soltu.DM.02G028050 | natural resistance-associated macrophage protein | 1.14523618 | 0.02330156 |
| Soltu.DM.02G029010 | conserved hypothetical protein | -3.43918743 | 0.04621445 |
| Soltu.DM.02G029930 | PQ-loop repeat family protein / transmembrane family protein | 1.39461301 | 0.03609191 |
| Soltu.DM.02G030250 | NSP-interacting kinase | 1.78852085 | 0.01659677 |
| Soltu.DM.02G030670 | COBRA-like extracellular glycosyl-phosphatidyl inositol-anchored protein family | 1.02316234 | 0.01022626 |
| Soltu.DM.02G031640 | H(+)-ATPase | -2.29594394 | 0.00507474 |
| Soltu.DM.02G032290 | Putative membrane lipoprotein | 3.16184219 | 0.01267463 |
| Soltu.DM.02G032400 | B12D protein | -1.54583308 | 0.02776949 |
| Soltu.DM.02G033170 | cysteine-rich RLK (RECEPTOR-like protein kinase) | -1.67529944 | 0.02090847 |
| Soltu.DM.02G034260 | Major facilitator superfamily protein | -1.47896568 | 0.02102236 |
| Soltu.DM.03G001910 | HAESA-like | 3.1226572 | 0.03713183 |
| Soltu.DM.03G002030 | LEUNIG_homolog | -2.35984589 | 0.0344959 |
| Soltu.DM.03G002340 | Protein of unknown function (DUF761) | -2.18256475 | 0.01451758 |
| Soltu.DM.03G003460 | NEP-interacting protein | 1.08018171 | 0.02114186 |
| Soltu.DM.03G003760 | Leucine-rich receptor-like protein kinase family protein | 2.81361618 | 0.00080919 |
| Soltu.DM.03G005050 | Leucine-rich receptor-like protein kinase family protein | 1.99158202 | 0.01972997 |
| Soltu.DM.03G009280 | Leucine-rich repeat receptor-like protein kinase family protein | 1.59149287 | 0.02958573 |
| Soltu.DM.03G014330 | ABC transporter family protein | 1.16604394 | 0.04971796 |
| Soltu.DM.03G019480 | conserved hypothetical protein | -1.00619137 | 0.0234547 |
| Soltu.DM.03G019620 | phosphate transporter 3;1 | -1.87949127 | 0.04296169 |
| Soltu.DM.03G021550 | conserved hypothetical protein | 3.18471177 | 0.04229549 |
| Soltu.DM.03G021720 | Lung seven transmembrane receptor family protein | -1.04372429 | 0.03418749 |
| Soltu.DM.03G022660 | RING/U-box superfamily protein | -2.66531623 | 0.00292236 |
| Soltu.DM.03G022670 | Protein kinase superfamily protein | -1.35776174 | 0.01884466 |
| Soltu.DM.03G022700 | hypothetical protein | -1.41259975 | 0.01855298 |
| Soltu.DM.03G025010 | S-adenosyl-L-methionine-dependent methyltransferases superfamily protein | 2.53220549 | 0.01263805 |
| Soltu.DM.03G025590 | cytochrome P450, family 71, subfamily A, polypeptide | -1.89246577 | 0.03561065 |
| Soltu.DM.03G026850 | hypothetical protein | -1.54385021 | 0.02360074 |
| Soltu.DM.03G027360 | Leucine-rich repeat protein kinase family protein | 1.80811855 | 0.03093777 |
| Soltu.DM.03G028670 | LAG1 longevity assurance homolog | -1.592237 | 0.02970346 |
| Soltu.DM.03G029090 | cytochrome P450, family 78, subfamily A, polypeptide | 3.06467609 | 0.01332973 |
| Soltu.DM.03G029730 | Leucine-rich repeat protein kinase family protein | -1.20283926 | 0.0422184 |
| Soltu.DM.03G030660 | Embryo-specific protein 3, (ATS3) | -1.17572793 | 0.0263981 |
| Soltu.DM.03G031200 | NOD26-like intrinsic protein 6;1 | 2.59887586 | 0.00032522 |
| Soltu.DM.03G032300 | calnexin | -1.08621297 | 0.04412523 |
| Soltu.DM.03G033110 | Auxin efflux carrier family protein | 1.01356807 | 0.03691859 |
| Soltu.DM.03G033670 | Protein kinase superfamily protein | -1.79742148 | 0.02338297 |
| Soltu.DM.03G033780 | Peptidase M50B-like domain containing protein | 4.86777301 | 0.00185007 |
| Soltu.DM.03G035440 | conserved hypothetical protein | -1.02155938 | 0.01254284 |
| Soltu.DM.03G037010 | Lung seven transmembrane receptor family protein | 1.42336795 | 0.03271196 |
| Soltu.DM.04G000050 | Matrixin family protein | -1.92559072 | 0.02794036 |
| Soltu.DM.04G002260 | Protein kinase superfamily protein | -1.02883841 | 0.03390872 |
| Soltu.DM.04G002550 | conserved hypothetical protein | -1.80686261 | 0.02442407 |
| Soltu.DM.10G027550 | beta-1,3-glucanase | -2.92339235 | 0.00888368 |
| Soltu.DM.11G000800 | Cupredoxin superfamily protein | 1.63688598 | 0.01675106 |
| Soltu.DM.12G011650 | O-Glycosyl hydrolases family 17 protein | 2.2085718 | 0.01579723 |
| Soltu.DM.S002220 | Glycosyl hydrolase superfamily protein | -5.75279263 | 0.00551471 |
| Intrinsic component of plasma membrane | | | |
| Soltu.DM.01G005200 | beta-1,3-glucanase | -4.59338232 | 0.0199515 |
| Soltu.DM.01G008280 | plasmodesmata callose-binding protein | 1.88906391 | 0.03238192 |
| Cell wall | | | |
| Soltu.DM.01G029240 | xyloglucan endotransglucosylase/hydrolase | -1.24521153 | 0.02579054 |
| Soltu.DM.01G036420 | pathogenesis-related | -2.37143842 | 0.00434617 |
| Soltu.DM.01G040800 | Peroxidase superfamily protein | -5.30669943 | 0.01095948 |
| Soltu.DM.01G041270 | germin-like protein | -1.72886327 | 0.01579489 |
| Soltu.DM.01G041740 | germin-like protein | -2.27005764 | 0.02828055 |
| Soltu.DM.01G041810 | germin-like protein | -1.76419363 | 0.03871896 |
| Soltu.DM.01G041830 | RmlC-like cupins superfamily protein | -2.08084554 | 0.00519518 |
| Soltu.DM.02G020130 | Peroxidase superfamily protein | 1.62108255 | 0.00585912 |
| Soltu.DM.02G020270 | SKU5 similar | 2.68360445 | 0.04324736 |
| Soltu.DM.02G029430 | SKU5 similar | 1.10090717 | 0.01290468 |
| Soltu.DM.02G031610 | expansin A10 | 1.6929925 | 0.0254793 |
| Soltu.DM.03G018570 | kunitz trypsin inhibitor | 4.08082087 | 0.02091395 |
| Soltu.DM.03G027490 | RmlC-like cupins superfamily protein | -3.90332386 | 0.01423824 |
| Soltu.DM.03G030010 | expansin A15 | 2.01465324 | 0.00658978 |
| Soltu.DM.04G037290 | SKU5 similar | 2.13803548 | 0.00670126 |
| Soltu.DM.05G008450 | polygalacturonase | 1.83061191 | 0.02541325 |
| Soltu.DM.05G008480 | polygalacturonase | 2.13885215 | 0.00187852 |
| Soltu.DM.05G008500 | polygalacturonase | 2.04658745 | 0.00118652 |
| Soltu.DM.06G031540 | expansin A6 | 1.49999472 | 0.0437076 |
| Soltu.DM.07G002190 | Xyloglucan endotransglucosylase/hydrolase family protein | -2.02591436 | 0.03218808 |
| Soltu.DM.07G004760 | subtilisin-like serine protease | 1.45561769 | 0.02039504 |
| Soltu.DM.07G012760 | Subtilase family protein | 1.12500915 | 0.02416125 |
| Soltu.DM.07G022000 | xyloglucan endotransglucosylase/hydrolase | -1.87924181 | 0.00840713 |
| Soltu.DM.07G022400 | xyloglucan endotransglucosylase/hydrolase | -1.3243544 | 0.02850132 |
| Soltu.DM.09G002110 | Barwin-like endoglucanases superfamily protein | 2.6768259 | 0.00180396 |
| Soltu.DM.09G023660 | Plant invertase/pectin methylesterase inhibitor superfamily | 1.9498324 | 0.03672531 |
| Soltu.DM.10G017970 | basic chitinase | -4.05891015 | 0.02349777 |
| Soltu.DM.11G008230 | Plant invertase/pectin methylesterase inhibitor superfamily | 1.49982498 | 0.01719116 |
| Soltu.DM.12G004990 | SKU5 similar | 2.09170786 | 0.0032285 |
| Cell wall organization or biogenesis | | | |
| Soltu.DM.01G031690 | Plant invertase/pectin methylesterase inhibitor superfamily | -4.55599855 | 0.0274712 |
| Soltu.DM.01G038550 | Cyclin family protein | 1.2852225 | 0.02894798 |
| Soltu.DM.01G040810 | Peroxidase superfamily protein | -4.88858414 | 0.00174268 |
| Soltu.DM.02G013980 | Leucine-rich receptor-like protein kinase family protein | -1.96981943 | 0.00560827 |
| Soltu.DM.02G019780 | xyloglucan endotransglucosylase/hydrolase | 2.24120852 | 0.02512935 |
| Soltu.DM.02G024320 | Pectin lyase-like superfamily protein | 5.33350962 | 0.01747249 |
| Soltu.DM.02G030620 | reversibly glycosylated polypeptide | 2.65846928 | 0.03308911 |
| Soltu.DM.03G013870 | Nucleotide-diphospho-sugar transferase family protein | -2.7899122 | 0.00107571 |
| Soltu.DM.04G000790 | sec7 domain-containing protein | 1.13401241 | 0.03273164 |
| Soltu.DM.12G002200 | Pollen Ole e 1 allergen and extensin family protein | -1.44641374 | 0.04288822 |
| Soltu.DM.12G002220 | Pollen Ole e 1 allergen and extensin family protein | -1.50707888 | 0.04351732 |
| Soltu.DM.12G002240 | Pollen Ole e 1 allergen and extensin family protein | -1.70392865 | 0.04433017 |
| Soltu.DM.12G002250 | Pollen Ole e 1 allergen and extensin family protein | -1.60972737 | 0.03676882 |
