# Supplementary material for: The Induction of Disease Resistance by Scopolamine and the Application of Datura Extract Against Potato (Solanum tuberosum L.) Late Blight
Source: Int J Mol Sci. 2024 Dec 15;25(24):13442. doi: 10.3390/ijms252413442 (PMC11676833; doi:10.3390/ijms252413442)
Supplement: Supplementary file 1 [file ijms-25-13442-s001.zip › Supplementary Table 8.docx]

**Supplementary Table 8 DEGs related to plant-pathogen interaction**

| gene name | gene description | log_2_FC(S/C) | *P* value |
| --- | --- | --- | --- |
| PAMP-triggered immunity，PTI |  |  |  |
| Soltu.DM.01G044530 | metacaspase | -7.416925389 | 7.45E-06 |
| Soltu.DM.02G016980 | homeobox | -6.546920534 | 1.29E-05 |
| Soltu.DM.03G013350 | WRKY DNA-binding protein | -5.813735288 | 8.42E-05 |
| Soltu.DM.09G011140 | WRKY DNA-binding protein | -5.732743763 | 0.000354015 |
| Soltu.DM.04G034390 | Serine protease inhibitor (SERPIN) family protein | -5.517889016 | 0.000393521 |
| Soltu.DM.07G006060 | Transcriptional factor B3 family protein / auxin-responsive factor AUX/IAA-related | -5.366981821 | 0.000543518 |
| Soltu.DM.08G015900 | WRKY DNA-binding protein | -5.346458175 | 0.000544445 |
| Soltu.DM.02G003130 | Homeobox-leucine zipper family protein / lipid-binding START domain-containing protein | -5.075121252 | 0.000582659 |
| Soltu.DM.10G028420 | squalene synthase | -4.384077209 | 0.000654687 |
| Soltu.DM.03G023740 | Kunitz family trypsin and protease inhibitor protein | -3.674476138 | 0.00108946 |
| Soltu.DM.05G019150 | auxin response factor | -3.581681631 | 0.001177341 |
| Soltu.DM.07G007140 | AINTEGUMENTA-like | -3.514670351 | 0.001207479 |
| Soltu.DM.05G011490 | P-loop containing nucleoside triphosphate hydrolases superfamily protein | -3.408461152 | 0.001278139 |
| Soltu.DM.04G033540 | basic helix-loop-helix (bHLH) DNA-binding superfamily protein | -3.372942727 | 0.001304354 |
| Soltu.DM.02G010100 | hypothetical protein | -3.35713397 | 0.001391727 |
| Soltu.DM.02G027660 | GRAS family transcription factor | -3.282484331 | 0.001392054 |
| Soltu.DM.01G043490 | GATA transcription factor | -3.250920629 | 0.00142244 |
| Soltu.DM.03G033780 | Peptidase M50B-like domain containing protein | -3.096326884 | 0.00185007 |
| Soltu.DM.04G006770 | phosphoenolpyruvate carboxylase kinase | -3.035114226 | 0.002043275 |
| Soltu.DM.05G023310 | myb domain protein | -2.999244571 | 0.002072024 |
| Soltu.DM.06G032070 | Homeodomain-like transcriptional regulator | -2.99664368 | 0.002250795 |
| Soltu.DM.08G014820 | Homeobox-leucine zipper family protein / lipid-binding START domain-containing protein | -2.884204966 | 0.002301934 |
| Soltu.DM.03G019980 | NAC domain containing protein | -2.857621884 | 0.002459836 |
| Soltu.DM.02G010090 | hypothetical protein | -2.780876319 | 0.002461257 |
| Soltu.DM.03G016610 | peptidoglycan-binding LysM domain-containing protein | -2.775794105 | 0.002487247 |
| Soltu.DM.08G000230 | Subtilase family protein | -2.770579061 | 0.002580509 |
| Soltu.DM.03G034300 | TCP family transcription factor | -2.758658163 | 0.002611221 |
| Soltu.DM.08G027050 | GRAS family transcription factor | -2.711388268 | 0.002713578 |
| Soltu.DM.12G007600 | Integrase-type DNA-binding superfamily protein | -2.708442087 | 0.002886724 |
| Soltu.DM.05G003270 | myb domain protein | -2.696551932 | 0.003168369 |
| Soltu.DM.12G029300 | P-loop containing nucleoside triphosphate hydrolases superfamily protein | -2.690054206 | 0.003267052 |
| Soltu.DM.02G026320 | Potato type II proteinase inhibitor family domain containing protein | -2.588919831 | 0.003446463 |
| Soltu.DM.01G024100 | Eukaryotic aspartyl protease family protein | -2.575036547 | 0.003549541 |
| Soltu.DM.04G034380 | Serine protease inhibitor (SERPIN) family protein | -2.558140834 | 0.003603787 |
| Soltu.DM.03G030960 | WRKY DNA-binding protein | -2.526461473 | 0.003741125 |
| Soltu.DM.02G016650 | Integrase-type DNA-binding superfamily protein | -2.518994 | 0.003802812 |
| Soltu.DM.10G000040 | pseudo-response regulator | -2.499233736 | 0.004053628 |
| Soltu.DM.05G022450 | DRE-binding protein 2A | -2.492112936 | 0.004082212 |
| Soltu.DM.04G031480 | oligopeptide transporter | -2.476938525 | 0.004309929 |
| Soltu.DM.02G017390 | TARGET OF MONOPTEROS | -2.432823395 | 0.004682809 |
| Soltu.DM.06G011430 | Basic-leucine zipper (bZIP) transcription factor family protein | -2.410293852 | 0.004692911 |
| Soltu.DM.02G015140 | heat shock transcription factor A4A | -2.395188955 | 0.004748112 |
| Soltu.DM.03G037200 | GRAS family transcription factor | -2.359845892 | 0.004920364 |
| Soltu.DM.08G004690 | WRKY family transcription factor | -2.286093646 | 0.005184803 |
| Soltu.DM.10G030060 | cytochrome P450, family 98, subfamily A, polypeptide | -2.275640526 | 0.005311094 |
| Soltu.DM.05G027130 | Seed dormancy control domain containing protein | -2.27341813 | 0.005363902 |
| Soltu.DM.04G035460 | RAD-like | -2.252656857 | 0.005425309 |
| Soltu.DM.01G032120 | AT-hook motif nuclear-localized protein | -2.250617629 | 0.005586544 |
| Soltu.DM.10G030010 | cytochrome P450, family 98, subfamily A, polypeptide | -2.215527734 | 0.006080113 |
| Soltu.DM.08G026790 | phospholipase D beta | -2.208711919 | 0.006086762 |
| Soltu.DM.06G031590 | SCARECROW-like | -2.149573049 | 0.006309786 |
| Soltu.DM.02G014620 | subtilase family protein | -2.110252559 | 0.00656118 |
| Soltu.DM.02G023600 | Tetratricopeptide repeat (TPR)-like superfamily protein | -2.108051684 | 0.006793458 |
| Soltu.DM.12G022230 | GRAS family transcription factor | -2.042179579 | 0.006831792 |
| Soltu.DM.11G000190 | oligopeptide transporter | -2.036506453 | 0.006978845 |
| Soltu.DM.11G022180 | Polynucleotidyl transferase, ribonuclease H-like superfamily protein | -2.014887119 | 0.00716206 |
| Soltu.DM.12G020490 | hypothetical protein | -1.976155923 | 0.007215285 |
| Soltu.DM.09G000420 | polygalacturonase inhibiting protein | -1.972811198 | 0.007310605 |
| Soltu.DM.07G001290 | Tetratricopeptide repeat (TPR)-like superfamily protein | -1.948581964 | 0.007363754 |
| Soltu.DM.02G017750 | heat shock transcription factor B2A | -1.944658864 | 0.00739508 |
| Soltu.DM.03G035640 | Homeobox-leucine zipper family protein / lipid-binding START domain-containing protein | -1.932033797 | 0.007418696 |
| Soltu.DM.10G015470 | cytochrome P450, family 72, subfamily A, polypeptide | -1.931603816 | 0.007430081 |
| Soltu.DM.06G027050 | Tetratricopeptide repeat (TPR)-like superfamily protein | -1.925590718 | 0.007573152 |
| Soltu.DM.02G020420 | aspartic proteinase A1 | -1.907796302 | 0.007609242 |
| Soltu.DM.01G030040 | metacaspase | -1.902520747 | 0.008118129 |
| Soltu.DM.03G026770 | myb domain protein | -1.892465774 | 0.008272209 |
| Soltu.DM.05G020140 | Tetratricopeptide repeat (TPR)-like superfamily protein | -1.891382591 | 0.008401638 |
| Soltu.DM.02G023310 | conserved hypothetical protein | -1.875473437 | 0.008417153 |
| Soltu.DM.04G005640 | TCP family transcription factor | -1.865424755 | 0.008939693 |
| Soltu.DM.04G030360 | basic helix-loop-helix (bHLH) DNA-binding superfamily protein | -1.865255227 | 0.009452243 |
| Soltu.DM.06G030230 | serine carboxypeptidase-like | -1.865069528 | 0.009538908 |
| Soltu.DM.08G029980 | YELLOW STRIPE like | -1.860088458 | 0.009683262 |
| Soltu.DM.08G000970 | ethylene responsive element binding factor | -1.849168921 | 0.009687079 |
| Soltu.DM.01G026640 | AGAMOUS-like | -1.826814894 | 0.009890604 |
| Soltu.DM.02G020620 | KNOX/ELK homeobox transcription factor | -1.778263871 | 0.010187194 |
| Soltu.DM.10G021890 | WRKY DNA-binding protein | -1.768838549 | 0.010429945 |
| Soltu.DM.02G023400 | Eukaryotic aspartyl protease family protein | -1.748539971 | 0.011062406 |
| Soltu.DM.11G009740 | NAC (No Apical Meristem) domain transcriptional regulator superfamily protein | -1.740079067 | 0.011295817 |
| Soltu.DM.02G016660 | ethylene responsive element binding factor | -1.728978205 | 0.011313563 |
| Soltu.DM.03G018650 | Trypsin and protease inhibitor domain containing protein | -1.695667836 | 0.012186043 |
| Soltu.DM.04G020620 | hypothetical protein | -1.692211808 | 0.012354767 |
| Soltu.DM.02G028120 | cytochrome P450, family 81, subfamily D, polypeptide | -1.688914644 | 0.012514359 |
| Soltu.DM.04G006520 | pentatricopeptide (PPR) repeat-containing protein | -1.679194736 | 0.01251544 |
| Soltu.DM.01G017170 | CTP synthase family protein | -1.675805402 | 0.012529913 |
| Soltu.DM.06G000110 | Tetratricopeptide repeat (TPR)-like superfamily protein | -1.672767864 | 0.012643448 |
| Soltu.DM.04G001370 | basic helix-loop-helix (bHLH) DNA-binding superfamily protein | -1.639164071 | 0.013092076 |
| Soltu.DM.11G021880 | Calcium-binding EF-hand family protein | -1.62795415 | 0.013243181 |
| Soltu.DM.03G029090 | cytochrome P450, family 78, subfamily A, polypeptide | -1.625695488 | 0.013329731 |
| Soltu.DM.07G028450 | NAC domain containing protein | -1.614917961 | 0.01376108 |
| Soltu.DM.05G016500 | Eukaryotic aspartyl protease family protein | -1.593832648 | 0.01397466 |
| Soltu.DM.10G025490 | syntaxin of plants | -1.54729179 | 0.014327174 |
| Soltu.DM.10G017860 | NAC domain containing protein | -1.506634666 | 0.014469098 |
| Soltu.DM.03G018430 | Kunitz family trypsin and protease inhibitor protein | -1.500964624 | 0.014558374 |
| Soltu.DM.03G004580 | Peptidase M50B-like domain containing protein | -1.49760564 | 0.015144047 |
| Soltu.DM.10G021990 | myb-related protein | -1.454463813 | 0.015948342 |
| Soltu.DM.08G024170 | ethylene responsive element binding factor | -1.446329763 | 0.016267282 |
| Soltu.DM.07G003640 | Tetratricopeptide repeat (TPR)-like superfamily protein | -1.420401211 | 0.016379038 |
| Soltu.DM.01G037560 | Esterase/lipase/thioesterase family protein | -1.410225724 | 0.016694258 |
| Soltu.DM.07G001200 | Class I glutamine amidotransferase-like superfamily protein | -1.405592877 | 0.016778167 |
| Soltu.DM.09G019050 | Tetratricopeptide repeat (TPR)-like superfamily protein | -1.391828089 | 0.01696952 |
| Soltu.DM.02G029910 | protein kinase family protein / peptidoglycan-binding LysM domain-containing protein | -1.387412493 | 0.016984628 |
| Soltu.DM.06G006740 | serine carboxypeptidase-like | -1.381013742 | 0.017155994 |
| Soltu.DM.07G004210 | CCT motif family protein | -1.379019421 | 0.017364914 |
| Soltu.DM.07G021360 | cytochrome P450, family 72, subfamily A, polypeptide | -1.359885731 | 0.017554069 |
| Soltu.DM.03G026030 | histone deacetylase | -1.357340009 | 0.017591132 |
| Soltu.DM.05G023230 | Protein kinase superfamily protein | -1.350660335 | 0.017807519 |
| Soltu.DM.07G009920 | alpha/beta-Hydrolases superfamily protein | -1.349848417 | 0.018179799 |
| Soltu.DM.08G024160 | ethylene responsive element binding factor | -1.348603921 | 0.018200339 |
| Soltu.DM.06G026730 | Protein of Unknown Function (DUF239) | -1.346853516 | 0.018313198 |
| Soltu.DM.05G021130 | AINTEGUMENTA-like | -1.31576238 | 0.018566364 |
| Soltu.DM.01G042780 | AGAMOUS-like | -1.305699326 | 0.018736754 |
| Soltu.DM.07G000850 | MIF4G domain-containing protein / MA3 domain-containing protein | -1.305265151 | 0.019129038 |
| Soltu.DM.10G024710 | cytochrome P450, family 76, subfamily G, polypeptide | -1.296209089 | 0.019485406 |
| Soltu.DM.07G020250 | Homeodomain-like transcriptional regulator | -1.293883519 | 0.019790143 |
| Soltu.DM.10G027770 | IAA-leucine resistant (ILR)-like gene | -1.285010243 | 0.019815931 |
| Soltu.DM.03G016270 | subtilase 1.3 | -1.256464922 | 0.020222704 |
| Soltu.DM.04G035810 | basic helix-loop-helix (bHLH) DNA-binding superfamily protein | -1.237907674 | 0.020241494 |
| Soltu.DM.07G004760 | subtilisin-like serine protease | -1.205118126 | 0.020395035 |
| Soltu.DM.03G017800 | cytochrome P450, family 715, subfamily A, polypeptide | -1.193734444 | 0.020496653 |
| Soltu.DM.03G032450 | Integrase-type DNA-binding superfamily protein | -1.165630333 | 0.020545248 |
| Soltu.DM.02G001580 | cytochrome P450, family 86, subfamily B, polypeptide | -1.164154906 | 0.020552925 |
| Soltu.DM.02G034670 | DEAD/DEAH box helicase, putative | -1.155950891 | 0.020824681 |
| Soltu.DM.04G027260 | ABA/WDS induced protein domain containing protein | -1.149825478 | 0.020826775 |
| Soltu.DM.03G018570 | kunitz trypsin inhibitor | -1.122612101 | 0.020913954 |
| Soltu.DM.09G004080 | Family of unknown function (DUF662) | -1.122412722 | 0.021236982 |
| Soltu.DM.03G025600 | cytochrome P450, family 71, subfamily A, polypeptide | -1.116908769 | 0.021249909 |
| Soltu.DM.04G020610 | cytochrome P450, family 71, subfamily A, polypeptide | -1.116521422 | 0.021589565 |
| Soltu.DM.03G025110 | peptidemethionine sulfoxide reductase | -1.106959554 | 0.022106153 |
| Soltu.DM.11G018460 | NIN like protein | -1.101426871 | 0.022143138 |
| Soltu.DM.07G001250 | cytochrome P450, family 72, subfamily A, polypeptide | -1.099948561 | 0.022258266 |
| Soltu.DM.04G000060 | Matrixin family protein | -1.095213369 | 0.022482266 |
| Soltu.DM.08G018140 | Eukaryotic aspartyl protease family protein | -1.086781801 | 0.022668598 |
| Soltu.DM.02G004730 | auxin response factor | -1.083669072 | 0.023135182 |
| Soltu.DM.01G047150 | Granulin repeat cysteine protease family protein | -1.073866773 | 0.023488316 |
| Soltu.DM.05G022380 | alpha-soluble NSF attachment protein | -1.069723522 | 0.023597239 |
| Soltu.DM.06G009540 | alfin-like | -1.06125167 | 0.023644201 |
| Soltu.DM.01G047120 | Granulin repeat cysteine protease family protein | -1.058760917 | 0.023881603 |
| Soltu.DM.07G012760 | Subtilase family protein | -1.051285014 | 0.024161245 |
| Soltu.DM.12G006690 | sequence-specific DNA binding transcription factors;transcription regulators | -1.050496628 | 0.024273193 |
| Soltu.DM.05G025360 | ALA-interacting subunit | -1.042268094 | 0.024459598 |
| Soltu.DM.10G025810 | cytochrome P450, family 82, subfamily C, polypeptide | -1.030257929 | 0.024467182 |
| Soltu.DM.04G035840 | basic leucine-zipper | -1.022865386 | 0.024537604 |
| Soltu.DM.06G011680 | WRKY DNA-binding protein | -1.018348995 | 0.024794758 |
| Soltu.DM.03G035590 | GATA transcription factor | -1.017628988 | 0.024893449 |
| Soltu.DM.01G046890 | Plant basic secretory protein (BSP) family protein | -1.009419611 | 0.024977078 |
| Soltu.DM.01G047740 | cytochrome P450, family 707, subfamily A, polypeptide | 1.003128391 | 0.024983431 |
| Soltu.DM.04G028130 | WRKY DNA-binding protein | 1.003839729 | 0.025041099 |
| Soltu.DM.04G033040 | cytochrome P450, family 81, subfamily D, polypeptide | 1.012858686 | 0.025242591 |
| Soltu.DM.04G037030 | calcium-dependent protein kinase | 1.014945285 | 0.025314646 |
| Soltu.DM.01G048330 | methyl esterase | 1.037160075 | 0.025324072 |
| Soltu.DM.01G048010 | serine carboxypeptidase-like | 1.043338688 | 0.025889654 |
| Soltu.DM.01G042270 | DNA-directed RNA polymerase III subunit Rpc31 domain containing protein | 1.049550464 | 0.026042439 |
| Soltu.DM.11G026790 | Pentatricopeptide repeat (PPR) superfamily protein | 1.052306704 | 0.026413716 |
| Soltu.DM.05G007790 | Major facilitator superfamily protein | 1.057535115 | 0.026439088 |
| Soltu.DM.01G010340 | plastid-encoded CLP P | 1.05955495 | 0.026899957 |
| Soltu.DM.08G012280 | Duplicated homeodomain-like superfamily protein | 1.068958515 | 0.026926766 |
| Soltu.DM.10G005400 | Eukaryotic aspartyl protease family protein | 1.069326429 | 0.027054805 |
| Soltu.DM.06G028370 | SPFH/Band 7/PHB domain-containing membrane-associated protein family | 1.071321261 | 0.02724686 |
| Soltu.DM.09G013560 | PHD finger transcription factor, putative | 1.082173036 | 0.0273208 |
| Soltu.DM.08G015950 | plant natriuretic peptide A | 1.098844502 | 0.027324267 |
| Soltu.DM.06G005910 | nuclear factor Y, subunit C10 | 1.113962413 | 0.027389901 |
| Soltu.DM.08G024290 | Tetratricopeptide repeat (TPR)-like superfamily protein | 1.122364378 | 0.027582367 |
| Soltu.DM.01G035170 | Eukaryotic aspartyl protease family protein | 1.122426114 | 0.02776611 |
| Soltu.DM.05G021010 | high mobility group A | 1.123043183 | 0.027799122 |
| Soltu.DM.12G029870 | Subtilase family protein | 1.12500915 | 0.027810562 |
| Soltu.DM.12G000540 | basic helix-loop-helix (bHLH) DNA-binding superfamily protein | 1.125321613 | 0.027900827 |
| Soltu.DM.04G000050 | Matrixin family protein | 1.13301457 | 0.027940355 |
| Soltu.DM.02G031430 | hypothetical protein | 1.137989 | 0.02796024 |
| Soltu.DM.12G011520 | Integrase-type DNA-binding superfamily protein | 1.139608261 | 0.028148772 |
| Soltu.DM.03G000640 | myb domain protein | 1.14897092 | 0.028461159 |
| Soltu.DM.07G014900 | Eukaryotic aspartyl protease family protein | 1.165953353 | 0.028650006 |
| Soltu.DM.07G021390 | cytochrome P450, family 72, subfamily A, polypeptide | 1.171408912 | 0.028878007 |
| Soltu.DM.05G011750 | nuclear factor Y, subunit C13 | 1.17873187 | 0.029259973 |
| Soltu.DM.01G045500 | AGAMOUS-like | 1.178800507 | 0.029688202 |
| Soltu.DM.09G021200 | ethylene response factor | 1.199628196 | 0.029734712 |
| Soltu.DM.07G021370 | cytochrome P450, family 72, subfamily A, polypeptide | 1.208336058 | 0.029844699 |
| Soltu.DM.09G030850 | cytochrome P450, family 76, subfamily C, polypeptide | 1.221691546 | 0.029992519 |
| Soltu.DM.10G005720 | arabinogalactan protein | 1.226121836 | 0.030265648 |
| Soltu.DM.07G023130 | Peptidase S24/S26A/S26B/S26C family protein | 1.22714658 | 0.03064701 |
| Soltu.DM.06G030840 | nitrate excretion transporter1 | 1.229287837 | 0.030928624 |
| Soltu.DM.08G017970 | Acyl-CoA N-acyltransferases (NAT) superfamily protein | 1.242480259 | 0.03113983 |
| Soltu.DM.05G009530 | Protein of Unknown Function (DUF239) | 1.251832071 | 0.031277675 |
| Soltu.DM.08G024150 | ethylene responsive element binding factor | 1.267691699 | 0.031791885 |
| Soltu.DM.04G022660 | cytochrome P450, family 71, subfamily A, polypeptide | 1.287787536 | 0.032443311 |
| Soltu.DM.09G009490 | WRKY DNA-binding protein | 1.301053969 | 0.032751169 |
| Soltu.DM.03G018690 | Kunitz family trypsin and protease inhibitor protein | 1.309283523 | 0.032866395 |
| Soltu.DM.02G034320 | WRKY DNA-binding protein | 1.31116649 | 0.032867105 |
| Soltu.DM.02G030620 | reversibly glycosylated polypeptide | 1.313532921 | 0.033089108 |
| Soltu.DM.06G026620 | Dof-type zinc finger DNA-binding family protein | 1.326705182 | 0.033176887 |
| Soltu.DM.12G029330 | NAC (No Apical Meristem) domain transcriptional regulator superfamily protein | 1.327843969 | 0.03335251 |
| Soltu.DM.02G020760 | Regulator of chromosome condensation (RCC1) family with FYVE zinc finger domain | 1.346238867 | 0.033946448 |
| Soltu.DM.03G002030 | LEUNIG_homolog | 1.346424953 | 0.0344959 |
| Soltu.DM.06G001970 | breast cancer associated RING | 1.347916635 | 0.034589052 |
| Soltu.DM.03G018440 | Kunitz family trypsin and protease inhibitor protein | 1.354819741 | 0.034603608 |
| Soltu.DM.04G033280 | Serine/threonine-protein kinase WNK (With No Lysine)-related | 1.357307771 | 0.034615427 |
| Soltu.DM.06G018830 | Tetratricopeptide repeat (TPR)-like superfamily protein | 1.365868828 | 0.035059857 |
| Soltu.DM.09G020830 | WRKY DNA-binding protein | 1.379351188 | 0.035149041 |
| Soltu.DM.04G021540 | tetratricopeptide repeat (TPR)-containing protein | 1.388717844 | 0.035191518 |
| Soltu.DM.01G051020 | proteasome alpha subunit D2 | 1.389358484 | 0.035267211 |
| Soltu.DM.01G050150 | GATA transcription factor | 1.401051894 | 0.035359803 |
| Soltu.DM.04G008550 | hydroxyproline-rich glycoprotein family protein | 1.40297827 | 0.035368197 |
| Soltu.DM.02G004750 | auxin response factor | 1.432876413 | 0.035370258 |
| Soltu.DM.10G024480 | myb domain protein | 1.448201616 | 0.035463186 |
| Soltu.DM.03G025590 | cytochrome P450, family 71, subfamily A, polypeptide | 1.455617686 | 0.035610645 |
| Soltu.DM.08G007360 | TEOSINTE BRANCHED 1, cycloidea and PCF transcription factor | 1.461072721 | 0.035636573 |
| Soltu.DM.08G029870 | Serine/threonine-protein kinase WNK (With No Lysine)-related | 1.463859232 | 0.035765348 |
| Soltu.DM.03G022260 | Tetratricopeptide repeat (TPR)-like superfamily protein | 1.46670618 | 0.035966799 |
| Soltu.DM.02G007690 | alpha/beta-Hydrolases superfamily protein | 1.468004733 | 0.036802887 |
| Soltu.DM.09G001760 | basic helix-loop-helix (bHLH) DNA-binding superfamily protein | 1.486646118 | 0.036816265 |
| Soltu.DM.07G019030 | MYB-like | 1.493951753 | 0.036848856 |
| Soltu.DM.05G006080 | Regulator of chromosome condensation (RCC1) family with FYVE zinc finger domain | 1.494308428 | 0.036870263 |
| Soltu.DM.08G007130 | jasmonate-zim-domain protein | 1.498524431 | 0.036881303 |
| Soltu.DM.03G025510 | cytochrome P450, family 71, subfamily A, polypeptide | 1.5177408 | 0.037019399 |
| Soltu.DM.04G038210 | PLATZ transcription factor family protein | 1.523450882 | 0.037163796 |
| Soltu.DM.11G021030 | Eukaryotic aspartyl protease family protein | 1.528027497 | 0.037270443 |
| Soltu.DM.01G036060 | Major facilitator superfamily protein | 1.542204122 | 0.037406511 |
| Soltu.DM.04G031720 | Leucine-rich repeat transmembrane protein kinase family protein | 1.544332111 | 0.037739926 |
| Soltu.DM.01G038350 | Signal peptidase subunit | 1.547960328 | 0.037779277 |
| Soltu.DM.10G030030 | cytochrome P450, family 98, subfamily A, polypeptide | 1.562205793 | 0.037861661 |
| Soltu.DM.03G023710 | Kunitz family trypsin and protease inhibitor protein | 1.569556946 | 0.037940063 |
| Soltu.DM.08G026640 | Subtilase family protein | 1.575654907 | 0.03810896 |
| Soltu.DM.12G005320 | proteasome alpha subunit F1 | 1.592456599 | 0.038263867 |
| Soltu.DM.04G021440 | Major facilitator superfamily protein | 1.601446658 | 0.038441361 |
| Soltu.DM.08G014330 | haloacid dehalogenase-like hydrolase family protein | 1.643452638 | 0.038456286 |
| Soltu.DM.10G003670 | cytochrome P450, family 72, subfamily A, polypeptide | 1.662263208 | 0.038635522 |
| Soltu.DM.03G014050 | myb domain protein | 1.692286619 | 0.038740085 |
| Soltu.DM.08G000200 | Subtilase family protein | 1.72516228 | 0.038944882 |
| Soltu.DM.08G028970 | Integrase-type DNA-binding superfamily protein | 1.773649192 | 0.039022206 |
| Soltu.DM.11G017460 | Peptidase M28 family protein | 1.782201449 | 0.039515313 |
| Soltu.DM.01G024140 | Eukaryotic aspartyl protease family protein | 1.784496719 | 0.039669293 |
| Soltu.DM.11G023000 | Homeobox-leucine zipper family protein / lipid-binding START domain-containing protein | 1.822585281 | 0.03974488 |
| Soltu.DM.03G004330 | homeobox protein | 1.833554148 | 0.039767328 |
| Soltu.DM.06G031580 | TRF-like | 1.867885473 | 0.040075947 |
| Soltu.DM.01G049470 | alpha/beta-Hydrolases superfamily protein | 1.905160409 | 0.040254747 |
| Soltu.DM.07G012300 | DZC (Disease resistance/zinc finger/chromosome condensation-like region) domain containing protein | 1.93099936 | 0.040342315 |
| Soltu.DM.12G029240 | Cyclophilin-like peptidyl-prolyl cis-trans isomerase family protein | 1.951492675 | 0.040419454 |
| Soltu.DM.03G025100 | BTB and TAZ domain protein | 1.961460487 | 0.040443833 |
| Soltu.DM.06G026950 | BTB and TAZ domain protein | 1.983614396 | 0.040456965 |
| Soltu.DM.11G026620 | myb domain protein | 1.993558962 | 0.041049543 |
| Soltu.DM.08G029670 | NIN like protein | 2.019055128 | 0.041137244 |
| Soltu.DM.12G019530 | serine carboxypeptidase-like | 2.030402457 | 0.041335894 |
| Soltu.DM.11G025890 | related to AP2.7 | 2.082207029 | 0.042295237 |
| Soltu.DM.07G023410 | BES1/BZR1 homolog | 2.114429574 | 0.042359572 |
| Soltu.DM.03G005730 | Granulin repeat cysteine protease family protein | 2.115175443 | 0.042538253 |
| Soltu.DM.01G024120 | Eukaryotic aspartyl protease family protein | 2.135807688 | 0.042670062 |
| Soltu.DM.01G044540 | metacaspase | 2.145768247 | 0.04285947 |
| Soltu.DM.08G029850 | Dof-type zinc finger DNA-binding family protein | 2.177795037 | 0.04299267 |
| Soltu.DM.04G005970 | NAC (No Apical Meristem) domain transcriptional regulator superfamily protein | 2.180483992 | 0.043044396 |
| Soltu.DM.04G032200 | Integrase-type DNA-binding superfamily protein | 2.184618077 | 0.044080294 |
| Soltu.DM.04G010460 | proton gradient regulation | 2.204114713 | 0.044460155 |
| Soltu.DM.03G029180 | Eukaryotic aspartyl protease family protein | 2.287626469 | 0.04503712 |
| Soltu.DM.08G021480 | DZC (Disease resistance/zinc finger/chromosome condensation-like region) domain containing protein | 2.315616323 | 0.045201439 |
| Soltu.DM.10G030270 | 20S proteasome alpha subunit G1 | 2.376144594 | 0.045236715 |
| Soltu.DM.04G008700 | basic helix-loop-helix (bHLH) DNA-binding superfamily protein | 2.376968868 | 0.045370044 |
| Soltu.DM.01G023870 | REF4-related | 2.391914798 | 0.045486469 |
| Soltu.DM.07G006830 | basic helix-loop-helix (bHLH) DNA-binding superfamily protein | 2.477254863 | 0.045647012 |
| Soltu.DM.02G013910 | conserved peptide upstream open reading frame | 2.478400715 | 0.045986517 |
| Soltu.DM.07G021690 | heat shock transcription factor A4A | 2.505543375 | 0.046125343 |
| Soltu.DM.10G001290 | Cell differentiation, Rcd1-like protein | 2.615222642 | 0.04644583 |
| Soltu.DM.04G033210 | N-terminal nucleophile aminohydrolases (Ntn hydrolases) superfamily protein | 2.63634494 | 0.046907128 |
| Soltu.DM.01G005400 | cytochrome P450, family 71, subfamily B, polypeptide | 2.652152977 | 0.046943138 |
| Soltu.DM.10G019280 | DRE-binding protein 2A | 2.658469276 | 0.047346397 |
| Soltu.DM.11G002540 | myb domain protein | 2.662897645 | 0.047565691 |
| Soltu.DM.08G008460 | Tetratricopeptide repeat (TPR)-like superfamily protein | 2.678661698 | 0.047593943 |
| Soltu.DM.02G016830 | Optic atrophy 3 protein (OPA3) | 2.718237745 | 0.047673559 |
| Soltu.DM.04G021630 | Integrase-type DNA-binding superfamily protein | 2.7353111 | 0.048065815 |
| Soltu.DM.09G001130 | Protein kinase protein with tetratricopeptide repeat domain | 2.860039061 | 0.048196975 |
| Soltu.DM.08G015780 | dicer-like | 3.06467609 | 0.048467099 |
| Soltu.DM.03G004970 | DegP protease | 4.080820865 | 0.04873409 |
| Soltu.DM.09G015230 | alpha/beta-Hydrolases superfamily protein | 4.311513015 | 0.049137862 |
| Soltu.DM.06G014270 | Signal recognition particle, SRP9/SRP14 subunit | 4.311623701 | 0.049699543 |
| Soltu.DM.01G005630 | signal peptide peptidase | 4.37560211 | 0.049937586 |
| Soltu.DM.07G020890 | sequence-specific DNA binding transcription factors | 4.86777301 | 0.049974105 |
| Effector-triggered immunity，ETI | | | |
| Soltu.DM.05G009940 | hypothetical protein | -10.1233128 | 1.36E-06 |
| Soltu.DM.09G007850 | MLP-like protein | -6.897917602 | 8.26E-06 |
| Soltu.DM.04G029230 | conserved hypothetical protein | -6.728294134 | 4.80E-05 |
| Soltu.DM.09G007770 | MLP-like protein | -6.214147711 | 4.81E-05 |
| Soltu.DM.10G003070 | conserved hypothetical protein | -6.192038556 | 0.00016254 |
| Soltu.DM.03G025840 | hypothetical protein | -6.009253052 | 0.000251317 |
| Soltu.DM.11G023450 | hypothetical protein | -6.005429108 | 0.00025442 |
| Soltu.DM.10G027580 | hypothetical protein | -5.853680286 | 0.000266593 |
| Soltu.DM.05G019270 | cytochrome p450 78a9 | -5.366981821 | 0.000474576 |
| Soltu.DM.04G002830 | hypothetical protein | -5.346458175 | 0.00049562 |
| Soltu.DM.08G019300 | Protein of unknown function (DUF_B2219) domain containing protein | -5.29067567 | 0.000498744 |
| Soltu.DM.07G006680 | conserved hypothetical protein | -5.160855716 | 0.000537847 |
| Soltu.DM.12G029060 | hypothetical protein | -5.044566914 | 0.000684714 |
| Soltu.DM.03G034460 | conserved hypothetical protein | -4.813559092 | 0.000691828 |
| Soltu.DM.04G008330 | conserved hypothetical protein | -4.521181131 | 0.000822908 |
| Soltu.DM.05G012430 | senescence-associated gene | -4.50400061 | 0.000985151 |
| Soltu.DM.10G001270 | hypothetical protein | -4.367572255 | 0.001048266 |
| Soltu.DM.12G007260 | hypothetical protein | -4.347403382 | 0.001060546 |
| Soltu.DM.11G010180 | sucrose-proton symporter | -4.321981374 | 0.001338823 |
| Soltu.DM.02G024880 | conserved hypothetical protein | -3.551209572 | 0.001382719 |
| Soltu.DM.01G050940 | Major facilitator superfamily protein | -3.43918743 | 0.00148178 |
| Soltu.DM.06G020490 | late embryogenesis abundant domain-containing protein / LEA domain-containing protein | -3.408461152 | 0.001838795 |
| Soltu.DM.02G024910 | Chaperone DnaJ-domain superfamily protein | -3.396685208 | 0.001878965 |
| Soltu.DM.01G044930 | hypothetical protein | -3.275186695 | 0.002103311 |
| Soltu.DM.11G007740 | hypothetical protein | -3.273154005 | 0.002453212 |
| Soltu.DM.02G015380 | conserved hypothetical protein | -3.194503981 | 0.002505953 |
| Soltu.DM.07G002120 | UDP-glucosyl transferase 73B5 | -3.14040339 | 0.002532524 |
| Soltu.DM.04G037690 | Patched family protein | -3.075815795 | 0.002640593 |
| Soltu.DM.04G025520 | conserved hypothetical protein | -2.999244571 | 0.002820809 |
| Soltu.DM.02G026840 | conserved hypothetical protein | -2.977131764 | 0.003001813 |
| Soltu.DM.09G026550 | conserved hypothetical protein | -2.903201831 | 0.003045176 |
| Soltu.DM.11G023870 | Nucleotide-diphospho-sugar transferases superfamily protein | -2.876519346 | 0.00323793 |
| Soltu.DM.10G011430 | Seven transmembrane MLO family protein | -2.873390302 | 0.003460349 |
| Soltu.DM.09G031070 | UDP-Glycosyltransferase superfamily protein | -2.867097703 | 0.003481442 |
| Soltu.DM.05G004570 | conserved hypothetical protein | -2.758658163 | 0.003847146 |
| Soltu.DM.07G022270 | Cytochrome P450 superfamily protein | -2.731251911 | 0.003919321 |
| Soltu.DM.10G005370 | conserved hypothetical protein | -2.73048885 | 0.004156854 |
| Soltu.DM.09G024960 | conserved hypothetical protein | -2.707194851 | 0.004159517 |
| Soltu.DM.05G024390 | NB-ARC domain-containing disease resistance protein | -2.684529249 | 0.004191973 |
| Soltu.DM.05G002750 | hypothetical protein | -2.672717062 | 0.004519097 |
| Soltu.DM.06G010720 | HOPZ-ACTIVATED RESISTANCE | -2.670414133 | 0.004760215 |
| Soltu.DM.10G013190 | Concanavalin A-like lectin protein kinase family protein | -2.656566808 | 0.004769325 |
| Soltu.DM.02G026570 | conserved hypothetical protein | -2.64841155 | 0.004800518 |
| Soltu.DM.01G000540 | hypothetical protein | -2.623199483 | 0.004886704 |
| Soltu.DM.06G023290 | 2-oxoglutarate (2OG) and Fe(II)-dependent oxygenase superfamily protein | -2.588919831 | 0.005050022 |
| Soltu.DM.03G029230 | exocyst subunit exo70 family protein E2 | -2.582594587 | 0.005110423 |
| Soltu.DM.03G032380 | conserved hypothetical protein | -2.575036547 | 0.005184762 |
| Soltu.DM.09G031020 | UDP-Glycosyltransferase superfamily protein | -2.566187899 | 0.005274132 |
| Soltu.DM.09G005740 | Calcium-dependent lipid-binding (CaLB domain) family protein | -2.518994 | 0.005311005 |
| Soltu.DM.05G025480 | conserved hypothetical protein | -2.478980024 | 0.005330905 |
| Soltu.DM.08G028450 | hypothetical protein | -2.47315983 | 0.005365705 |
| Soltu.DM.09G017300 | conserved hypothetical protein | -2.460263672 | 0.005371216 |
| Soltu.DM.08G027650 | root hair specific | -2.445695642 | 0.005513779 |
| Soltu.DM.02G030290 | hypothetical protein | -2.411436945 | 0.005583665 |
| Soltu.DM.01G042410 | conserved hypothetical protein | -2.410293852 | 0.00567544 |
| Soltu.DM.01G014450 | Got1/Sft2-like vescicle transport protein family | -2.380198774 | 0.006133546 |
| Soltu.DM.11G006330 | Integral membrane Yip1 family protein | -2.366657031 | 0.006154664 |
| Soltu.DM.04G032240 | hypothetical protein | -2.355139963 | 0.006233589 |
| Soltu.DM.06G014360 | conserved hypothetical protein | -2.296571984 | 0.006289931 |
| Soltu.DM.05G009070 | hypothetical protein | -2.28119831 | 0.006451167 |
| Soltu.DM.04G033200 | hypothetical protein | -2.262326586 | 0.006636232 |
| Soltu.DM.09G018580 | Integral membrane HRF1 family protein | -2.2606268 | 0.006647097 |
| Soltu.DM.03G011440 | PHE ammonia lyase | -2.255931877 | 0.006868598 |
| Soltu.DM.10G022920 | Arabidopsis Inositol phosphorylceramide synthase | -2.252656857 | 0.006872797 |
| Soltu.DM.03G005950 | conserved hypothetical protein | -2.220121389 | 0.006892172 |
| Soltu.DM.03G020520 | conserved hypothetical protein | -2.208711919 | 0.006969453 |
| Soltu.DM.04G037830 | glutamate receptor 3.3 | -2.185205304 | 0.007258294 |
| Soltu.DM.04G030420 | conserved hypothetical protein | -2.175723818 | 0.007397069 |
| Soltu.DM.09G022150 | conserved hypothetical protein | -2.154722645 | 0.007473506 |
| Soltu.DM.06G001170 | hypothetical protein | -2.119081526 | 0.007645146 |
| Soltu.DM.11G008510 | receptor-like protein kinase | -2.073858076 | 0.007705097 |
| Soltu.DM.03G022200 | heat shock protein 70 (Hsp 70) family protein | -2.068702196 | 0.007707618 |
| Soltu.DM.01G026210 | hypothetical protein | -2.066611571 | 0.007748248 |
| Soltu.DM.01G004960 | hypothetical protein | -2.060514031 | 0.007885743 |
| Soltu.DM.12G006820 | hypothetical protein | -2.046328681 | 0.008104634 |
| Soltu.DM.10G016100 | B-cell receptor-associated 31-like | -2.036090649 | 0.008143255 |
| Soltu.DM.01G000480 | NB-ARC domain-containing disease resistance protein | -2.030964168 | 0.00820995 |
| Soltu.DM.08G019670 | hypothetical protein | -2.014887119 | 0.008305591 |
| Soltu.DM.01G036110 | aminophospholipid ATPase | -2.010654871 | 0.008434524 |
| Soltu.DM.05G005160 | hypothetical protein | -2.007110004 | 0.008955396 |
| Soltu.DM.04G020510 | conserved hypothetical protein | -2.006591556 | 0.009078558 |
| Soltu.DM.05G017050 | acireductone dioxygenase | -1.976575192 | 0.009414453 |
| Soltu.DM.09G021040 | hypothetical protein | -1.967249454 | 0.009545456 |
| Soltu.DM.08G029740 | hypothetical protein | -1.942358994 | 0.009573437 |
| Soltu.DM.04G028470 | RING/U-box superfamily protein | -1.931603816 | 0.009758115 |
| Soltu.DM.11G003400 | conserved hypothetical protein | -1.931202339 | 0.009906719 |
| Soltu.DM.12G006830 | hypothetical protein | -1.928043891 | 0.010006842 |
| Soltu.DM.01G036640 | conserved hypothetical protein | -1.918713649 | 0.010010935 |
| Soltu.DM.10G027460 | UDP-glycosyltransferase 73B4 | -1.912356671 | 0.010095423 |
| Soltu.DM.08G026470 | hypothetical protein | -1.892465774 | 0.010172911 |
| Soltu.DM.07G015350 | conserved hypothetical protein | -1.891382591 | 0.010221696 |
| Soltu.DM.06G033580 | ER lumen protein retaining receptor family protein | -1.875695358 | 0.01039361 |
| Soltu.DM.02G020160 | hypothetical protein | -1.874309037 | 0.010815158 |
| Soltu.DM.01G025960 | Auxin efflux carrier family protein | -1.865424755 | 0.011166353 |
| Soltu.DM.03G020240 | hypothetical protein | -1.865255227 | 0.011296534 |
| Soltu.DM.08G028460 | Late embryogenesis abundant (LEA) hydroxyproline-rich glycoprotein family | -1.85677716 | 0.011350433 |
| Soltu.DM.01G040290 | HIT zinc finger ;PAPA-1-like conserved region | -1.85529417 | 0.011394681 |
| Soltu.DM.06G019550 | conserved hypothetical protein | -1.854091645 | 0.011411397 |
| Soltu.DM.07G002960 | prephenate dehydrogenase family protein | -1.852600196 | 0.01153431 |
| Soltu.DM.09G018170 | vesicle-associated membrane protein | -1.841724105 | 0.011930864 |
| Soltu.DM.10G021500 | Modifier of rudimentary (Mod(r)) protein | -1.834660732 | 0.012069028 |
| Soltu.DM.12G006800 | hypothetical protein | -1.828263018 | 0.012131186 |
| Soltu.DM.04G006830 | threonine aldolase | -1.827488609 | 0.012213901 |
| Soltu.DM.02G003520 | conserved hypothetical protein | -1.809583809 | 0.012476207 |
| Soltu.DM.03G035440 | conserved hypothetical protein | -1.789071347 | 0.012542842 |
| Soltu.DM.03G011450 | PHE ammonia lyase | -1.780498936 | 0.012575002 |
| Soltu.DM.10G009910 | conserved hypothetical protein | -1.768838549 | 0.012741357 |
| Soltu.DM.06G000920 | hypothetical protein | -1.768483409 | 0.012810451 |
| Soltu.DM.01G014440 | SRP72 RNA-binding domain | -1.763640542 | 0.012880032 |
| Soltu.DM.04G028750 | hypothetical protein | -1.761885533 | 0.013034495 |
| Soltu.DM.01G022220 | conserved hypothetical protein | -1.759616449 | 0.013196039 |
| Soltu.DM.04G000010 | hypothetical protein | -1.751189973 | 0.013246697 |
| Soltu.DM.12G029850 | conserved hypothetical protein | -1.748539971 | 0.013275882 |
| Soltu.DM.01G002670 | syntaxin of plants | -1.731136336 | 0.013360022 |
| Soltu.DM.01G042510 | Protein of unknown function (DUF679) | -1.703209369 | 0.013405251 |
| Soltu.DM.08G029210 | hypothetical protein | -1.700187794 | 0.013498524 |
| Soltu.DM.02G028070 | Auxin-responsive GH3 family protein | -1.692211808 | 0.013515693 |
| Soltu.DM.04G023810 | conserved hypothetical protein | -1.687582275 | 0.013648425 |
| Soltu.DM.05G023760 | phloem protein 2-B12 | -1.685656824 | 0.013740633 |
| Soltu.DM.01G024680 | DNAJ heat shock family protein | -1.68408374 | 0.013758161 |
| Soltu.DM.02G026510 | hypothetical protein | -1.672167645 | 0.013867167 |
| Soltu.DM.04G037080 | conserved hypothetical protein | -1.670217604 | 0.013969367 |
| Soltu.DM.07G022560 | glutathione S-transferase TAU | -1.667961169 | 0.013969774 |
| Soltu.DM.04G034690 | allene oxide synthase | -1.66207277 | 0.014026059 |
| Soltu.DM.01G045840 | conserved hypothetical protein | -1.655397402 | 0.014065703 |
| Soltu.DM.01G029090 | glutathione S-transferase tau | -1.650172195 | 0.014073342 |
| Soltu.DM.06G026640 | HCO3- transporter family | -1.642940588 | 0.014125483 |
| Soltu.DM.12G019570 | formin homology | -1.625695488 | 0.014364147 |
| Soltu.DM.07G002540 | conserved hypothetical protein | -1.615751332 | 0.014429586 |
| Soltu.DM.01G019950 | Ras-related small GTP-binding family protein | -1.612107083 | 0.014652243 |
| Soltu.DM.11G003570 | 3-deoxy-D-arabino-heptulosonate 7-phosphate synthase | -1.6107509 | 0.014887925 |
| Soltu.DM.01G030410 | PRA1 (Prenylated rab acceptor) family protein | -1.592236999 | 0.014892693 |
| Soltu.DM.08G024000 | FAD/NAD(P)-binding oxidoreductase family protein | -1.590450081 | 0.01497307 |
| Soltu.DM.04G002100 | conserved hypothetical protein | -1.588630678 | 0.015030638 |
| Soltu.DM.03G000460 | Sec23/Sec24 protein transport family protein | -1.5814261 | 0.015196134 |
| Soltu.DM.11G008890 | conserved hypothetical protein | -1.579651619 | 0.015229358 |
| Soltu.DM.06G025650 | soluble N-ethylmaleimide-sensitive factor adaptor protein | -1.577475328 | 0.015288328 |
| Soltu.DM.10G005810 | Fatty acid/sphingolipid desaturase | -1.575205944 | 0.015300774 |
| Soltu.DM.03G021640 | conserved hypothetical protein | -1.571769247 | 0.015302315 |
| Soltu.DM.04G033890 | UDP-glucose:flavonoid 3-o-glucosyltransferase | -1.568729658 | 0.015308721 |
| Soltu.DM.10G019260 | prenylated RAB acceptor 1.B4 | -1.563985587 | 0.015383687 |
| Soltu.DM.08G019140 | Protein of unknown function (DUF_B2219) domain containing protein | -1.561119222 | 0.015602046 |
| Soltu.DM.08G006350 | hypothetical protein | -1.550770223 | 0.015740231 |
| Soltu.DM.05G023770 | F-box family protein | -1.549107007 | 0.015774821 |
| Soltu.DM.01G001940 | hypothetical protein | -1.543850208 | 0.015806257 |
| Soltu.DM.01G028580 | conserved hypothetical protein | -1.534993544 | 0.015836597 |
| Soltu.DM.11G021410 | hypothetical protein | -1.530618629 | 0.015911 |
| Soltu.DM.07G027120 | conserved hypothetical protein | -1.530351063 | 0.016057604 |
| Soltu.DM.09G031520 | hypothetical protein | -1.52116958 | 0.016059019 |
| Soltu.DM.02G034230 | Ferritin/ribonucleotide reductase-like family protein | -1.520665287 | 0.016160191 |
| Soltu.DM.09G009300 | prenylated RAB acceptor 1.B4 | -1.514368012 | 0.016342385 |
| Soltu.DM.04G006420 | hypothetical protein | -1.514083125 | 0.016355743 |
| Soltu.DM.11G005680 | UDP-Glycosyltransferase superfamily protein | -1.509697303 | 0.016367153 |
| Soltu.DM.07G021660 | S-domain-2 | -1.504110639 | 0.016443688 |
| Soltu.DM.02G014910 | Leucine-rich repeat transmembrane protein kinase | -1.501471635 | 0.016509187 |
| Soltu.DM.05G001680 | hypothetical protein | -1.500964624 | 0.016512968 |
| Soltu.DM.01G027100 | conserved hypothetical protein | -1.494506518 | 0.016575623 |
| Soltu.DM.02G026260 | hypothetical protein | -1.483843315 | 0.016800087 |
| Soltu.DM.10G004010 | targeting protein for XKLP2 | -1.474771831 | 0.016863741 |
| Soltu.DM.03G000760 | translocase of outer membrane 22-V | -1.468692113 | 0.016933248 |
| Soltu.DM.12G006810 | hypothetical protein | -1.460646146 | 0.016995384 |
| Soltu.DM.12G029830 | UDP-Glycosyltransferase superfamily protein | -1.459200496 | 0.017006691 |
| Soltu.DM.06G013450 | beta-carotene hydroxylase | -1.456616903 | 0.017215953 |
| Soltu.DM.06G029980 | hypothetical protein | -1.435729066 | 0.017312411 |
| Soltu.DM.04G007470 | conserved hypothetical protein | -1.429081568 | 0.017369641 |
| Soltu.DM.07G023820 | hypothetical protein | -1.420750302 | 0.017618602 |
| Soltu.DM.06G001150 | hypothetical protein | -1.412599745 | 0.017848407 |
| Soltu.DM.06G033590 | ER lumen protein retaining receptor family protein | -1.411250289 | 0.018007354 |
| Soltu.DM.03G018340 | hypothetical protein | -1.406288755 | 0.018018026 |
| Soltu.DM.01G021920 | vesicle-associated membrane protein | -1.40128327 | 0.018040989 |
| Soltu.DM.01G046490 | conserved hypothetical protein | -1.398301865 | 0.018107411 |
| Soltu.DM.12G009550 | Cytochrome P450 superfamily protein | -1.396097604 | 0.018159536 |
| Soltu.DM.03G011430 | UDP-Glycosyltransferase superfamily protein | -1.391828089 | 0.018185986 |
| Soltu.DM.04G027670 | Protein of unknown function (DUF3511) | -1.367397852 | 0.018320538 |
| Soltu.DM.07G019760 | conserved hypothetical protein | -1.361938334 | 0.0184212 |
| Soltu.DM.10G013030 | conserved hypothetical protein | -1.361117238 | 0.018443568 |
| Soltu.DM.03G022700 | hypothetical protein | -1.352309079 | 0.01855298 |
| Soltu.DM.04G005650 | conserved hypothetical protein | -1.351363717 | 0.018735974 |
| Soltu.DM.02G007770 | Auxin-responsive GH3 family protein | -1.349189431 | 0.019110848 |
| Soltu.DM.02G034300 | conserved hypothetical protein | -1.322430168 | 0.019620143 |
| Soltu.DM.03G024900 | cysteine-rich RLK (RECEPTOR-like protein kinase) | -1.31409151 | 0.019828124 |
| Soltu.DM.11G025970 | hypothetical protein | -1.313436054 | 0.019854359 |
| Soltu.DM.12G006840 | hypothetical protein | -1.313100729 | 0.019959443 |
| Soltu.DM.11G008380 | receptor-like protein kinase | -1.302099068 | 0.019978105 |
| Soltu.DM.05G000240 | conserved hypothetical protein | -1.301032658 | 0.020067231 |
| Soltu.DM.10G024730 | cytochrome P450, family 76, subfamily G, polypeptide | -1.28731511 | 0.020181952 |
| Soltu.DM.11G021070 | magnesium (Mg) transporter | -1.287279449 | 0.02047146 |
| Soltu.DM.12G028090 | RING membrane-anchor | -1.283492413 | 0.020530915 |
| Soltu.DM.03G037120 | lipoxygenase | -1.270552565 | 0.020547912 |
| Soltu.DM.11G018490 | hypothetical protein | -1.2702083 | 0.020763191 |
| Soltu.DM.03G034640 | pyridoxine biosynthesis 1.2 | -1.263105026 | 0.020851078 |
| Soltu.DM.11G005450 | UDP-Glycosyltransferase superfamily protein | -1.258732392 | 0.02096757 |
| Soltu.DM.04G034820 | GNS1/SUR4 membrane protein family | -1.257693255 | 0.021078994 |
| Soltu.DM.03G005400 | N-acetyl-l-glutamate synthase | -1.240326934 | 0.021109918 |
| Soltu.DM.03G036780 | conserved hypothetical protein | -1.238381403 | 0.021150131 |
| Soltu.DM.01G003160 | Abscisic acid-responsive (TB2/DP1, HVA22) family protein | -1.236755592 | 0.021228112 |
| Soltu.DM.05G006230 | UDP-galactose transporter | -1.227719799 | 0.021281681 |
| Soltu.DM.12G029250 | conserved hypothetical protein | -1.21879721 | 0.021296159 |
| Soltu.DM.02G015110 | hypothetical protein | -1.210067416 | 0.021404488 |
| Soltu.DM.01G027660 | hypothetical protein | -1.206768734 | 0.021426314 |
| Soltu.DM.12G003240 | hypothetical protein | -1.197676475 | 0.021631707 |
| Soltu.DM.02G017680 | conserved hypothetical protein | -1.19287953 | 0.021666793 |
| Soltu.DM.02G018220 | secretory carrier | -1.187978124 | 0.021948508 |
| Soltu.DM.08G018340 | conserved hypothetical protein | -1.18622211 | 0.021981779 |
| Soltu.DM.03G002690 | Ubiquitin-like superfamily protein | -1.185816734 | 0.022031942 |
| Soltu.DM.01G021050 | UDP-Glycosyltransferase superfamily protein | -1.185533636 | 0.022126559 |
| Soltu.DM.04G022390 | terpene synthase | -1.182006217 | 0.022184892 |
| Soltu.DM.12G010130 | conserved hypothetical protein | -1.173419544 | 0.022425491 |
| Soltu.DM.09G023860 | cellulose synthase-like D5 | -1.166934565 | 0.022582191 |
| Soltu.DM.01G047220 | Protein of unknown function (DUF579) | -1.165749192 | 0.022599081 |
| Soltu.DM.07G004010 | purine permease | -1.164154906 | 0.022612228 |
| Soltu.DM.10G004200 | conserved hypothetical protein | -1.157522289 | 0.02267198 |
| Soltu.DM.08G024020 | RAB GTPase homolog 1C | -1.149148113 | 0.022726017 |
| Soltu.DM.04G001280 | conserved hypothetical protein | -1.147003149 | 0.022748451 |
| Soltu.DM.08G010620 | Ubiquitin-like superfamily protein | -1.146739589 | 0.022835198 |
| Soltu.DM.07G022520 | glutathione S-transferase TAU | -1.146589442 | 0.023094831 |
| Soltu.DM.11G003280 | ENTH/ANTH/VHS superfamily protein | -1.144811218 | 0.023180099 |
| Soltu.DM.10G007100 | isoamylase | -1.144534517 | 0.023263937 |
| Soltu.DM.03G031080 | hypothetical protein | -1.140580672 | 0.023323487 |
| Soltu.DM.05G025980 | Beta-D-glucosyl crocetin beta-1,6-glucosyltransferase | -1.136444295 | 0.023383094 |
| Soltu.DM.01G032570 | UDP-N-acetylglucosamine (UAA) transporter family | -1.126275642 | 0.023433674 |
| Soltu.DM.03G019480 | conserved hypothetical protein | -1.119048151 | 0.023454701 |
| Soltu.DM.03G026850 | hypothetical protein | -1.112155224 | 0.023600736 |
| Soltu.DM.09G017390 | conserved hypothetical protein | -1.106959554 | 0.023635944 |
| Soltu.DM.06G034950 | NPL4-like protein | -1.10354959 | 0.023745575 |
| Soltu.DM.08G000560 | plant VAP homolog | -1.103416933 | 0.024094449 |
| Soltu.DM.02G003530 | hypothetical protein | -1.101426871 | 0.024353565 |
| Soltu.DM.04G002550 | conserved hypothetical protein | -1.101037478 | 0.024424069 |
| Soltu.DM.08G013640 | ferrochelatase | -1.095213369 | 0.024464468 |
| Soltu.DM.02G032380 | galacturonosyltransferase-like | -1.090418704 | 0.025088788 |
| Soltu.DM.11G008440 | receptor-like protein kinase | -1.089930968 | 0.025211499 |
| Soltu.DM.01G043880 | conserved hypothetical protein | -1.086781801 | 0.025316414 |
| Soltu.DM.11G015310 | conserved hypothetical protein | -1.086212971 | 0.025610026 |
| Soltu.DM.08G025570 | cytochrome P450, family 706, subfamily A, polypeptide | -1.085205993 | 0.025676415 |
| Soltu.DM.01G007010 | hypothetical protein | -1.077500141 | 0.025770699 |
| Soltu.DM.10G000690 | conserved hypothetical protein | -1.074263689 | 0.025996899 |
| Soltu.DM.02G020930 | microsomal glutathione s-transferase, putative | -1.073542637 | 0.026035477 |
| Soltu.DM.11G008490 | receptor-like protein kinase | -1.073068949 | 0.026167146 |
| Soltu.DM.04G008340 | conserved hypothetical protein | -1.072921524 | 0.026252213 |
| Soltu.DM.10G004170 | conserved hypothetical protein | -1.072466131 | 0.027076158 |
| Soltu.DM.04G011670 | UDP-glucosyl transferase 85A7 | -1.066371248 | 0.027268129 |
| Soltu.DM.09G002000 | conserved hypothetical protein | -1.052289874 | 0.027300248 |
| Soltu.DM.07G022330 | hypothetical protein | -1.046164498 | 0.027335638 |
| Soltu.DM.08G004500 | 1-aminocyclopropane-1-carboxylic acid (acc) synthase | -1.045740673 | 0.027516318 |
| Soltu.DM.05G007110 | conserved hypothetical protein | -1.040402653 | 0.027553292 |
| Soltu.DM.06G034370 | hypothetical protein | -1.029863616 | 0.027594241 |
| Soltu.DM.04G034100 | phospholipase A 2A | -1.029035865 | 0.027829316 |
| Soltu.DM.04G031280 | HVA22 homologue A | -1.023370471 | 0.027987142 |
| Soltu.DM.10G027480 | UDP-glycosyltransferase 73B4 | -1.022943006 | 0.028117082 |
| Soltu.DM.11G001250 | nonsense-mediated mRNA decay NMD3 family protein | -1.021797418 | 0.028429309 |
| Soltu.DM.01G013420 | hypothetical protein | -1.021559375 | 0.02850457 |
| Soltu.DM.02G026700 | conserved hypothetical protein | -1.018348995 | 0.028574598 |
| Soltu.DM.05G005910 | hypothetical protein | -1.015150234 | 0.028616625 |
| Soltu.DM.03G006190 | hypothetical protein | -1.012579237 | 0.028805405 |
| Soltu.DM.03G011480 | PHE ammonia lyase | -1.006191372 | 0.028894225 |
| Soltu.DM.11G008450 | receptor-like protein kinase | -1.004265962 | 0.028924676 |
| Soltu.DM.05G013050 | RING/U-box superfamily protein | 1.012858686 | 0.029008213 |
| Soltu.DM.07G006300 | Phosphoinositide phosphatase family protein | 1.013568068 | 0.029014729 |
| Soltu.DM.01G026740 | conserved hypothetical protein | 1.02105371 | 0.029045674 |
| Soltu.DM.12G027460 | hypothetical protein | 1.022241451 | 0.029131004 |
| Soltu.DM.05G005670 | hypothetical protein | 1.023056702 | 0.029160968 |
| Soltu.DM.12G027030 | hypothetical protein | 1.040019006 | 0.0292719 |
| Soltu.DM.12G015570 | hypothetical protein | 1.04717883 | 0.029321706 |
| Soltu.DM.02G004170 | B-cell receptor-associated 31-like | 1.063925168 | 0.029334659 |
| Soltu.DM.03G021630 | conserved hypothetical protein | 1.065466261 | 0.0293433 |
| Soltu.DM.06G027220 | secE/sec61-gamma protein transport protein | 1.065694777 | 0.029391833 |
| Soltu.DM.12G018550 | PHYTOENE SYNTHASE | 1.068218564 | 0.029481283 |
| Soltu.DM.03G005030 | NAD(P)H dehydrogenase C1 | 1.08376485 | 0.029499541 |
| Soltu.DM.02G025220 | Cytochrome P450 superfamily protein | 1.091266775 | 0.029544223 |
| Soltu.DM.03G028670 | LAG1 longevity assurance homolog | 1.095076897 | 0.029703459 |
| Soltu.DM.03G014100 | hypothetical protein | 1.105664107 | 0.029722642 |
| Soltu.DM.04G030380 | ENTH/ANTH/VHS superfamily protein | 1.130146463 | 0.02972613 |
| Soltu.DM.01G020290 | hypothetical protein | 1.138835114 | 0.029768361 |
| Soltu.DM.03G004850 | hypothetical protein | 1.139608261 | 0.029898291 |
| Soltu.DM.05G005090 | galactosyltransferase1 | 1.176558299 | 0.030129101 |
| Soltu.DM.02G023370 | early-responsive to dehydration stress protein (ERD4) | 1.182385797 | 0.03024028 |
| Soltu.DM.10G027720 | hypothetical protein | 1.184444612 | 0.030243795 |
| Soltu.DM.12G003800 | conserved hypothetical protein | 1.186198432 | 0.030273883 |
| Soltu.DM.08G011280 | conserved hypothetical protein | 1.199904787 | 0.030596475 |
| Soltu.DM.03G036590 | SNARE-like superfamily protein | 1.220725339 | 0.030727216 |
| Soltu.DM.01G037990 | CTP synthase family protein | 1.262494988 | 0.030931704 |
| Soltu.DM.05G021790 | conserved hypothetical protein | 1.266946541 | 0.030999944 |
| Soltu.DM.05G016070 | hypothetical protein | 1.267850855 | 0.031005151 |
| Soltu.DM.04G004680 | Cox19-like CHCH family protein | 1.29390692 | 0.031339582 |
| Soltu.DM.12G009490 | Cytochrome P450 superfamily protein | 1.300377988 | 0.031549332 |
| Soltu.DM.12G000120 | receptor like protein | 1.307430372 | 0.031580559 |
| Soltu.DM.07G013260 | conserved hypothetical protein | 1.31703059 | 0.031657771 |
| Soltu.DM.09G023280 | TSPO(outer membrane tryptophan-rich sensory protein)-related | 1.326705182 | 0.031825781 |
| Soltu.DM.03G032230 | inositol polyphosphate kinase 2 beta | 1.339758291 | 0.031848537 |
| Soltu.DM.01G027220 | conserved hypothetical protein | 1.371813919 | 0.031875314 |
| Soltu.DM.04G031940 | hypothetical protein | 1.407027978 | 0.031908703 |
| Soltu.DM.03G024920 | cysteine-rich RLK (RECEPTOR-like protein kinase) | 1.409697093 | 0.032209432 |
| Soltu.DM.10G020380 | hypothetical protein | 1.41142523 | 0.032227722 |
| Soltu.DM.03G019600 | hypothetical protein | 1.413893429 | 0.032494038 |
| Soltu.DM.02G014590 | Nodulin MtN3 family protein | 1.428746845 | 0.032553318 |
| Soltu.DM.05G018930 | hypothetical protein | 1.434939811 | 0.032651216 |
| Soltu.DM.07G010710 | conserved hypothetical protein | 1.44154375 | 0.032724824 |
| Soltu.DM.02G020650 | conserved hypothetical protein | 1.47288861 | 0.032754889 |
| Soltu.DM.04G032290 | conserved hypothetical protein | 1.472916855 | 0.032899926 |
| Soltu.DM.01G031630 | Plant VAMP (vesicle-associated membrane protein) family protein | 1.485754836 | 0.032944559 |
| Soltu.DM.04G007430 | heat shock cognate protein 70-1 | 1.494308428 | 0.033049835 |
| Soltu.DM.01G023530 | pyruvate orthophosphate dikinase | 1.495902102 | 0.033387025 |
| Soltu.DM.06G019860 | 3-ketoacyl-CoA synthase | 1.505622702 | 0.033490548 |
| Soltu.DM.04G029640 | conserved hypothetical protein | 1.515464642 | 0.033536792 |
| Soltu.DM.01G038080 | Phosphoglycerate mutase family protein | 1.516069011 | 0.033967334 |
| Soltu.DM.12G004950 | hypothetical protein | 1.5177408 | 0.034269948 |
| Soltu.DM.12G001430 | conserved hypothetical protein | 1.524589846 | 0.034289179 |
| Soltu.DM.04G001420 | Endosomal targeting BRO1-like domain-containing protein | 1.563834372 | 0.034707156 |
| Soltu.DM.01G047240 | UDP-glucosyl transferase 73B3 | 1.605493394 | 0.034888855 |
| Soltu.DM.07G002680 | hypothetical protein | 1.632822201 | 0.035017818 |
| Soltu.DM.02G019680 | cysteine-rich RLK (RECEPTOR-like protein kinase) | 1.634544641 | 0.035313941 |
| Soltu.DM.01G047850 | conserved hypothetical protein | 1.635725189 | 0.035440613 |
| Soltu.DM.03G025850 | hypothetical protein | 1.637090284 | 0.035588554 |
| Soltu.DM.12G004590 | conserved hypothetical protein | 1.650217025 | 0.035613362 |
| Soltu.DM.07G016750 | ethylene-forming enzyme | 1.657403886 | 0.03566943 |
| Soltu.DM.04G028760 | hypothetical protein | 1.662263208 | 0.036095773 |
| Soltu.DM.12G004730 | Domain of unknown function (DUF23) | 1.669406132 | 0.036302693 |
| Soltu.DM.10G005750 | hypothetical protein | 1.671310425 | 0.036482096 |
| Soltu.DM.12G011610 | conserved hypothetical protein | 1.679331206 | 0.036554584 |
| Soltu.DM.06G023680 | hypothetical protein | 1.695003492 | 0.036663903 |
| Soltu.DM.01G033110 | NAD-dependent malic enzyme | 1.713034759 | 0.03672739 |
| Soltu.DM.11G009550 | Preprotein translocase SecA family protein | 1.71600896 | 0.036805907 |
| Soltu.DM.11G016270 | conserved hypothetical protein | 1.725882953 | 0.036887135 |
| Soltu.DM.03G033110 | Auxin efflux carrier family protein | 1.739170977 | 0.036918589 |
| Soltu.DM.04G032430 | hypothetical protein | 1.741078028 | 0.036925086 |
| Soltu.DM.08G023650 | hypothetical protein | 1.791952401 | 0.037322561 |
| Soltu.DM.08G009890 | conserved hypothetical protein | 1.814170285 | 0.037349607 |
| Soltu.DM.03G009790 | glutamate synthase | 1.816165263 | 0.037558672 |
| Soltu.DM.09G026100 | Target SNARE coiled-coil domain protein | 1.821486951 | 0.037700086 |
| Soltu.DM.06G017250 | hypothetical protein | 1.876615357 | 0.037858114 |
| Soltu.DM.12G021100 | cellulose synthase like E1 | 1.897680736 | 0.038007385 |
| Soltu.DM.09G030560 | Sar8.2 family domain containing protein | 1.903776097 | 0.038793333 |
| Soltu.DM.02G019320 | conserved hypothetical protein | 1.93099936 | 0.038813364 |
| Soltu.DM.10G002500 | Tyrosine transaminase family protein | 1.938425125 | 0.038913189 |
| Soltu.DM.10G002010 | Auxin-responsive GH3 family protein | 1.951492675 | 0.039094025 |
| Soltu.DM.12G002640 | UDP-D-glucuronate 4-epimerase | 2.019566001 | 0.039510494 |
| Soltu.DM.07G014280 | heat shock protein 70 (Hsp 70) family protein | 2.021117179 | 0.039792335 |
| Soltu.DM.11G021250 | conserved hypothetical protein | 2.02430918 | 0.039996645 |
| Soltu.DM.02G010160 | hypothetical protein | 2.03666751 | 0.040042864 |
| Soltu.DM.02G015350 | conserved hypothetical protein | 2.056506065 | 0.040612822 |
| Soltu.DM.08G029220 | conserved hypothetical protein | 2.082625452 | 0.040654643 |
| Soltu.DM.06G009730 | hypothetical protein | 2.100275257 | 0.040814635 |
| Soltu.DM.01G007940 | potassium channel in Arabidopsis thaliana | 2.12262803 | 0.040879667 |
| Soltu.DM.02G006390 | CAP-binding protein | 2.125231705 | 0.041138771 |
| Soltu.DM.09G012730 | hypothetical protein | 2.177795037 | 0.041217504 |
| Soltu.DM.01G016650 | hypothetical protein | 2.192073996 | 0.041296681 |
| Soltu.DM.06G029130 | hypothetical protein | 2.271409891 | 0.041314441 |
| Soltu.DM.04G006710 | hypothetical protein | 2.281534911 | 0.041495573 |
| Soltu.DM.12G007720 | hypothetical protein | 2.288860935 | 0.041554918 |
| Soltu.DM.01G001950 | hypothetical protein | 2.300951114 | 0.041661179 |
| Soltu.DM.12G025160 | AP2/B3-like transcriptional factor family protein | 2.339931063 | 0.041763336 |
| Soltu.DM.03G037490 | conserved hypothetical protein | 2.376144594 | 0.042078776 |
| Soltu.DM.04G027320 | cellulose synthase A2 | 2.388235331 | 0.042222204 |
| Soltu.DM.03G021550 | conserved hypothetical protein | 2.392055285 | 0.042295492 |
| Soltu.DM.12G011550 | ARF GTPase-activating protein | 2.401669016 | 0.042423569 |
| Soltu.DM.09G017090 | heat shock protein 18.2 | 2.407456085 | 0.042568539 |
| Soltu.DM.08G018120 | hypothetical protein | 2.454664965 | 0.042662339 |
| Soltu.DM.01G034180 | 1-amino-cyclopropane-1-carboxylate synthase | 2.612224142 | 0.042713686 |
| Soltu.DM.09G015430 | hypothetical protein | 2.631069049 | 0.043334444 |
| Soltu.DM.06G024280 | phospholipid N-methyltransferase | 2.678661698 | 0.043525087 |
| Soltu.DM.03G032300 | calnexin | 2.730352012 | 0.044125226 |
| Soltu.DM.11G005440 | UDP-Glycosyltransferase superfamily protein | 2.7353111 | 0.044159257 |
| Soltu.DM.05G027120 | PDI-like 1-1 | 2.847539615 | 0.044230265 |
| Soltu.DM.02G022830 | phosphoserine aminotransferase | 2.908166338 | 0.044238281 |
| Soltu.DM.09G012680 | conserved hypothetical protein | 2.931728735 | 0.044303919 |
| Soltu.DM.01G037800 | receptor like protein | 3.022109841 | 0.044578116 |
| Soltu.DM.08G027500 | Myosin heavy chain-related protein | 3.029543411 | 0.044731276 |
| Soltu.DM.04G028740 | hypothetical protein | 3.063136801 | 0.044735091 |
| Soltu.DM.06G009270 | phospholipase C | 3.06467609 | 0.044887869 |
| Soltu.DM.05G003040 | conserved hypothetical protein | 3.160579491 | 0.044907426 |
| Soltu.DM.07G005660 | ADP-ribosylation factor family protein | 3.168862073 | 0.04549568 |
| Soltu.DM.08G002280 | Uridine diphosphate glycosyltransferase 74E2 | 3.184711774 | 0.045552385 |
| Soltu.DM.12G013810 | conserved hypothetical protein | 3.246619567 | 0.045907449 |
| Soltu.DM.01G050210 | fructose-bisphosphate aldolase | 3.408074846 | 0.046124481 |
| Soltu.DM.07G025190 | Auxin-responsive GH3 family protein | 3.520661941 | 0.046162748 |
| Soltu.DM.02G029010 | conserved hypothetical protein | 3.578330695 | 0.046214453 |
| Soltu.DM.12G010140 | conserved hypothetical protein | 3.579942642 | 0.046565995 |
| Soltu.DM.08G002210 | Uridine diphosphate glycosyltransferase 74E2 | 3.604714926 | 0.046924001 |
| Soltu.DM.05G021730 | hypothetical protein | 3.702633123 | 0.047028466 |
| Soltu.DM.05G002210 | ATPase E1-E2 type family protein / haloacid dehalogenase-like hydrolase family protein | 3.764409369 | 0.0471839 |
| Soltu.DM.09G007110 | conserved hypothetical protein | 3.859860699 | 0.047417161 |
| Soltu.DM.12G010510 | hypothetical protein | 3.985016204 | 0.047682942 |
| Soltu.DM.12G010010 | conserved hypothetical protein | 4.01948416 | 0.04784213 |
| Soltu.DM.08G005970 | conserved hypothetical protein | 4.0852373 | 0.048098534 |
| Soltu.DM.03G027800 | Glycosyl hydrolase family 47 protein | 4.311623701 | 0.048257687 |
| Soltu.DM.05G025840 | conserved hypothetical protein | 4.373181515 | 0.048265547 |
| Soltu.DM.12G003440 | UDP-Glycosyltransferase superfamily protein | 4.475971861 | 0.04881218 |
| Soltu.DM.10G028370 | UDP-Glycosyltransferase superfamily protein | 4.500457959 | 0.048819734 |
| Soltu.DM.08G022700 | hypothetical protein | 4.562678484 | 0.048974863 |
| Soltu.DM.07G017850 | Transmembrane protein 97, predicted | 4.568364368 | 0.049728787 |
| Soltu.DM.06G000660 | hypothetical protein | 5.045771146 | 0.049932665 |
