# Supplementary material for: The Induction of Disease Resistance by Scopolamine and the Application of Datura Extract Against Potato (Solanum tuberosum L.) Late Blight
Source: Int J Mol Sci. 2024 Dec 15;25(24):13442. doi: 10.3390/ijms252413442 (PMC11676833; doi:10.3390/ijms252413442)
Supplement: Supplementary file 1 [file ijms-25-13442-s001.zip › Supplementary Table 9.docx]

**Supplementary** **Table 9 Effects of different treatments on potato yield**

| treatment group | total mass/kg | commercial potato quality/kg | commodity potato rate (%) | rate of increase (%) |
| --- | --- | --- | --- | --- |
| 1 | 62.37 | 30.66 | 49.16 |  |
| 2 | 85.56 | 50.31 | 58.80 | 37.18 |
| 3 | 76.29 | 38.88 | 50.96 | 22.32 |
| 4 | 63.09 | 38.10 | 60.39 | 1.15 |
| 5 | 80.52 | 48.18 | 59.84 | 29.10 |

Note: Duncan analysis was used for difference significance analysis. Different letters indicated significant difference between experimental results, and different lowercase letters indicated *P* < 0.05.
