# Supplementary figures and images for: The Induction of Disease Resistance by Scopolamine and the Application of Datura Extract Against Potato (Solanum tuberosum L.) Late Blight
Source: Int J Mol Sci. 2024 Dec 15;25(24):13442. doi: 10.3390/ijms252413442 (PMC11676833; doi:10.3390/ijms252413442)

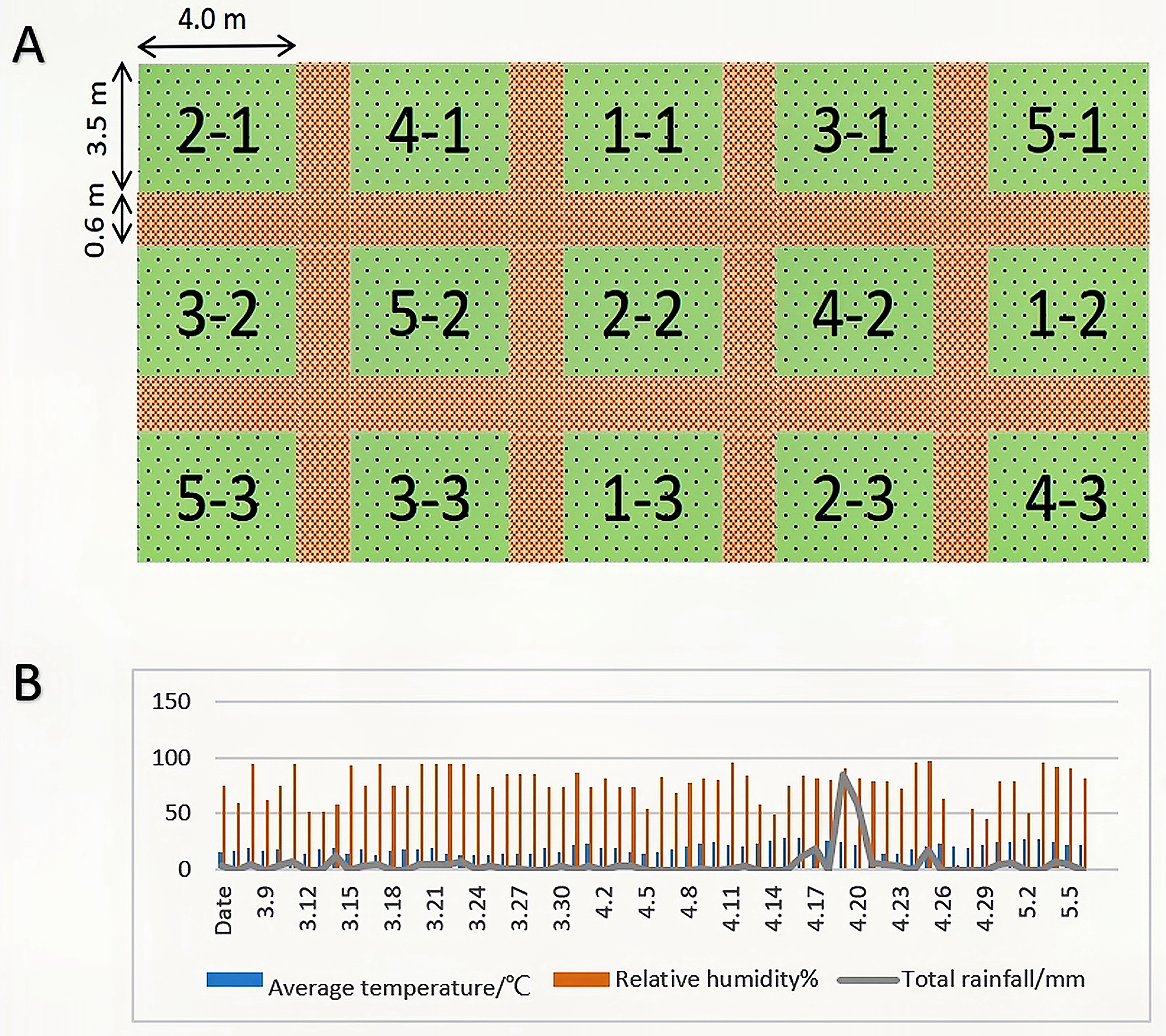

Supplement: Supplementary file 1 [file ijms-25-13442-s001.zip › Supplementary Figure 1.tif]

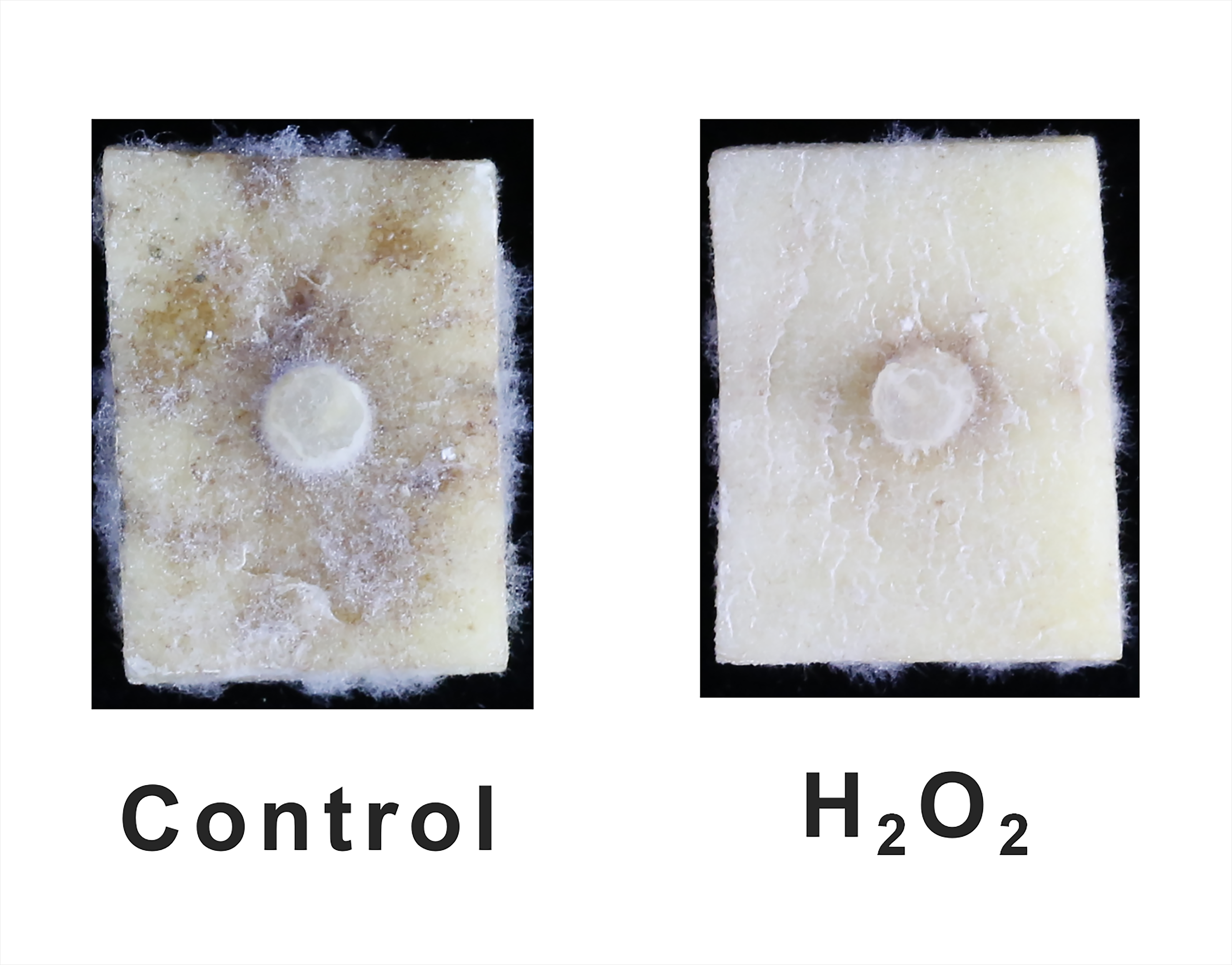

Supplement: Supplementary file 1 [file ijms-25-13442-s001.zip › Supplementary Figure 2.tif]
